# Genome-wide profiling of chromosome interactions in *Plasmodium falciparum* characterizes nuclear architecture and reconfigurations associated with antigenic variation

**DOI:** 10.1111/mmi.12381

**Published:** 2013-09-30

**Authors:** Jacob E Lemieux, Sue A Kyes, Thomas D Otto, Avi I Feller, Richard T Eastman, Robert A Pinches, Matthew Berriman, Xin-zhuan Su, Chris I Newbold

**Affiliations:** 1 Weatherall Institute of Molecular MedicineHeadington, Oxford, OX3 9DS, UK; 2 National Institute of Allergy and Infectious Disease, NIHRockville, MD, 20892, USA; 3 Wellcome Trust Sanger Institute, Wellcome Trust Genome CampusHinxton, Cambridge, CB10 1SA, UK; 4 Department of Statistics, Harvard University1 Oxford Street, Cambridge, MA, 02138, USA

## Abstract

Spatial relationships within the eukaryotic nucleus are essential for proper nuclear function. In *Plasmodium falciparum*, the repositioning of chromosomes has been implicated in the regulation of the expression of genes responsible for antigenic variation, and the formation of a single, peri-nuclear nucleolus results in the clustering of rDNA. Nevertheless, the precise spatial relationships between chromosomes remain poorly understood, because, until recently, techniques with sufficient resolution have been lacking. Here we have used chromosome conformation capture and second-generation sequencing to study changes in chromosome folding and spatial positioning that occur during switches in *var* gene expression. We have generated maps of chromosomal spatial affinities within the *P. falciparum* nucleus at 25 Kb resolution, revealing a structured nucleolus, an absence of chromosome territories, and confirming previously identified clustering of heterochromatin foci. We show that switches in *var* gene expression do not appear to involve interaction with a distant enhancer, but do result in local changes at the active locus. These maps reveal the folding properties of malaria chromosomes, validate known physical associations, and characterize the global landscape of spatial interactions. Collectively, our data provide critical information for a better understanding of gene expression regulation and antigenic variation in malaria parasites.

## Introduction

The eukaryotic nucleus is an intricately structured organelle whose organization is essential for normal cellular function. Physical space within the nucleus is a key determinant of many processes including transcription of ribosomal RNA within the nucleolus, activation and silencing of genes through interaction with enhancer and silencer elements, and the establishment of epigenetic memory through histone modifications and higher-order chromatin structures (Osborne *et al.*, 2004; Misteli, 2005; Sproul *et al.*, 2005; Ling *et al.*, 2006; Fudenberg and Mirny, 2012).

In the human malaria parasite, *Plasmodium falciparum*, nuclear substructure has been linked to antigenic variation, the major mechanism by which *P. falciparum* evades the host immune response and establishes a chronic infection. The parasite achieves this variation by the sequential expression on the red cell surface of different members of the *P. falciparum* Erythrocyte Membrane Protein 1 (PfEMP-1) family that are encoded by *var* genes (Baruch *et al.*, 1995; Smith *et al.*, 1995; Su *et al.*, 1995). Within the haploid genome, there are approximately 60 copies of *var* genes, the majority of which are located in subtelomeric regions with a few clusters located at internal chromosomal regions. The *var* genes are expressed in a mutually exclusive manner; a single locus is transcribed while the remaining copies of the gene family remain silenced within facultative heterochromatin localized at the nuclear periphery (Duraisingh *et al.*, 2005; Freitas-Junior *et al.*, 2005). Activation of a *var* gene involves movement away from a silenced cluster and into a transcriptionally permissive region (Ralph *et al.*, 2005; Dzikowski *et al.*, 2007; Guizetti *et al.*, 2013), suggesting a change in chromatin interaction associated with the control of gene expression.

Another example of the influence of nuclear position on gene expression in *P. falciparum* is the ribosomal RNA genes that have recently been shown to cluster in a peri-nuclear nucleolus. Their presence in this region constrains the interaction preferences of the telomeres of *P. falciparum* chromosomes containing rDNA elements (Mancio-Silva *et al.*, 2010). Despite these clearly described position effects, chromosomes of *P. falciparum* are unusual in that they do not condense to visible higher-order structures during mitosis (Bannister *et al.*, 2000; Gerald *et al.*, 2011), although a number of organisms, including *S. cerevisiae*, do not demonstrate observable chromosome condensation during mitosis (Heath, 1980; Winey *et al.*, 1995).

Studies of nuclear localization in *P. falciparum* have typically relied on fluorescence *in situ* hybridization (FISH). While these investigations have successfully defined a link between *var* gene activation and spatial positioning, FISH has major limitations including a resolution that is limited by the wavelength of light and the need to label a sufficiently large segment of a chromosome to visualize with fluorescence microscopy. The number of fluorophores that can be spectrally distinguished also restricts throughput. Therefore, while studies using FISH have yielded great insight into the spatial behaviour of a small number of genes, little is known about the spatial positioning of genes at a whole-genome level.

The chromosome conformation capture (3C) assay (Dekker *et al.*, 2002) offers a complementary way to detect spatial interactions in the nucleus by biochemical cross-linking, restriction digestion and subsequent ligation of distal sequence fragments. The resulting DNA library contains three-dimensional spatial information converted into one-dimensional DNA sequence. When first introduced, 3C libraries were analysed using PCR- or microarray-based methods, but these techniques have been able to recover only a portion of information present in such libraries. The recent advent of second-generation sequencing allows direct analysis of 3C libraries at a scale sufficient to reveal much of the complexity of DNA interactions in the nucleus (Lieberman-Aiden *et al.*, 2009; Rodley *et al.*, 2009). The approach of massively parallel sequencing of 3C-type libraries has transformed our understanding of the physical properties of chromatin and the biological relevance of subnuclear spatial relationships (Fullwood *et al.*, 2009; Duan *et al.*, 2010; Yaffe and Tanay, 2011; Sexton *et al.*, 2012).

In this work, we use direct sequencing of 3C libraries (also known as Genome Conformation Capture, GCC) (Rodley *et al.*, 2009), as well as HiC, a similar procedure in which biotin is incorporated into the ligation site (Lieberman-Aiden *et al.*, 2009), to generate genome-wide maps of spatial interaction and chromosome folding in *P. falciparum* with particular emphasis on *var* gene expression. Sequencing the 23 Mb *P. falciparum* genome to high coverage allowed us to generate maps with a bin size of 25 Kb (MboI) and 50 Kb (HindIII).

The mutually exclusive expression of *var* genes shares many features with the mammalian olfactory receptors. Both establish reversible, mutually exclusive expression of a single member of a large gene family within a single cell. Olfactory receptors, like *var* genes, enforce this mutually exclusion expression in part through heterochromatin (Magklara *et al.*, 2011) and subnuclear localization (Clowney *et al.*, 2012). The olfactory receptor genes are additionally regulated by enhancer elements, including the H-element (Serizawa *et al.*, 2003; Lomvardas *et al.*, 2006) and the P-element (Bozza *et al.*, 2009; Khan *et al.*, 2011), but it is not known whether similar enhancer elements exist in *P. falciparum*. Although the H-element was initially believed to be a single, pan-enhancer, more recent data suggest that both the H- and P-elements are involved in activating a particular cluster of OR genes (Lomvardas *et al.*, 2006; Fuss *et al.*, 2007; Khan *et al.*, 2011). We tested the hypothesis that mutually exclusive expression of the active *var* gene is maintained by associated enhancer elements similar to the H-elements and P-elements in the mammalian olfactory receptor gene family. We observed reorganization in local chromatin structure associated with *var* gene switching, but were not able to identify any specific, long-range associations suggestive of a trans-acting enhancer or a long-range cis-acting enhancer. The maps provide the first global view of the *P. falciparum* nucleus, define the basic folding properties of malaria parasite chromosomes, reveal a substructured nucleolus which maintains physical associations between active rDNA loci, document an apparent absence of chromosome territories, and profile the changes associated with *var* switching.

## Results

### Generation of chromosome conformation capture libraries in lines expressing single PfEMP-1 proteins

**Fig 1:**
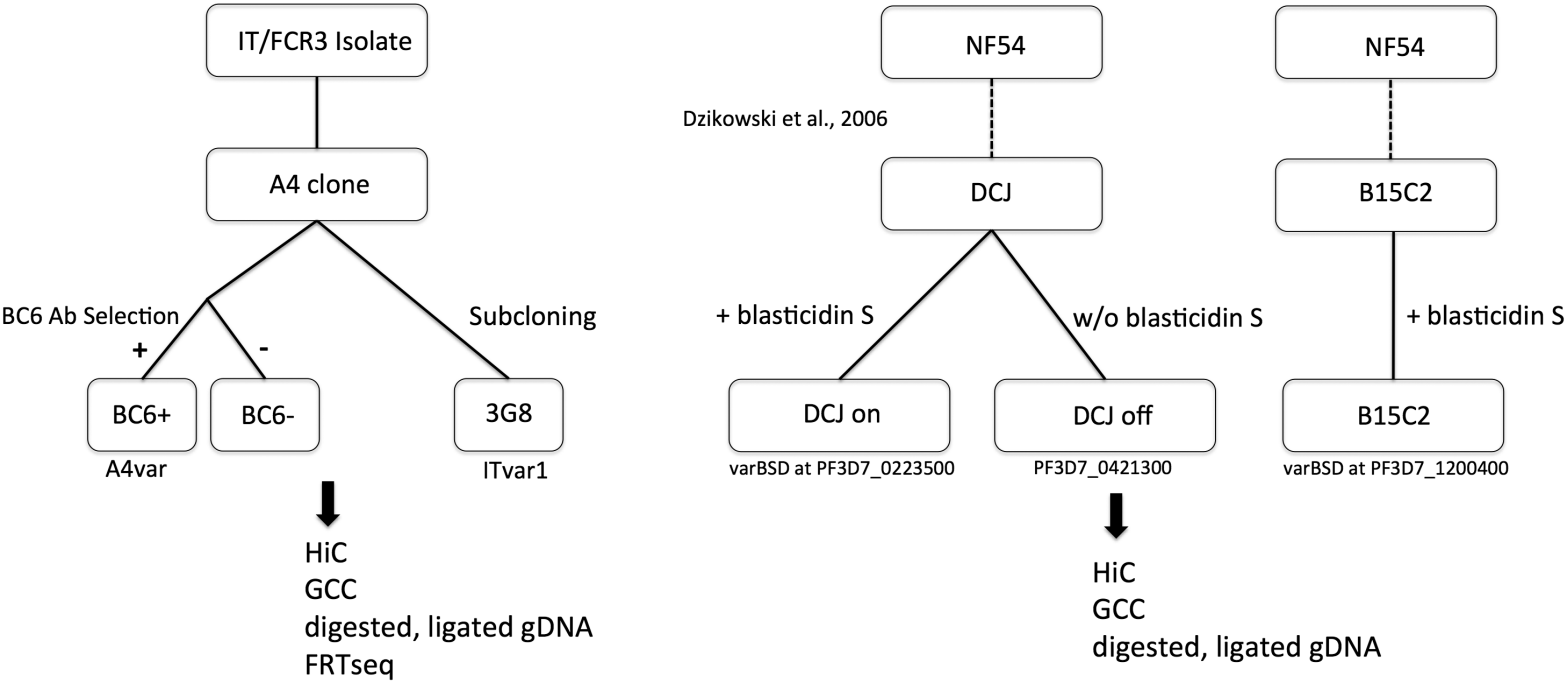
Generation of parasite clones used in this study and the experiments performed on them. The BC6+ and BC6− cultures were obtained by positive and negative selection of the A4 subclone of the IT isolate with BC6 monoclonal antibody (see *Experimental procedures*). The 3G8 subclone was generated previously (clone F in Horrocks *et al.*, 2004). The IT clones (A4 and 3G8 subclones) were then subjected to GCC and HiC using MboI and HindIII, along with contemporaneous RNAseq (FRTseq) (Mamanova *et al.*, 2010) and digested, ligated genomic DNA control libraries. The DCJ and B15C2 lines were created by Dzikowski *et al*. in reference (Dzikowski *et al.*, 2006) and contain a chromosomally integrated blasticidin S deaminase gene, enabling rapid selection of distinct *var* loci. These parasite isolates were synchronized, grown to late ring stage, and subjected to GCC and HiC using MboI and HindIII. Overall, among the A4 clones, a BC6-positively selected population, a BC6-negatively selected population, and a 3G8 subclone were subjected to HiC, GCC, control library preparation and FRTseq, while among the NF54-derived lines, a line of DCJ on blasticidin, a line of DCJ off blasticidin and a B15C2 line were subjected to HiC, GCC and control library preparation.

In order to study the general features of chromosome folding in *P. falciparum* and the specific contacts associated with unique *var* gene expression, we generated several populations of parasites that were selected for homogenous expression of single *var* genes. The generation of these lines is summarized in Fig. 1. Using a monoclonal antibody (BC6) that specifically recognizes the extracellular portion of the protein product of the A4*var* gene (Smith *et al.*, 1995), located in a subtelomeric region of chromosome 13, we performed positive and negative selection, resulting in lines expressing A4*var* PfEMP-1 (BC6+) and lines lacking A4*var* PfEMP-1 expression at the RBC surface (BC6−). We also studied a subclone of A4, known as 3G8, which has undergone a switch in the expressed *var* gene and stably expresses PfEMP-1 from a central *var* gene (Horrocks *et al.*, 2004). The expression of the *var* genes in these parasites was evaluated using flow cytometry and Northern blot (Fig. S1). Lines were then synchronized and maintained in culture until mid-to-late ring stage when they were harvested for GCC, HiC and strand-specific RNA sequencing. A single time point was used in order to focus on mechanisms governing *var* gene regulation (Kyes *et al.*, 2007) and to remove the confounding effect of stage-specific gene expression and chromatin condensation; equivalent temporal development was confirmed (Fig. S1D and E) by a previously developed method to infer temporal development from observed gene expression profiles (Lemieux *et al.*, 2009).

For these experiments, the *P. falciparum* IT lineage was used because it allows phenotypic selection of parasites expressing a single, specific *var* gene in isolates without genetic modification. For second-generation sequencing-based assays, which generate large numbers of short reads that are mapped back to a reference genome, the quality of the underlying reference is crucial in obtaining reliable results. An improved reference sequence has recently been produced by the Wellcome Trust Sanger Institute (ITv2 available at http://www.genedb.org). We made additional improvements to this sequence (see *Experimental procedures*) to produce ITv2.5. Nevertheless, the IT genome sequence is still incomplete in many low complexity and polymorphic regions, due to limitations of current short read technology. We therefore sought to conduct a parallel set of experiments using NF54, the parent of clone 3D7 for which complete, telomere-to-telomere sequence is available for all 14 chromosomes.

To this end, we made use of the selectable-marker system developed by Deitsch and colleagues in the NF54 background (Dzikowski *et al.*, 2006). DCJ parasites contain a blasticidin S deaminase gene (*BSD*) integrated in place of exon 1 of PF3D7_0223500, a subtelomeric *var* gene on chromosome 2. After integration, subcloned DCJ parasites, grown in the absence of blasticidin S, express the chromosome 4 internal *var* gene PF3D7_0421300. Before addition of blasticidin S to the culture, the *var BSD* locus remains silent in these parasites. When grown in the presence of drug, parasite populations that all express *var BSD* can be rapidly selected. We grew DCJ lines in the absence and presence of blasticidin S for a period of 4 weeks and confirmed their *var* expression profiles by quantitative, real-time PCR (Fig. S2). We also cultured a third parasite under blasticidin S pressure, B15C2 (also generated by Dzikowski *et al.*, 2006), which possesses an integrated *BSD* locus between the promoter and first exon of PF3D7_1200400, a *var* gene located in a subtelomere of chromosome 12. After confirming *var* gene expression, these three parasite lines were tightly synchronized, grown until late ring stage, and subjected to GCC and HiC using both HindIII and MboI. Purified genomic DNA from the 3D7 parasite clone was also digested, ligated and sequenced as a control for these experiments.

### Analysis strategy

Chromosome conformation capture establishes structural information about chromosomes by estimating the relative frequency with which loci interact. These estimates of interaction frequency are derived from the number of split read-pairs, i.e. read-pairs that map either greater than 2 Kb further apart on the same chromosome, or on separate chromosomes. These estimates can be influenced by the experimental protocol as well as choices made in the analysis pipeline, including the reference genome, mapping strategy, normalization procedures and sequence homology. Therefore, before proceeding with the analysis, we studied each of these factors in detail in order to achieve an analysis strategy that reliably discovered genuine interactions while minimizing the number of false positives.

First, we compared interaction matrices derived from GCC and HiC libraries, using HindIII and MboI, and mapped under different alignment conditions (discussed in detail in Supplementary Note 1). In this analysis, we found that increasing the frequency of restriction sites (using a restriction enzyme with a four base recognition sequence) and enriching for split read-pairs with a biotin in the ligation site (HiC) could improve the read depth and resolution and reduce the noise of the interaction data set. As a result, we obtained the best quality data in the MboI HiC libraries.

We also found that homologous sequences within the malaria genome, particularly in subtelomeres, were much more likely to generate false-positive interactions. This effect could be removed by either discarding non-uniquely aligning reads or modelling the expected interaction due to homology using the read counts in control libraries (Supplementary Note 1 and Fig. S4). Discarding the reads that map to multiple places was the simplest and most conservative approach, but it had the effect of reducing statistical power in regions that contain homologous sequences. In contrast, modelling the expected signal retained more statistical power in these regions but is contingent on the validity of modelling assumptions. For the IT libraries, where our statistical power was limited in telomeric regions due to the underlying reference, we used the approach of discarding reads that map to multiple places in the reference. For the NF54-derived libraries, where the high-quality reference sequence allowed us to perform a more in-depth analysis of interactions in these regions, we generated corrected interaction maps by modelling the expected contribution of homology based on read counts in the control libraries (‘corrected’ maps).

### Global features of the chromatin interaction maps

**Fig 2:**
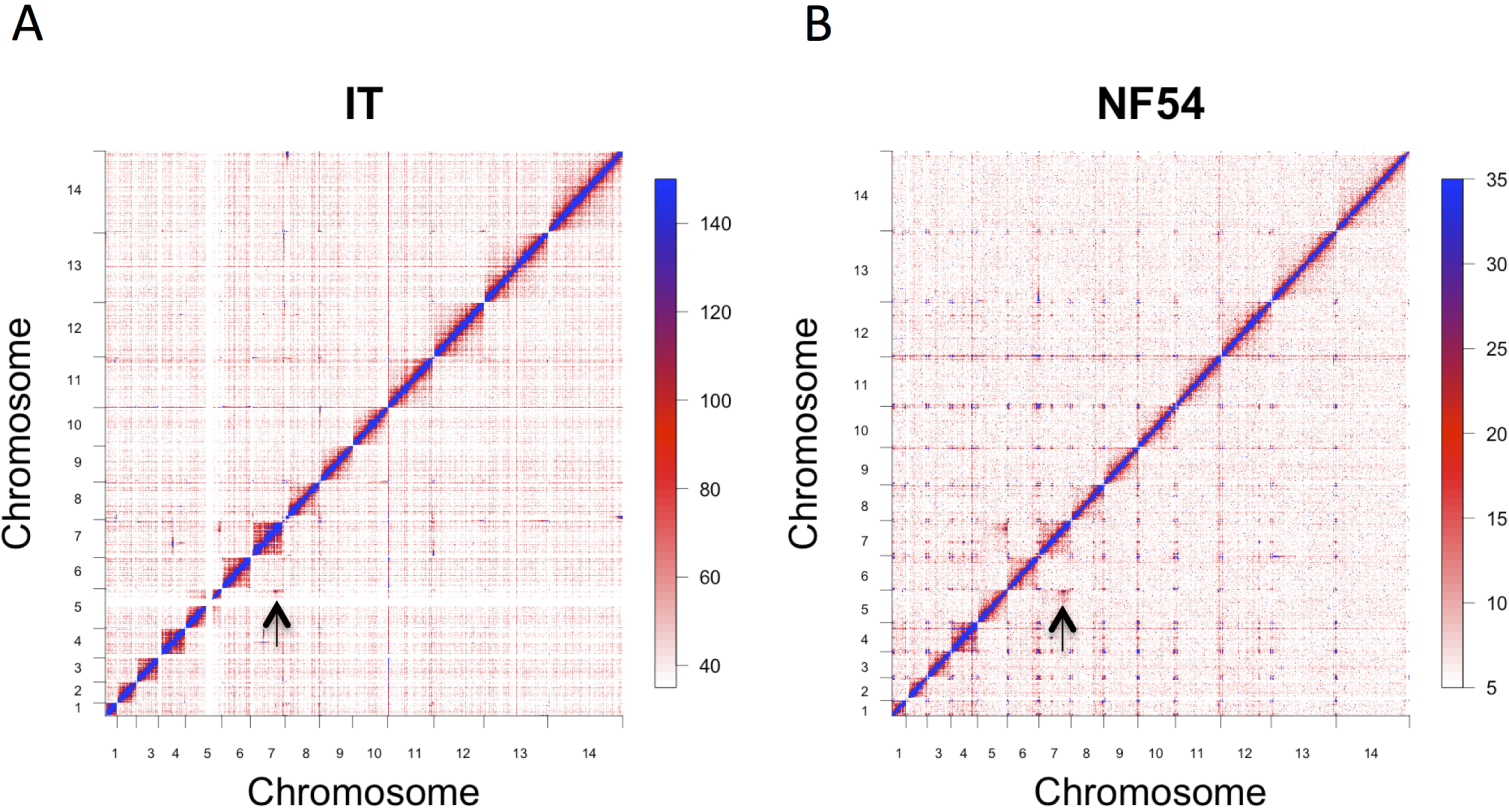
HiC interaction matrices for all 14 chromosomes constructed using the MboI enzyme with a bin resolution of 25 Kb in the clones descended from IT (A) and in the NF54 isolate (B). The value in matrix element A_ij_ corresponds to the number of reads we sequenced for which one mate corresponded to bin i and its mate pair to bin j. The matrices are by nature symmetric (i.e. A_ij_ = A_ji_), reflecting the fact that an interaction is an underlying contact between two loci without inherent direction. The clones have been combined in order to provide the highest signal to noise ratio. The full genome is shown with chromosomes 1 through 14 concatenated in numerical order. In (A) reads were not mapped to repetitive regions, which is why there are some gaps; for example the white space on chromosome 5 is due to a corresponding amplification at the MDR1 locus which has resulted in three copies of an approximately 100 Kb region. In (B), degenerately mapping reads were placed randomly (maps for the NF54 clones with degenerate reads unmapped, as for the IT clones in (A), are given in Fig. S4). In order to correct for spurious interactions that resulted from homology, matrix elements above the 99% percentile of the digested, ligated gDNA control libraries were set to zero in the HiC library (Supplementary Note 1). The genome-wide interaction matrices display a decrease in interaction as a function of linear distance within chromosomes, characteristic of polymers, interactions between telomeres and internal antigenic clusters, as well as a strong interaction between the region of chromosome 5 and 7 containing the A-type rDNA which is expressed in asexual stages [marked by the arrow in (A) and (B)].

The whole-genome interaction maps for IT and NF54 lines, constructed using HiC with the MboI enzyme, are shown in Fig. 2. These maps demonstrate reproducible patterns of chromosome interaction that are broadly equivalent in both parasite genotypes, including non-specific collisions that scale with distance due to the polymeric nature of DNA, interactions between rDNA elements, and contacts between heterochromatin clusters. Each of these interaction types is analysed in detail below.

**Fig 3:**
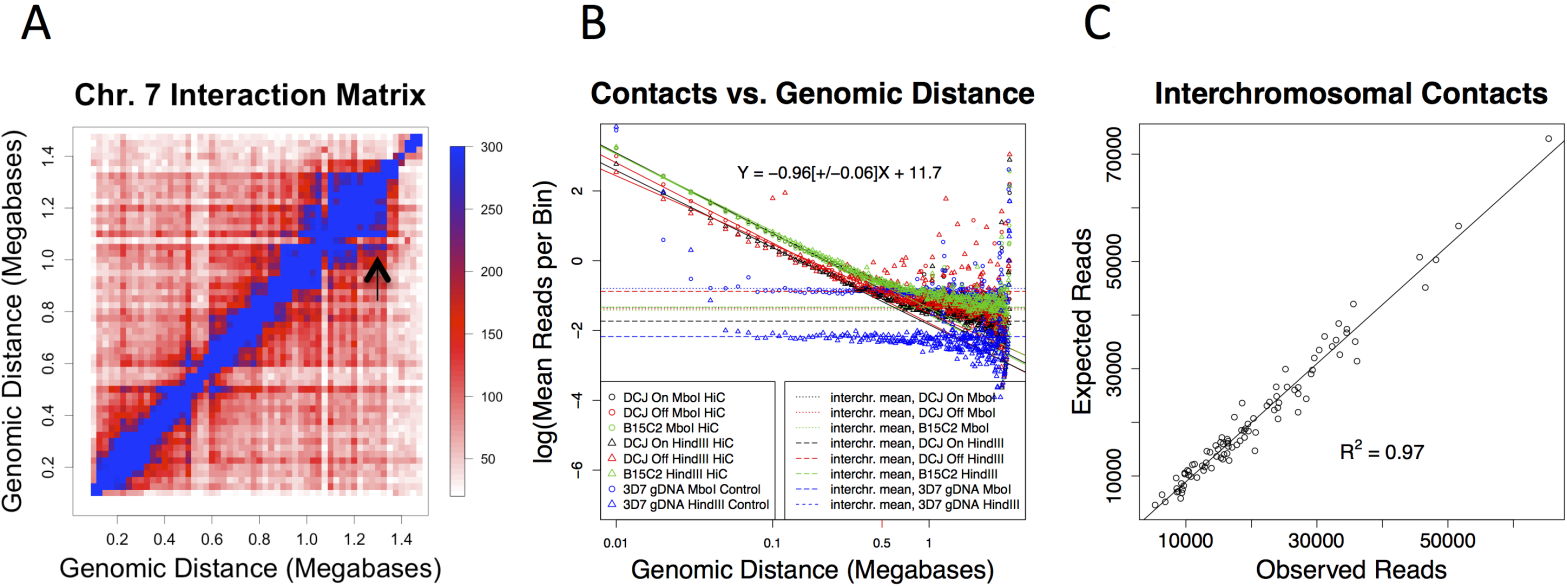
Scaling of interaction probability as a function of distance. (A) The interaction matrix, plotted as a heatmap for chromosome 7, from the combined A4 libraries. A decrease in interaction probability is seen as linear distance increases, a relationship quantified for the entire genome in (B), which plots the mean number of contacts observed at a given genomic distance (in base pairs) on log–log axes (note: all logarithms here are to the base e). A power law with scaling exponent of −0.96 (± 0.06) for the HiC IT libraries holds from 10 Kb to approximately 500 Kb (0.5Mb is marked on the *x*-axis in red). The linear relationship on log–log plots does not hold for random ligation libraries generated by digesting and ligating purified genomic DNA (blue points). Mean inter-chromosomal interaction counts are shown in dashed horizontal lines. The arrow in (A) marks a region of locally increased interaction density, as noted in the main text. In (C), the expected number of reads under a model in which the probability of making a contact between two chromosomes is proportional to the product of their size is compared with the observed number of contacts.

As expected, both in the whole-genome interaction matrices and in the interaction matrices for individual chromosomes, the most striking feature of the plots is a prominent diagonal line, representing interactions between reads from adjacent or linearly proximal segments of the chromosome. The interaction matrices for a single chromosome, chromosome 7, are shown in Fig. 3A. This pattern is consistent across chromosomes (Fig. 2 and Fig. S4A and B) and underscores the importance of the statistical and physical behaviour of polymers in shaping the contact landscape of the nucleus. Regions nearby on the same chromosome are more likely to interact in three-dimensional space simply because they are constrained to be nearby one another by the polymer backbone. At fine scale, the on-diagonal interactions that decayed as a functional of linear distance were the major feature of both GCC and HiC libraries, suggesting an absence of prominent intra-chromosomal looping, and suggesting a major role for entropic forces acting on polymer constraints in dictating the way that chromosomes interact. We observed modest variations in regional interaction density such as the one at the end of chromosome 7 (Fig. 3A – marked with an arrow, and discussed further below). Such regional variations were present on multiple chromosomes and were reproducible in our assays across replicates (Fig. S4A and B). The interaction matrices generated under different conditions (Fig. S4C–G) were quantitatively and qualitatively equivalent (average pairwise *r* = 0.99 for MboI HiC libraries from the IT isolates and *r* = 0.98 from the NF54 isolates). There was a strong correlation between GCC and HiC libraries for both enzymes used (MboI, *r* = 0.97 and HindIII, *r* = 0.88). Qualitatively, the major features of genomic architecture including the interaction between rDNA loci, between heterochromatin foci and within a relatively unstructured euchromatin genome were present in libraries constructed with both genotypes, via GCC or HiC, and using either HindIII or MboI (described below). Maps produced using NF54 benefitted from a completed reference genome, whereas greater read depth was available for maps constructed in the IT lineage, leading to maps with less noise in areas where the IT reference genome is intact (Fig. 2A and B).

### Intra-chromosomal interaction and contact probability

The quantitative scaling of contact probability as a function of the linear distance along a chromosome can provide insight into physical and statistical aspects of polymer folding (Lieberman-Aiden *et al.*, 2009; see also Supplementary Note 2). The number of contacts at a given distance is expected to follow a power law. We assessed the interaction probability by plotting the mean number of interaction pairs as a function of genomic distance on a log–log scale, and found a prominent power law relationship at distances between 10 Kb and 500 Kb (Fig. 3B and S5). We consistently obtained estimates of ∼ −1 for the slope of this graph (−0.96 ± 0.06 for MboI HiC libraries – Fig. 3B). The libraries produced with MboI and HindIII produced nearly identical curves (Fig. 3B and S5) despite different digestion fragment sizes. In contrast, control libraries made from parasite genomic DNA that was purified prior to digestion and ligation exhibited markedly different behaviour, with an exponential decrease over the first few bins followed by a line with zero slope at distances above 50 Kb (Fig. 3B and S5). The observed value of ∼ −1 for the power law exponent suggests an increase in contact probability relative to a freely diffusing polymer in an ideal solvent at equilibrium, which has a scaling exponent of −3/2, a result which comes from modelling a polymer as a three-dimensional random walk (Supplemental Note 2). The estimate of −1 for malaria chromosomes is similar to that obtained for human chromosomes (Lieberman-Aiden *et al.*, 2009), a finding which led the authors to invoke a folding pattern for human chromosomes known as the ‘crumpled’ or ‘fractal’ globule (Grosberg *et al*., 1988; 1993). The results we obtained for malaria chromosomes suggest that this scaling exponent may be a feature of chromosomes conserved among different species, although a general model of nuclear organization is still an area of active investigation (McNally and Mazza, 2010; Iyer *et al.*, 2011; Fudenberg and Mirny, 2012).

### Inter-chromosomal interactions

#### Chromosome territories and inter-chromosomal interactions

We compared the observed frequency of inter-chromosomal interactions to the frequency of intra-chromosomal interactions at large chromosomal distances as a way to assess whether chromosomes exist in distinct ‘territories’ (Branco and Pombo, 2006; Lieberman-Aiden *et al.*, 2009). In all sets of libraries studied, the intra-chromosomal interaction frequency decreased as a function of distance, until at approximately 1.5 Mb, the likelihood of making a contact between chromosomes was equivalent to that of making a contact within the same chromosome, indicating an absence of chromosome territories in *P. falciparum* (Fig. 3B and S5). This was in contrast to a human lymphoblastoid cell line in which the curves for intra- and inter-chromosomal contact probability did not converge, consistent with the presence of chromosome territories. Nevertheless, the size of most chromosomes in *P. falciparum* is less than 1.5 Mb; so most chromosomal loci are more likely to interact with elements of the same chromosome than with another chromosome, a situation that might be described as ‘effective’ or ‘statistical’ chromosome territories for smaller chromosomes. We also assessed whether chromosomes have preferences for one another. There was a strong linear relationship (*r* = 0.98, *P* < 10^−16^) between observed interactions and interactions predicted under a random model in which contact is proportional to the multiplied size of the two interaction chromosomes (Fig. 3C and Fig. S3F–H). Under this model, 97% of the variation in inter-chromosomal contacts can be accounted for by chromosome size alone, suggesting that inter-chromosomal contacts are dominated by probabilistic forces such as diffusion.

#### Interaction of rDNA loci

**Fig 4:**
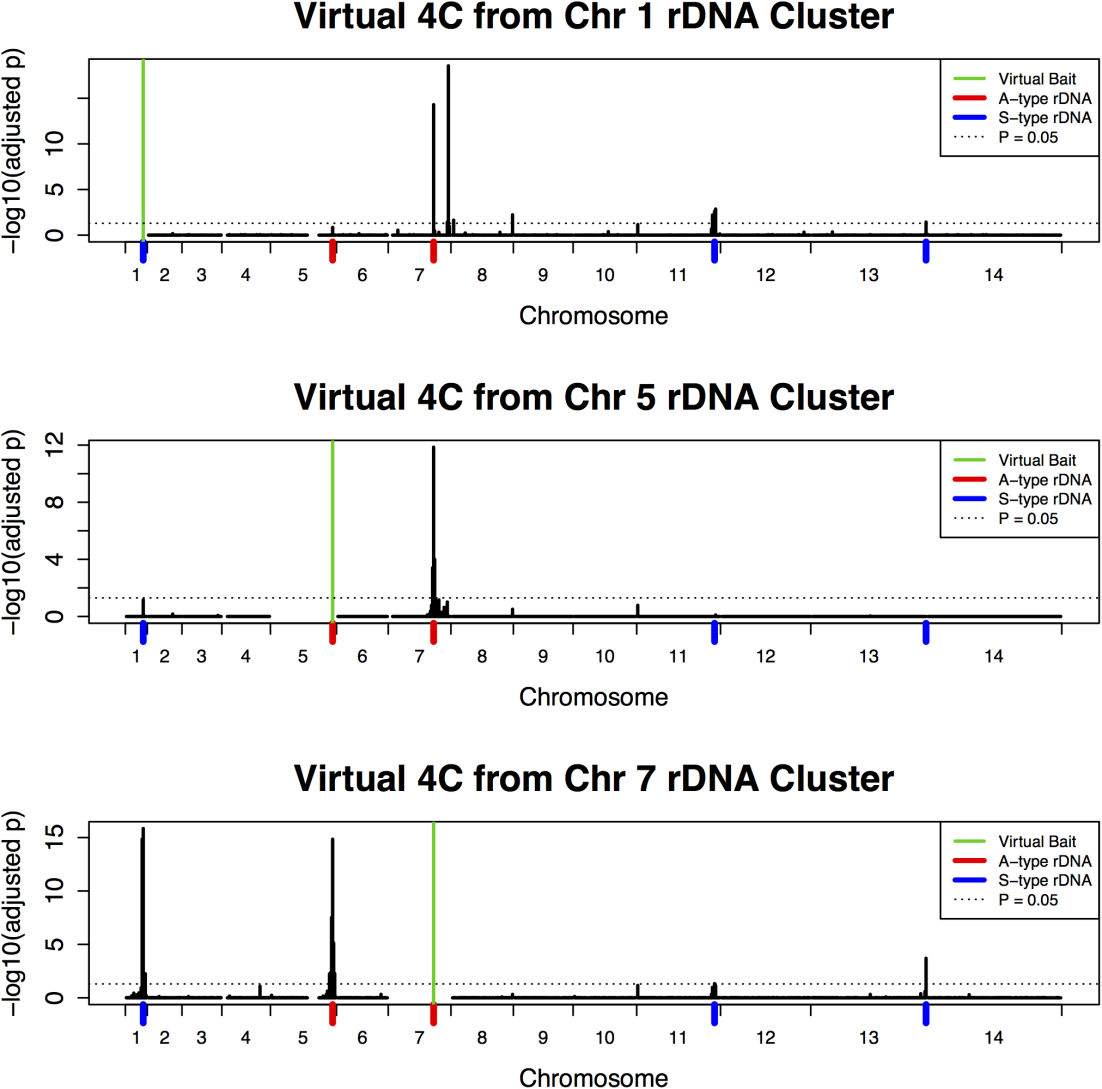
Interactions among rDNA clusters. Virtual 4C experiments from the rDNA loci on chromosome 1, 5 and 7, in which the interactions between a single bin (‘virtual bait’) and the remainder of the genome are plotted. The figure shows *P*-values; the interaction counts are given in Fig. S6. The *P*-values test the null hypothesis that the number of interactions in a given bin results from the background distribution of inter-chromosomal interactions (*Experimental procedures*). The dashed, black, horizontal line gives the value of *P* = 0.05. The rDNA cluster on chromosome 7 had statistically significant interactions with the other A-type rDNA cluster on chromosome 5 (*P* = 1.4 × 10^−15^) as well as the three S-type rDNA clusters on chromosomes 1, 11 and 13 (*P* = 1.4 × 10^−16^, 0.05 and 2.0 × 10^−4^ respectively), whereas the A-type cluster on chromosome 5 had significant interactions only with the cluster on chromosome 7 (*P* = 1.4 × 10^−12^) and a weak interaction with the cluster on chromosome 1 (*P* = 0.07), suggesting that, of the A-type rDNA loci, the one on chromosome 7 remains in better contact with the S-type rDNA loci may serve as a nucleolar hub. The pooled A4 MboI HiC libraries were used in this analysis because the large number of reads provided the strongest differentiation between signal and background noise, but results were similar for the NF54 libraries and the signal could also be seen in HindIII libraries and libraries constructed with GCC (Fig. S4).

There are two major types of inter-chromosomal interactions visible in the whole-genome interaction matrices shown in Fig. 2. The first is between telomeres and internal clusters of *var* genes, consistent with the known clustering of these sites in heterochromatin foci at the nuclear periphery (Freitas-Junior *et al.*, 2000) (discussed below). The second is the interactions between rDNA loci. Figure 4 shows plots of a virtual version (Sexton *et al.*, 2012) of a 4C experiment (Simonis *et al.*, 2006; Zhao *et al.*, 2006), a technique that evaluates the interactions between individual loci and the remainder of the genome. We observed significant increases in interaction among rDNA loci (Fig. 4, Fig. S6A; the A4 MboI HiC libraries were used for this analysis because they possessed the greatest number of reads and therefore the best ability to distinguish signal from noise, but the results described did not depend on library type, enzyme or strain, see Fig. S4C–G). The rDNA elements in the *P. falciparum* genome are arrayed as single rDNA 18S–5.8S–28S units on chromosomes 1, 5, 7, 11 and 13, with those clusters on chromosome 5, and 7 transcribed predominantly during the asexual stages (‘A-type’) while those on 1, 11 and 13 are transcribed during the sexual stages (‘S-type’) (Langsley *et al.*, 1983; Waters *et al.*, 1989; Gardner *et al.*, 2002). The interactions were particularly strong among the rDNA locus on chromosome 5 and the rDNA locus on chromosome 7 (*P* = 1.38 × 10^−12^, see *Experimental procedures* for an explanation of the testing and multiple correction procedure). The A-type rDNA genes are transcribed during the asexual stage, suggesting that active genes colocalize in the nucleolus. These interactions present as regional ‘peaks’ in the virtual 4C data (Fig. 4, and shown in higher resolution in Fig. S6B), not simply as elevated single bins in both IT and NF54-derived isolates, which is strong evidence of a genuine interaction since the decay of contact probability as a function of linear distance is characteristic of polymers interacting in space. As mentioned above and in Supplementary Note 1, homology between regions of the genome, such as rDNA clusters, has the potential to generate artificial interactions due to incorrect base calls and sequencing artefacts. However, this is unlikely to be the case, as reads that did not align uniquely were discarded and also because the interaction signal extended into adjacent regions that did not contain rDNA sequence. In addition we compared the interaction data from the GCC/HiC and control libraries which confirmed that the observed signals were not due to underlying sequence homology (Figs S4 and S6D).

Physical interactions between rDNA loci have been detected previously using FISH (Mancio-Silva *et al.*, 2010), indicating that the GCC approach can sensitively detect genuine interactions. Notably, the preferential interaction between the A-type genes was not apparent at the resolution of FISH data suggesting that GCC-based approaches can provide a level of detail beyond what is available by FISH. We further explored the spatial relationships within the *Plasmodium* nucleolus by using the specific interaction values for the rDNA loci (Fig. S6E) to generate a model of relative distances. To do this, we used multidimensional scaling (MDS) (Fig. S6F), a statistical technique that finds a best-fit approximation of distances using a numerical measure of similarity (in this case, contact probability). The relationships among rDNA elements reveal a nucleolus with substantial substructure. The most frequent spatial contacts are between actively expressed A-type rDNA. There is some level of contact between A- and S-type rDNA on chromosomes 1 and 11 and reduced contact between the S-type rDNA on chromosome 13 and other loci, suggesting that this latter locus may be distinct from the other two (Fig. 4 and Fig. S6E and F).

The interaction between A-type loci on chromosomes 5 and 7 is discernible above background for almost the entire section of chromosome 7 between the rDNA locus and the telomere, a region consisting of several hundred kilobases. As noted earlier, the intra-chromosomal contact probability in this region on chromosome 7 is also elevated, an effect which can be seen as the ‘block’ pattern along the diagonal (Fig. S3A – also visible in Fig. 2A and B and Fig. S6C). This confirms that nucleolar interactions impact spatial relationships in the non-rDNA-containing regions by tethering portions of the chromosome in the nucleolus as suggested by Mancio-Silva *et al.* (2010). The pattern is not uniform among chromosomes however with chromosome 7 showing the strongest effect. Whether this reflects a more stringent tethering of chromosome 7 within the nucleolus in space or in time, or some physical or biological feature of the adjacent chromosome is not clear. Some evidence for the former possibility comes from the relative frequency of interaction between the chromosome 7 rDNA locus and other rDNA loci. The A-type rDNA on chromosome 7 interacts frequently with both the S-type loci on Chr 1, Chr 11 and Chr 13, and the other A-type locus on chromosome 5 (Fig. 4). Such a pattern is not shared by other rDNA loci, suggesting that the chromosome 7 locus may be more centrally anchored within the nucleolar space than other rDNA loci during the ring stage (Fig. 4 and Fig. S6E and F). Finally, we note that the largest difference between the observed and expected inter-chromosomal interactions values (Fig. S3H) was between chromosome 5 and chromosome 7, consistent with the hypothesis that clustering of A-type rDNA loci in the nucleolus is one of the major non-random organizing forces of the nucleus during ring stage.

In contrast to rDNA loci, we did not find evidence of increased interaction among centromeres (Fig. S6G). This is consistent with data showing an absence of centromere clustering prior to the end of ring stage/beginning of trophozoite stage (Hoeijmakers *et al.*, 2012), a developmental landmark unlikely to have been reached in the parasites in our study (estimated at approximately 11 h post invasion from the expression data, Fig. S1D).

Overall, the interactions among rDNA loci identify important substructure in the nucleolus, confirm the role of this structure in constraining chromosome positioning in the nucleus at large and identify an association between contact probability and gene expression that links nucleolar physical space to gene activation.

#### Heterochromatin

**Fig 5:**
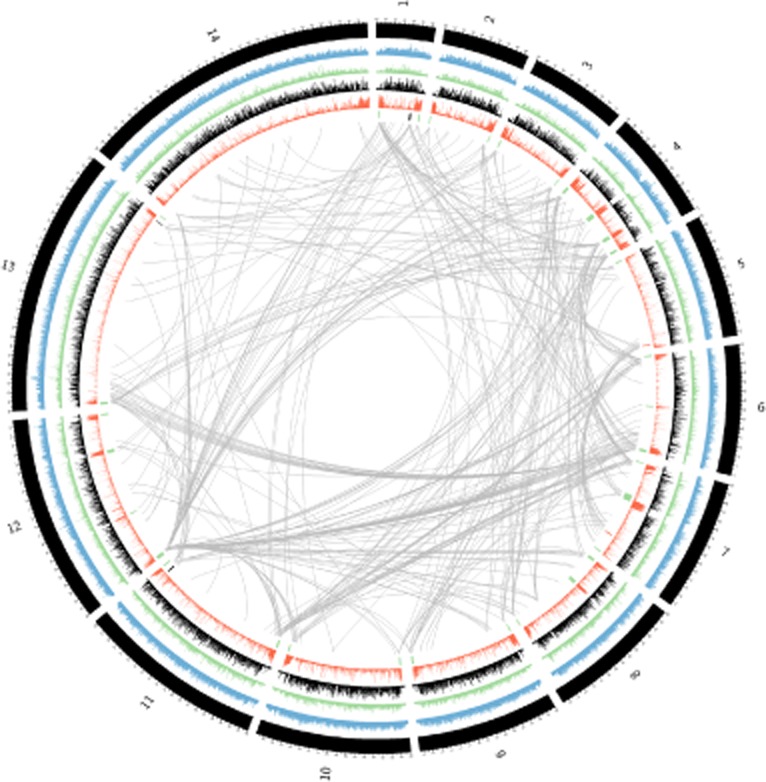
Circos plot illustrating whole-genome interactions with information on histone methylation and acetylation. In a Circos diagram (Krzywinski *et al.*, 2009), the concatenated chromosome co-ordinates are plotted around a circle; one-dimensional data (genetic landmarks and ChIP data) are overlaid on genomic co-ordinates while two-dimensional data can be drawn as links between points on the circle. In blue are H3K4me3 sites from late ring-stage parasites; in green are H3K9ac sites; in black are H2A.Z sites; and in red are H3K9me3 sites [ChIPseq and ChiP-chip data from Bártfai *et al.* (2010) and Mancio-Silva *et al.* (2010)]. The strongest 0.1% of interactions, by read count, are shown with a grey line (this threshold was chosen to avoid overplotting, as displaying, e.g. the top 1% of interactions resulted in a solid grey diagram; a bin resolution of 25 Kb in the ‘DCJ on’ library, with random placement of reads which map degenerately; normalized control libraries have been subtracted from the interaction libraries, as described in Supplemental Note 1). In the inner-most band, abutting the smooth grey curves which denote interactions, important genetic features are displayed. Vertical bars show the genomic location of important elements: The locations of *var* genes are marked by vertical bars in green; A-type rDNA subunits are in red; and S-type rDNA subunits are in black. Intra-chromosomal interactions are suppressed for plotting clarity. Consistent with observed FISH data (Freitas-Junior *et al.*, 2000; 2005,; Duraisingh *et al.*, 2005), chromosome ends and internal variant antigen clusters that are low in activating marks [H3K9ac and H2A.Z (Bártfai *et al.*, 2010)] and high in silencing marks [H3K9me3 (Mancio-Silva *et al.*, 2010)] are major sites of inter-chromosomal interactions in the malaria nucleus.

A large number of the inter-chromosomal interactions that we identified appeared to map to areas of known heterochromatin such as telomeres and internal *var* gene regions. We compared our interaction maps with the genome-wide histone modification maps from the 3D7 parasite (Lopez-Rubio *et al.*, 2009 and Bártfai *et al.*, 2010). After subtracting normalized control libraries from the HiC data, subtelomeric regions and areas of internal *var* gene clusters that have high levels of the silencing mark H3K9me3 and low levels of activating marks [H3K9Ac and H2A.Z (Lopez-Rubio *et al.*, 2009; Bártfai *et al.*, 2010)] had an elevated integrated interaction density, as measured by the sum of all contacts in a given 25 Kb bin (Wilcoxon rank sum test, *P* = 2.2 × 10^−16^; Fig. S7), consistent with their presence in densely packed foci of heterochromatin. These results were in agreement with the results of previous FISH studies (Freitas-Junior *et al.*, 2000; 2005,; Duraisingh *et al.*, 2005; Mancio-Silva *et al.*, 2010) that have established a link between H3K9me3 clusters and heterochromatin foci at the nuclear periphery. We displayed this graphically by combining the interaction data with publicly available genome-wide chromatin immunoprecipitation data sets. The combined data sets are presented in Fig. 5. The top 0.1% interactions in an NF54-derived library are shown as grey curves connecting the interacting regions. Consistent with the quantitative comparisons above, the Circos plots (Krzywinski *et al.*, 2009) demonstrate visually that areas high in activating chromatin marks (H3K9ac and H2A.Z obtained from Bártfai *et al.*, 2010) show fewer inter-chromosomal interactions, whereas areas rich in the silencing mark H3K9me3 showed a propensity to interact more strongly.

Broadly, the maps constructed for ring-stage malaria parasites revealed both similarities and differences between the malaria genome and other eukaryotes. We do not observe the ‘plaid’ patterning of human chromosomes (Lieberman-Aiden *et al.*, 2009) or the existence of preferences in inter-chromosomal interactions typically interpreted as chromosome territories (Lieberman-Aiden *et al.*, 2009; Yaffe and Tanay, 2011). Conversely we observed major similarities between the malaria and human genomes in terms of the mathematical scaling of contact probability as a function of distance and in the role of chromatin in structuring interactions (Lieberman-Aiden *et al.*, 2009; Yaffe and Tanay, 2011). Outside of heterochromatin clusters and rDNA loci, we found relatively few, specific long-range interactions that stood out above the background signal (Figs 2, 4 and 5), suggesting a genome that is structured during the ring stage by the anchoring of heterochromatin foci at the nuclear periphery and rDNA clusters in the nucleolus, but otherwise positioned by non-specific and diffusive forces.

#### Antigenic variation

We next studied changes in this pattern of interaction during *var* gene transcriptional switching. There was a very strong correlation between whole-genome interaction matrices from isolates of identical genetic background but which expressed different *var* genes (Fig. S4F and G; mean pairwise correlation of *r* = 0.99 for IT isolates and *r* = 0.98 for NF54-derived isolates for MboI HiC libraries with 25 Kb bins). This suggests an absence of global reconfiguration after *var* gene switching. Inspection of the whole-genome interaction matrices also failed to identify major structural changes associated with *var* switching (Fig. S4F1–2) that might have been missed by the correlation analysis which considers individual bins as independent. We found a similar pattern when searching for major changes in intra-chromosomal interactions on the chromosomes that contained individual *var* genes (Fig. S3A–E). The mean pairwise Pearson correlation coefficient between the chromosomes in isolates expressing distinct *var* genes was not significantly different for chromosome 13, where the A4 *var* gene resides, compared with the other chromosomes (Fig. S3E; *P* = 0.74, two-sample *t*-test), and the same was true for the NF54 lines. Among the six between-chromosome comparisons where a *var* locus was differentially active, the mean pairwise correlation (*r* = 0.78) was not significantly different from the overall mean (*r* = 0.74) (two-sample *t*-test, *P* = 0.65) at a resolution of 5 Kb bins. Nevertheless, these global measures of similarity have drawbacks (e.g. low power resulting from a small number of comparisons and the questionable assumption of independence between bins) and thus do not rule out specific changes. We therefore conducted a more thorough analysis of the libraries in our study to assess whether there were subtle or locus-specific changes in spatial interactions.

**Fig 6:**
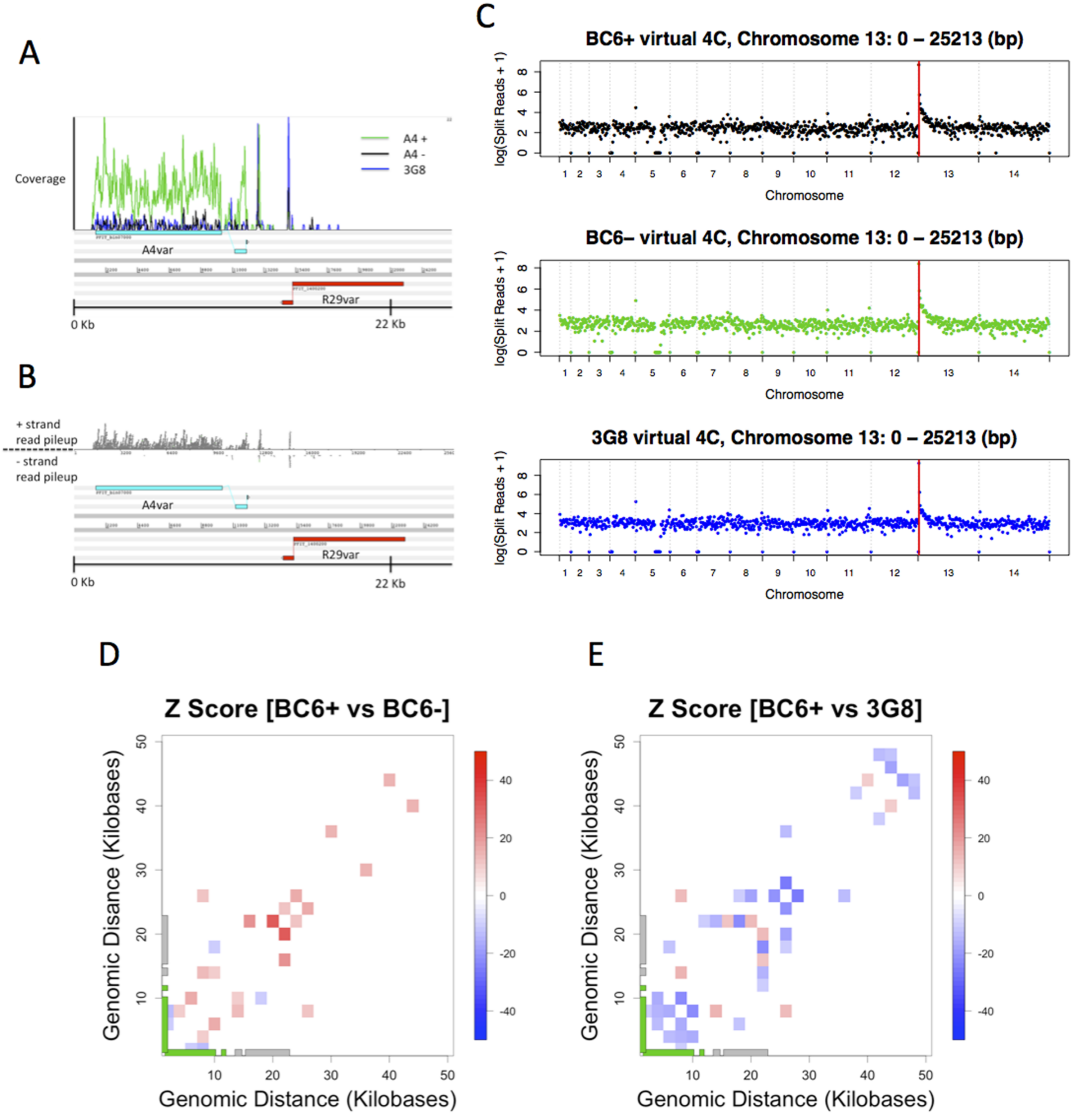
Genome-wide interactions of a 25 Kb bin covering the upstream, coding and downstream of the *A4var* gene.A. Artemis display of RNAseq coverage along the length of the *A4var* gene in the BC6+ (green), BC6− (black) and 3G8 (blue).B. Total coverage from the three libraries in (A) displayed as strand-specific coverage. Note that, in order to generate the IT reference for chromosome 13, the first 28 Kb, which were obtained by sequencing of this subtelomeric region in our previous work (Kyes *et al.*, 2007), were grafted onto the left side of the chromosome following automated assembly. This grafting procedure left some sequence unassembled in the current IT reference.C. Virtual 4C plots of whole-genome interaction matrices for an individual segment, in this case the 25 Kb bin encompassing the *A4var* gene, from the A4 MboI HiC libraries. No differences in global interactions could be observed between the IT subclones which differ at their expression of the *A4var* gene. Each dot represents the interactions between the *A4var* locus, marked by a solid vertical bar, and the corresponding genomic bin of 25 Kb.D and E. Local changes in interaction density at the 25 Kb surrounding the *A4var* locus on chromosome 13 (the same 25 Kb fragment used for virtual 4C analysis above) are shown. The heatmaps display the normalized difference in interaction number between BC6+ and BC6− lines (D) and BC6+ and 3G8 (E); red colouring corresponds to 2 Kb bins with increased interaction frequency in the BC6+ line whereas blue corresponds to bins with decreased interaction in the BC6+ line. Bins with an absolute Z-score of less than 10 are not plotted. The gene models for the underlying sequence are shown. Two *var* genes are present in a tail-to-tail configuration, with the *A4var* marked in green and the adjacent *R29var* in grey. A reproducible pattern of subtle rearrangements is apparent between the two panels. A loop appears to form involving the intergenic region between the active *A4var* gene and the adjacent, inactive *var* gene (*R29var*), and involving the 5′ region of the inactive *R29var* gene.

Contemporaneous RNAseq of parasites from which the corresponding GCC and HiC libraries were derived confirmed that the BC6+ line strongly expressed the *A4var* gene while the BC6− line and the 3G8 subclone did not (Fig. 6A and B and Fig. S1). Stage estimation also confirmed that the input samples were of the same temporal development and synchronicity (Fig. S1D and E). We then sought to identify whether there was specific reorganization at the *A4var* locus by examining virtual 4C data (Fig. 6C). Examining 25 Kb of sequence upstream, overlapping and downstream of the *A4var*, we detected a reproducible genome-wide interaction profile from these loci that looked essentially identical among parasite lines studied despite their distinct expression patterns from this locus (Fig. 6C). This observation suggests the absence of a distant enhancer element responsible for controlling mutually exclusive expression. However, examination of intra-chromosomal interactions in the immediate vicinity of the *A4var* gene revealed modest alterations in interactions (Fig. 6D and E, Fig. S8A). The underlying reference sequence makes further interpretation of these changes difficult since the assembly is incomplete in this area (including the upstream region of the *A4var* gene that is absent from the assembly).

We then repeated these analyses in data obtained from the NF54-derived lines (Fig. S8B–D). While these lines had fewer total reads, and the expression of the *var* gene was artificially controlled in the transfectants, we were able to survey the complete genome because of the quality of the reference sequence. Thus, we would be more likely to detect spatial changes occurring between subtelomeric regions that are absent in the current IT reference. For this analysis, which focused on relative differences between libraries and not absolute interaction in a single library, we used the unfiltered maps with degenerately mapping reads placed probabilistically. This was done because the comparison itself should serve as an internal control (regions with spurious interaction due to homology should generate a signal in both libraries, which would cancel in a relative comparison), and because it gave us maximal ability to study low-complexity and homologous regions. Consistent with the results in the IT clones, we again failed to detect any novel or consistently altered long-range binding of the differentially expressed loci or adjacent regions between the isolates studied that would indicate the presence of enhancer elements.

**Fig 7:**
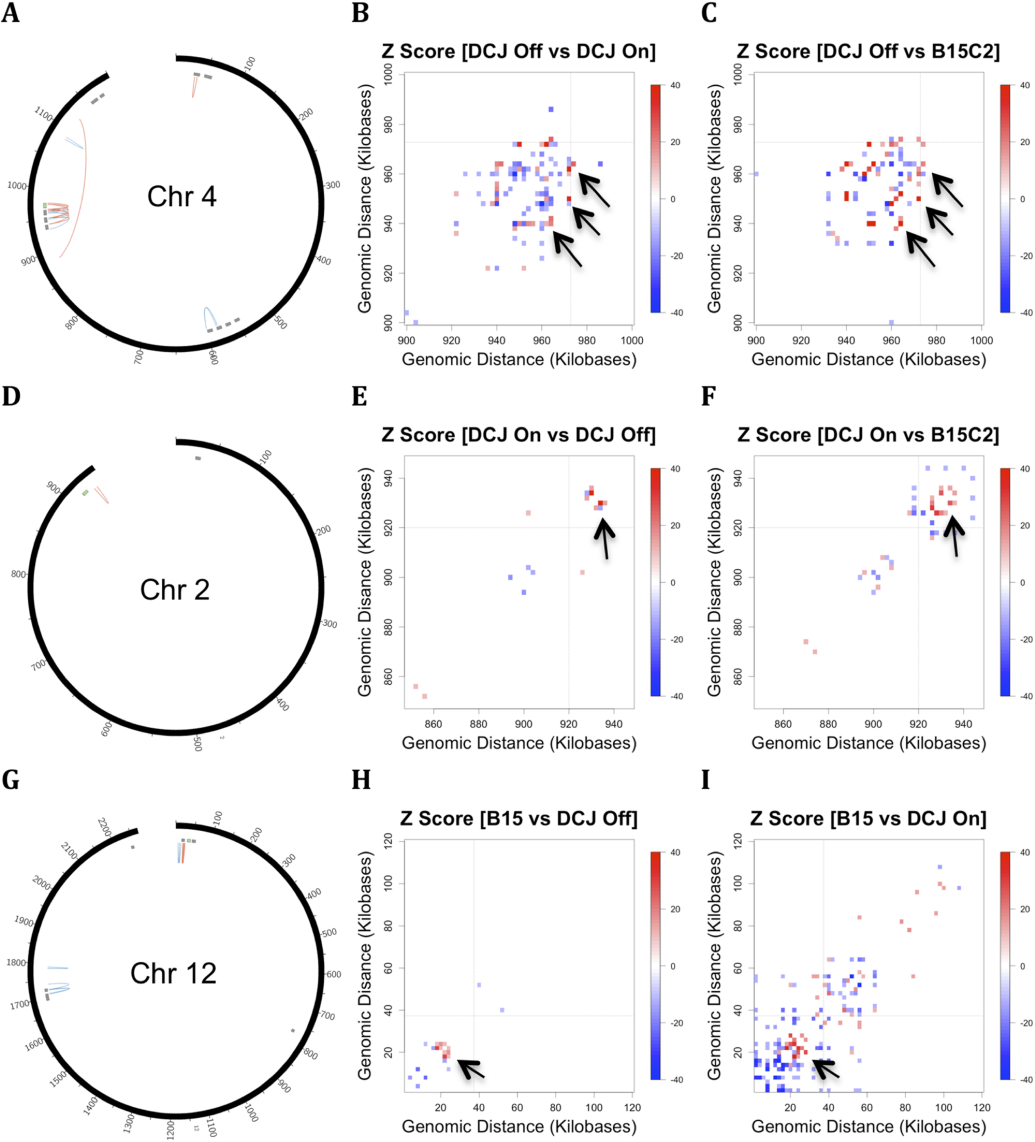
Interaction alterations associated with *var* switching. Local alterations in interaction are associated with the activation of three distinct *var* loci in the MboI HiC libraries for the NF54 isolates. The Circos plots in (A), (D) and (G) show alterations in intra-chromosomal contacts associated with activation of individual *var* genes (coloured in green). The remaining *var* genes on the chromosome are shown in grey. Areas of the chromosome that show an increase in interaction, in both comparisons, are drawn with red links, while areas that decrease in interaction are drawn with blue links. For each row, the local region containing the activated locus is shown for each of the two comparisons (B–C, E–F and H–I, corresponding to the diagrams in A, D and G respectively). In the heatmaps, the interaction matrices for individual libraries were subtracted, and the difference was standardized to generate a Z-score. The scale is in kilobases for both the Circos diagrams and interaction matrices. The Circos plots were filtered to display the strongest interactions (with an absolute Z-score greater than 20 when both comparisons were combined) and which had a consistent direction of change in both comparisons. The adjacent matrices only display bins with a Z-score greater or less than 10. In all cases, while global alterations in chromosome contacts associated with *var* switching were absent, the strongest alterations observed were in the local region of the activated *var* gene.

As above, we next looked for local changes in chromatin interactions at the three *var* loci differentially expressed in the lines (Fig. 7, Fig. S8E–G). In all cases, the most substantial changes occurred in the region adjacent to the activated *var* locus (as shown by the whole-chromosome Circos plots in Fig. 7A, D and G), with the interaction matrices for the local area amplified at right (in Fig. 7B–C, E–F and H–I respectively). The local changes differed depending on the chromosomal location of the activated *var* locus. The internal *var* gene on chromosome 4 that is active in the DCJ off parasite underwent an increase in interaction frequency at the level of the entire cluster in which the active *var* gene resided (marked by arrows in Fig. 7B and C; virtual 4C plots are given in Fig. S8E–G). In contrast, the two subtelomeric *var* genes studied seemed to be relatively unchanged at the activated locus itself, but showed a major interaction alteration slightly upstream (marked by arrows in Fig. 7E–F and H–I). For each of the *var* genes studied, these patterns were reproducible in the GCC libraries (Fig. S8H–O). In the case of the PF3D7_0223500, this altered interaction occurred at the site of a non-coding RNA (Broadbent *et al.*, 2011), while for PF3D7_1200400, the interaction involved the coding region of the adjacent *var* gene PF3D7_1200100 and the region between the two *var* genes, which is annotated to contain two *rifin* genes.

At a genome-wide level, the results we observed with the DCJ/NF54 libraries were consistent with those observed in the IT subclones. We were unable to detect substantially altered long-range interactions in either case, suggesting that mutually exclusive expression of *var* genes in *P. falciparum* does not depend on a mechanism similar to that of an H or P-like element. In contrast, we observed local reconfigurations in spatial structure during gene activation or silencing that are consistent with previous studies showing a role for the local chromatin environment (Chookajorn *et al.*, 2007; Lopez-Rubio *et al.*, 2007; Mancio-Silva *et al.*, 2010) and/or paired-promoter silencing (Deitsch *et al.*, 2001; Calderwood *et al.*, 2003) in the control of *var* gene transcription.

## Discussion

We have studied basic structural properties of the *P. falciparum* nucleus during the ring stage and assessed the relationship between chromosome interaction, chromatin modification and *var* gene activation. Our data provide, to our knowledge, the first global survey of the chromosome interactions in the malaria parasite *P. falciparum*.

We found that polymer physics, chromatin state and the nucleolus are the major structural organizing forces in the genome. The importance of heterochromatin foci in organizing the malaria nucleus is well known (Freitas-Junior *et al.*, 2000; Mancio-Silva *et al.*, 2010) and a recent article drew attention to the role of the nucleolus in constraining the nuclear location of telomeres (Mancio-Silva *et al.*, 2010). We additionally show substructure in the nucleolus that involves clustering of the A-type rDNA loci that are actively transcribed during asexual stages, and also show that the remainder of the genome remains comparatively unstructured. The driving force behind nucleolar clustering of rDNA loci is not currently known but the clustering of inactive loci with active loci suggests that this physical association does not depend on active transcription alone.

Our data suggest that any repositioning that occurs during *var* switching is predominantly local in character. We noted changes in local chromatin interaction during *var* gene switching that appeared much more pronounced when a gene in a central cluster was involved. Although this may be a result of limited statistical power in telomere regions, these changes in local DNA interactions are consistent with the apparent requirement for paired promoters for *var* gene silencing (Deitsch *et al.*, 2001).

Mutually exclusive expression of *var* genes drives the process of antigenic variation in the malaria parasite *P. falciparum*. Some progress in understanding mechanisms governing *var* gene expression has been made (Kyes *et al.*, 2001; Scherf *et al.*, 2008), but many questions remain unanswered. Active *var* genes occupy a transcriptionally permissive zone, while other, silenced members of the gene family reside in a compacted state in heterochromatin clusters at the nuclear periphery. Their silencing is maintained by their subnuclear localization, chromatin condensation mediated by histone modifications such as H3K9me3 and histone modifying enzymes such as PfSIR2 and likely other molecular factors (Deitsch *et al.*, 2001; Calderwood *et al.*, 2003). DNA elements, involved in the pairing of upstream and intron *var* promoters that are essential for *var* gene silencing, have also been described (Avraham *et al.*, 2012). Other elements of the expression control system nevertheless remain puzzling. How does the cell ‘count’ the number of expressed *var* genes? How do transitions occur between *var* genes? What factors can account for the observed associations between switch rate and chromosomal position?

We tested the hypothesis that mutually exclusive expression is maintained by a long-range association with a chromosome element. The theoretical rationale for this hypothesis is clear. First, an enhancer element present at one copy per genome is an attractive mechanism to drive expression of the active *var* because a single pan-enhancer with different affinity for distinct regions of the genome could explain both mutually exclusive expression (Scherf *et al.*, 1998) and unequal switch rates (Recker *et al.*, 2011). Second, because each chromosome element is present in exactly one copy prior to DNA replication, an enhancer-driven activation mechanism circumvents some of the inherent ‘leakiness’ in promoters that are sensitive to unavoidable stochastic variation in the levels of other expressed, soluble macromolecules such as RNA and proteins (Elowitz *et al.*, 2002; Lestas *et al.*, 2010). Such a mechanism would resemble the role of the H- and P-elements in the mammalian olfactory receptor system (Lomvardas *et al.*, 2006; Fuss *et al.*, 2007).

Despite the attractive theoretical reasons for the presence of such an enhancer, we were unable to identify such an element in the *P. falciparum* genome. Given that the inter-chromosomal interactions mediated by the mouse H-element were identified using chromosome conformation capture techniques, our positive identification of known rDNA interactions and heterochromatin clustering and the consistency of our results across parasite lines, it seems likely that mutually exclusive expression in *P. falciparum* is not achieved by a trans-acting enhancer functioning during ring stages. From the experiments presented here, we cannot rule out the presence of a H- or P-type element that affects only a subset of *var* genes, or acts only during trophozoite and schizont stages. Nevertheless, recent studies support the striking similarities between the OR genes and *var* genes. Since the initial characterization of H-element inter-chromosomal interactions (Lomvardas *et al.*, 2006), it has been shown that the H- and P-elements play a more restricted role in OR gene choice, acting predominantly in an intra-chromosomal capacity (Fuss *et al.*, 2007; Bozza *et al.*, 2009; Khan *et al.*, 2011). In both systems, a combination of reversible heterochromatin formation, distinct subnuclear positioning (Duraisingh *et al.*, 2005; Magklara *et al.*, 2011; Clowney *et al.*, 2012) and a hierarchy of local regulatory elements may control both mutually exclusive expression and the likelihood of gene activation.

There is a trade-off in GCC-based methods between sensitivity to detect subtle rearrangements at a single locus and the ability to profile the entire genome as a whole. Locus-specific techniques such as 4C may be able to detect more subtle chromatin structural rearrangements. While many of the *var*-specific changes occurred at a level of chromosomal detail inaccessible with standard fluorescence microscopy, it is possible that the use of super-resolution techniques could help characterize these reconfigurations in individual cells. One important question is whether this reconfiguration drives other epigenetic modifications that occur at the active *var* locus, or whether the reverse occurs, i.e. the altered local profile of spatial contacts simply results from diffusive or non-specific forces acting on a portion of chromatin fibre with altered stiffness or flexibility, secondary to changes in histone marks. In our view, the lack of an archetypal spatial interaction pattern for active *var* loci would seem to support this latter hypothesis.

Understanding the spatial organization of the malaria nucleus provides a unifying framework for the growing quantity of epigenomic and transcriptomic data for the *P. falciparum* parasite under various conditions. A consensus view of the parasite nucleus is emerging in which the expression of the vast majority of genes is controlled predominantly at the level of stage- and lineage-specific expression patterns conserved among parasite strains (Bozdech *et al.*, 2003; Le Roch *et al.*, 2003; Llinás *et al.*, 2006; Ganesan *et al.*, 2008). In contrast, a subset of the genome shows greater inter-clone variation (in genetically distinct and isogenic parasites), and the genes in this subset include the *var* genes, other genes encoding surface-associated proteins and genes that are present in heterochromatin clusters at subtelomeres and internal *var* gene clusters (Scherf *et al.*, 2008; Mancio-Silva *et al.*, 2010; Rovira-Graells *et al.*, 2012). In this paradigm, expression switching is associated with reconfigurations in the chromatin state of promoters rather than signalling cascades that respond to the extracellular environment, as is the case in most eukaryotic cells. Chromatin state in turn is closely associated with spatial position and interaction probability. The genome appears to be partitioned into three compartments, one at the nuclear periphery which contains heterochromatin, one at the centre of the nucleus which contains euchromatin (Duraisingh *et al.*, 2005; Freitas-Junior *et al.*, 2005; Lopez-Rubio *et al.*, 2009) and a third in the peri-nuclear nucleolus (Mancio-Silva *et al.*, 2010). Our data are consistent with the models of the *P. falciparum* nucleus previously described [e.g. fig. 3F of Lopez-Rubio *et al.*, (2009) and fig. 3E of Mancio-Silva *et al.* (2010)], but add additional detail by showing that these mechanisms that partition the genome between heterochromatin and euchromatin and the clustering of rDNA elements in the nucleolus are the dominant mechanisms that are structuring the *P. falciparum* nucleus during the ring stage when observed globally in an unbiased fashion. The convergence of intra-chromosomal contact probability at distances above 1.5 Mb with that of inter-chromosomal contact probability suggests an absence of chromosome territories, and the distribution of inter-chromosomal contacts among chromosomes is consistent with a model of non-preferential association between chromosomes. In this respect, the malaria genome is comparatively unstructured when compared with larger genomes.

It will be important to develop a more complete understanding of the technical subtleties of global chromosome conformation capture data. For example, early in our analysis we found that amplification and deletion events generated artificial signals of interaction density in the genome (Supplemental Note 3 and Fig. S9). High-throughput technologies, including second-generation sequencing and microarrays, have been shown to have significant bias resulting from the GC content of the underlying sequence, the presence of non-unique sequence and the preparation procedures used to generate libraries. It is likely that both digestion and proximity ligation as well as the downstream sequencing introduce bias into the interaction maps and understanding this bias, quantifying it and correcting for it in future efforts will improve the overall quality of chromosome interaction data sets. Furthermore, both longer read lengths and improved analytical methods should help resolve homologous regions of the malaria genome. Our approach of either analysing uniquely mapping reads, removing bins from the maps which generated a signal in the control libraries, or subtracting the interactions observed in the control library may have failed to capture some interesting spatial features of these difficult-to-resolve portions of the genome. However, the consistency of results across different mapping approaches suggests that the read length used in this study (76 bp) was adequate to resolve structural features of the genome outside the highly polymorphic regions such as telomeres.

It is tempting, from our own data and that of others, to imagine a picture of the malaria nucleus with clusters of telomere ends bound together at several distinct heterochromatin foci at the nuclear periphery, with rDNA clusters and their associated chromosome ends anchored in a single, peri-nuclear nucleolus and with the euchromatin of the remainder of the genome relatively free to diffuse. However, the dynamics of the formation and breakdown of structures and to what extent individual cells adopt structures resembling the ensemble average, remain important questions for future investigation. The information obtained from a chromosome conformation capture experiments is a single snapshot in time, averaged over a large population of cells. Furthermore the relationship between contact probability (measured by 3C-based techniques) and spatial distance needs to be understood. Under some simple models of polymer folding, such as the Gaussian chain, these are related analytically. However a general theoretical model for polymer folding in the nucleus is still unavailable and at present the relationship between these two observable parameters remains unclear. A better understanding of the individual configurations that make up the population-ensemble of nuclear structures is needed. Such an understanding is likely to be obtained through a combination of theoretical modelling, careful experimental combination of FISH and 3C-based techniques, and an extension of proximity-ligation methods to single-cell resolution.

In summary, we have constructed high-resolution maps of the ring-stage *P. falciparum* nucleus and used these maps to study the reconfigurations in chromosome interaction that occur during switches in *var* gene expression. We provide evidence for the absence of a pan-enhancer underlying the mutually exclusive expression mechanism of *var* genes and suggest that the majority of interaction changes coincident with *var* switching are local in nature. More generally, our experiments offer a global view of the parasite nucleus across multiple scales and underscore the importance of heterochromatin foci, rDNA interactions and the polymeric nature of chromosomes as the dominant organizing forces of the parasite nucleus.

## Experimental procedures

### Cultivation of *P. falciparum* parasites

Parasites were cultured according to the method of Trager and Jensen (1976). Cultures were synchronized by Plasmagel flotation (to obtain enrichment of trophozoite stages) or sorbitol lysis (to obtain enrichment of ring-stage parasites) (Lambros and Vanderberg, 1979). Where necessary, highly synchronized cultures were achieved using three rounds of sorbitol synchronization and Plasmagel flotation. Synchronicity was verified by microscopy of Giemsa stained blood smears. Strain DCJ was grown without drug to generate strain DCJ off, and grown with 4.7 μM blasticidin S pressure for 1 month to generate strain DCJ on. Strain B15C2 was grown under continuous blasticidin S pressure for 1 month.

### Antibody-based selection of PfEMP-1-specific clones

The IT isolate and its subclones were used for phenotypic selection experiments. The A4 subclone was positively and negatively selected for expression of the BC6 epitope. Parasites were positively selected for expression of the A4var-encoded PfEMP1 by incubating 20 μl of 400 μg ml^−1^ BC6 antibody with 50 μl of Protein G-coated magnetic beads (Invitrogen) in 150 μl of 1× PBS/1% BSA in a sterile tube at 25°C for 2 h. The beads were washed once with 1× PBS/1% BSA and incubated with Plasmagel purified trophozoites at 25°C for 30 min. The beads containing bound parasites were washed twice with 1× PBS/1% BSA, resuspended in a small volume of culture medium, and returned to parasite culture with fresh medium and cells. Enrichment of parasite populations for BC6-negative parasites was performed by removal of BC6-positive parasites as follows. BC6 antibody was bound to beads as above. The number of parasitized trophozoites was calculated to be 1/10 the number of magnetic beads. Incubation of beads and parasitized RBCs together was performed for 2 h at 25°C. Magnetic beads were gently pooled at the side of the tube and the supernatant was collected and returned to culture with fresh uninfected red cells and fresh medium.

### Chromosome conformation capture (GCC and HiC)

GCC was performed as in reference (Dekker *et al.*, 2002) with modifications for malaria as described below. Cultures were harvested at 5% parasitaemia. A ring-stage culture of 1 ml packed red cells at 5% parasitaemia typically provided enough material for six to eight 3C or HiC reactions. Parasite pellets were cross-linked with 1% paraformaldehyde (Electron Microscopy Sciences) for 10 min at 25°C. Cross-linking reactions were quenched with the addition of glycine to a final concentration of 125 mM and incubated for 10 min at 25°C. Parasites were isolated from infected red cells by incubation in 0.1% saponin/1× PBS. Incubation with 0.1% saponin was repeated three times in and the pellet was recovered by centrifugation at 14 000 *g*. The pellet was then washed twice in 1× PBS followed by centrifugation at 14 000 *g*. Nuclei were prepared by incubation of the saponin-lysed pellet in 10 mM Tris-HCl, 2.5 mM MgCl2, 14 mM 2-mercaptoethanol 0.5% Nonidet P-40, pH 7.5, and protease inhibitors (complete EDTA free protease inhibitors, Roche) at 4°C for 10 min. Nuclei were collected via centrifugation in a microcentrifuge at 4°C for 7 min at 2500 *g*. A 75 μl packed pellet of nuclei was used in a single 3C or HiC reaction. Nuclei were then resuspended in digestion buffer (NEBuffer 2 (50 mM NaCl, 10 mM Tris-HCl, 10 mM MgCl_2_, 1 mM DTT, pH 7.9). SDS was added to a final concentration of 0.3% and the nuclei were incubated for 30 min to 1 h at 37°C with shaking at 900 r.p.m. Triton X-100 was then added to a final concentration of 2% and shaking continued for a further 30 min to 1 h. Nuclear clumping was sometimes observed at this stage and was reduced by pipetting up and down to mix the samples. Restriction enzyme was then added (500 units HindIII, or 400 units MboI added over two to three time points) and cells were digested at least 16 h.

The following day, restriction enzymes were inactivated by addition of SDS to 1.6% final concentration, and incubation at 65°C for 25 min. Each 500 μl reaction was diluted into 8 ml of ligation buffer containing 1% Triton X-100. T4 DNA ligase was added in the amount of 10 units per tube (Invitrogen) and reactions were incubated at 16°C for 4 h, followed by a further 30 min at room temperature. After ligation, 500 μg of proteinase K (Sigma) was added and tubes were incubated at 65°C overnight. The following morning a further 500 μg of proteinase K was added and incubated for 2 h at 50°C. DNA was purified using phenol-chloroform extraction and precipitated using 0.1 volumes of sodium acetate and 2.5 volumes of 100% ethanol.

HiC was performed as GCC with the following modifications. After > 16 h of digestion with a restriction enzyme, but prior to inactivating that enzyme, 1.5 μl of 10 mM dATP, dGTP, dTTP was added to each reaction, along with 37.5 μl of biotin-14-dCTP (Invitrogen). Ten units of Klenow (NEB) were then added and the samples incubated at room temperature for 45 min. During the ligation step, 40–50 units of T4 DNA ligase (Invitrogen) were used. Streptavidin purification was performed as described in reference (Lieberman-Aiden *et al.*, 2009), and sequencing was as described below.

### RNA extraction, Northern blotting and qRT-PCR

RNA extraction and Northern blotting were performed as previously described (Kyes *et al.*, 2000). Complementary DNA synthesis was performed using the Superscript III kit according to manufacturer instructions (Invitrogen). Quantitative real-time polymerase chain reaction (qRT-PCR or qPCR) was performed on a RotorGene 6000 with SYBR mix (Quantace) diluted to a final concentration of 1×, along with 100 ng cDNA or template DNA at 100 ng or less with a 10 μl reaction volume. Cycling conditions were as follows: 95°C for 20 s, 58°C for 30 s, 68°C for 30 s, for 40 cycles. The primers used to measure 3D7 *var* gene expression were those from Salanti *et al.*, (2003) with modifications by Dzikowski *et al.* (2006) for the DCJ lines. Typical reaction volume was 10 μl. Estimates of concentration were obtained by the ΔΔCt method.

### On-flowcell reverse transcription sequencing

FRTseq was performed as described (Mamanova *et al.*, 2010). Briefly, RNA was extracted as described above from a portion of the culture immediately prior to cross-linking of the remainder for chromosome conformation capture analysis. Adapters were attached and reverse transcription was performed on the flowcell, allowing for strand-specific and amplification free analysis of transcript quantity. The data have been deposited in ArrayExpress under Accession No. E-ERAD-75.

### Flow cytometry

Primary antibodies were pre-cleared on uninfected red cells by incubating the total antibody used in the experiment with 100 μl of uninfected red cells for 30 min at 25°C. Secondary antibodies were pre-cleared with 100 μl of infected red cells for 30 min 25°C.

Flow cytometry was performed in triplicate on 10 μl of cells. Cells were washed twice with 1× PBS/1% BSA and incubated with primary antibody, suspended in 100 μl 1× PBS/1% BSA, for 30 min at 37°C. The BC6 antibody was used at a concentration of 5 μl of 400 μg ml^−1^ per well. Cells were then washed twice with 1× PBS/1% BSA and incubated for 30 min at 37°C with secondary antibody (goat anti-rabbit conjugated to APC, Invitrogen) resuspended in 1× PBS/1% BSA along with SYBR green stain diluted to 0.1×. After washing twice with 1× PBS/1% BSA, cells were fixed with 1% paraformaldehyde/1× PBS/1% BSA and analysed on a flow cytometer.

### Second-generation sequencing

To sequence the samples, they were first fragmented to libraries of 200–400 bp and prepared following the Kapa HiFi protocol (Quail *et al.*, 2011; Oyola *et al.*, 2012) and then marked with a molecular barcode. Before sequencing on an Illumina GAII or HiSeq the libraries were denatured and hybridized to the flowcell. Sequencing was performed with paired end reads with a read length of 76 bases. We deposited all sequences at the short read archive at http://www.ebi.ac.uk/ena/with Accession No. ERP001407.

### IT genome assembly and improvement

The current *P. falciparum* IT assembly version 2 (http://www.genedb.org) (Logan-Klumpler *et al.*, 2011) was additionally corrected by moving the *A4var* cluster to the left-hand side of chromosome 13 (Kyes *et al.*, 2007). The *var* gene ITvar7 which was at that position was moved to the unordered contigs bin. This modified genome version was called v2.5.

### Computational analysis

Reads were mapped to the *P. falciparum* genome using Smalt v. 0.5.8 using the –x flag (which triggers an exhaustive search for each read and chooses the optimal alignment independently). The –*r* flag was used for when reads which mapped to multiple sites in the genome were included in the analysis. BAM files were filtered for read-pairs which mapped greater than 2000 bases apart on the same chromosome or to different chromosomes. Lists of read co-ordinates were read into the statistical software environment R (Ihaka and Gentleman, 1996) (v. 2.14) and used to generate interaction matrices, compute statistical summaries and fit models. For the FRTseq libraries, expression values were calculated from BAM files using Cufflinks (Trapnell *et al.*, 2010) or Artemis (Rutherford *et al.*, 2000). All logarithms used in the article are to base e except where otherwise noted.

### Significance testing procedures

We propose a procedure to estimate the significance of inter-chromosomal interactions that is designed to overcome two potential difficulties with this type of data. First, we cannot directly measure the null distribution of non-specific inter-chromosomal interactions. Instead, we observe a mixture of the null distribution and some likely small number of biologically relevant interactions. Second, when we do observe genuine interactions, we expect such signals to exhibit spatial autocorrelation due to the polymeric nature of chromosomes. In other words, when an interaction occurs between two chromosomes, we expect chromosomal segments nearby to interact more often.

To account for these properties of the data, we propose a Leave-One-Out (LOO) or jackknife-type estimation procedure (Hastie *et al.*, 2001). First, we approximated the null distribution of inter-chromosomal interactions based on robust test statistics of the distribution excluding the point of interest. Specifically, for each bin of interest, i, we calculate the median and interquartile range for the remaining n − 1 bins and use these statistics to approximate a Normal distribution. Importantly, these robust estimators will not be strongly influenced by the comparatively small number of interactions that represent specific interactions. Second, to account for dependence between bins, we removed the adjacent 150 Kb in constructing the estimate of the null distribution, such that the removal of bin i described above actually represents a range of i ± k bins (with k = 3 for 25 Kb bins), reflecting the expected spatial autocorrelation of genuine interaction signals. Intra-chromosomal interactions were also removed in this analysis, since the null distribution for non-specific contacts is distant-dependent and therefore is not expected to follow a null distribution of the same form as inter-chromosomal interactions.

To test for significance in an interaction data set, we performed this procedure for a row of an interaction matrix, and for each bin estimated the null distribution from the interaction data for the remaining bins in the row. *P*-values were calculated using the Normal approximation described above, and were subsequently corrected for multiple tests using the Benjamini–Hochberg correction (Benjamini and Hochberg, 1995).

### Data access

Data are available at http://www.ebi.ac.uk/ena/ Accession No. ERP001407 and at ArrayExpress under Accession No. E-ERAD-75.
