# Supplementary material for: Genome-wide profiling of chromosome interactions in Plasmodium falciparum characterizes nuclear architecture and reconfigurations associated with antigenic variation
Source: Mol Microbiol. 2013 Sep 30;90(3):519–37. doi: 10.1111/mmi.12381 (PMC3894959; doi:10.1111/mmi.12381)
Supplement: Supplementary file 1 — Supporting Information [file mmi0090-0519-SD1.pdf]

Supplemental Note 1

In order to generate interaction matrices from the chromosome conformation capture libraries, short reads were mapped to the genome using SMALT, a short read aligner from the Wellcome Trust Sanger Institute that permits an exhaustive search of each member of the read-pair (when the –x flag is used). Reads for which individual members of a mate pair mapped greater than 2Kb from one another were considered as 'split read-pairs'. The first base of each aligned read was then used in downstream analysis.

We use two methods of global chromosome conformation capture in this study: GCC and HiC. The GCC protocol (Rodley et al., 2009) directly sequences the 3C library via restriction digestion and sticky-end ligation (Dekker et al., 2002). The HiC protocol (Lieberman-Aiden et al., 2009) expands on the GCC protocol by incorporating a biotinylated nucleotide into the ligation site using the Klenow fragment, which yields blunt ends prior to the ligation step. The main advantage of HiC over GCC is a higher relative proportion of read-pairs per restriction site, since biotin can be purified using streptavidin-coated beads. The main drawback is a decrease in ligation efficiency of the blunt-end ligation reaction. Furthermore, it is not known whether incorporation of the biotin, blunt-end ligation, or subsequent processing and enrichment steps will generate additional artifacts compared to direct sequencing of the 3C library as in the GCC protocol.

The small size of the malaria genome enabled us to compare GCC and HiC. We found that both techniques yielded largely equivalent results. Specifically, the interaction matrices (Figure S4A and S4B) as well as the relationship between contact probability and genomic distance (Figure S5) were qualitatively and quantitatively similar across the two methods. At the

level of individual bins, there was a strong, linear association between read counts in MboI HiC libraries and the MboI GCC libraries (r = 0.97, Figure S4C, panel 4); a similar relationship was observed for the HindIII libraries (r = 0.88, Figure S4D, panel 4). There were some differences between the methods, however. The HiC libraries generated a larger fraction of split read-pairs, which resulted in increased signal. As shown in S4C, panel 6, between 50Kb and 300Kb, the portion of the interaction maps where reads most clearly represented the genuine signal from polymeric contacts, there were approximately 8.5 times as many reads in the HiC libraries as compared to the GCC libraries. This improved signal can also been seen in the scaling relationships between contact probability as a function of distance (Figure S5). While both library types showed linear scaling in the initial part of the curve, this relationship extended for ~500 Kb in the HiC libraries whereas it stopped after ~300 Kb in GCC libraries, at which point in each library type the scaling curve became flat, reflecting non-specific contacts as seen in the control libraries. A second difference, comparably minor, is that the GCC and HiC libraries displayed different slopes for bins with the highest numbers of reads (as shown in the cloud of points in the upper right of Figure S4D panel 4). Points in this region correspond to bins on the diagonal (i.e. a_{ii}), which is demonstrated by generating scatterplots from 'hollow' versions of the interaction matrices (diagonal bins removed) in Figure S4C panel 5. This probably reflects a greater propensity to capture short-distance or self-ligation products in the GCC libraries.

We also examined the effect of using different restriction enzymes on GCC and HiC by constructing interaction libraries using HindIII, an enzyme with a six-base recognition sequence, and MboI, which recognizes a four-base sequence. As anticipated, at equivalent bin resolution, matrices produced using a four-cutter showed reproducible features and improved spatial resolution with lower levels of noise. Matrices produced using MboI began to exhibit high levels

of noise below approximately 10-25 Kb resolution, whereas matrices produced using HindIII exhibited noise below approximately 50Kb, although the exact value of resolution is somewhat arbitrary and was chosen based on the empirical bin size at which the interaction matrices lost obvious holes and irregularities from bins which contained no restriction sites. The resolution of 25Kb for MboI libraries and 50Kb for HindIII was made principally on the grounds that, at these levels, nearly all bins had sufficient numbers of restriction sites to give the matrices a smoothness in accordance with the expected behavior of polymer contacts, which should decay continuously and monotonically with distance. However, a less heuristic method for choosing the bin size would be a desirable topic for future research. In comparisons of the number of reads per bin between conditions, e.g. in Figure 6 and Figure 7, a higher resolution (i.e. smaller bins) could be used since the comparisons between bins do not rely on the values in neighboring bin, i.e. the 'noise' which produces deviations from smoothness in the contact matrices is controlled for in the direct comparisons. The theoretical resolution is determined by the frequency of cut sites as well as the total number of split read-pairs. In general, this was reflected in our analysis. Libraries generated with an enzyme that cuts more frequently had a higher resolution, and libraries with larger number of total reads (as a result of improvements in sequencing capacity over the course of this study, the IT libraries contained larger number of split read-pairs) also resulted in improved signal-to-noise ratios (Supplemental Table 1). In general, in the pilot libraries we used to evaluate the technique initially and in the A4 libraries, the percentage of read pairs that were more than 2Kb apart or on different chromosomes was roughly 40-50% for the MboI HiC libraries, 25-30% of the HindIII HiC libraries, 12-18% for the MboI GCC libraries, and 3-6% for the HindIII GCC libraries (Supplemental Table 1). For unclear reasons, the NF54 libraries followed a less consistent pattern, with most libraries showing roughly 10% of read

pairs more than 2Kb apart or on mapping to different chromosomes, and one set of the GCC MboI with higher ligation frequencies (38 – 48%). This variation in ligation efficiency between libraries did not affect any of the biophysical or biological results drawn from the samples, with GCC and HiC libraries leading to nearly identical scaling of contact probability and interaction signals (e.g. Figure 3b and Figure S4, or Figure 7B-C,E-F,H-I and Figure S8H-L).

The malaria genome contains significant stretches of homology (particularly in the sub-telomeres), which can generate artifacts in the interaction maps. We sought to understand the effect of sequence similarity by comparing two different mapping approaches. The first approach discards reads that did not map uniquely (Figure S4G panel 1). This approach strongly reduces the potential for sequence homology to generate false positive interactions, but it severely restricts statistical power to detect interactions in these regions. Rather than discard these non-uniquely mapping reads, the second approach places such reads at random among sites with matching sequences. While this approach increases the sensitivity to detect positive interactions in these regions (since there is still some information about their location), it also increases the likelihood of false positives. In practice, these two approaches generated similar mappings (c.f. Figures S4E panel 1 and S4G panel 1).

We estimated the propensity of regions to show spurious interactions by generating control GCC libraries with genomic DNA that was digested, ligated, sequenced, and mapped using the same analysis pipeline as the interaction libraries. As expected, and discussed in the main text, these control libraries did not show the characteristic scaling as a function of genomic distance, a result which confirms that the chromosome conformation capture technique effectively detects genuine features of chromosome folding.

The control libraries (Figure S4E panel 2), however, did show a signal in homologous regions of the chromosomes (subtelomeric regions and, to a lesser extent, internal antigenic repeat regions). We attribute this signal to mapping among read-pairs that can align to multiple sites, and some level of interaction along the diagonal, likely due to circularization of large restriction fragments ("self-ligation") which are greater than 2Kb (and therefore not filtered in the analysis pipeline) due to a large distance between restriction sequences or a small number of missed cleavage sites.

Fully correcting for all of these effects requires an extensive statistical model that is beyond the scope of this paper. In addition to correcting for homology, such a model would also account for the multiple sources of correlation across reads as well as overdispersion (such models are a subject of ongoing research, see, for example (Yaffe and Tanay, 2011). Instead, we consider two simple correction methods. First, we remove all elements from the interaction matrix that were scored in the top 1% of read counts in the digested/ ligated gDNA control library (Figure S4E panel 3), resulting in the 'filtered' interaction matrix of Figure 2B and Figure S4 panel 4. This approach broadly corresponds to a model in which significant homology completely confounds the detection of interactions. However, this likely overstates the effect of homology and also fails to take spatial autocorrelation into account.

Second, we normalize the libraries, to generate the same number of counts in the interaction and control libraries, and then subtract the normalized control counts from the observed interactions (S4E panel 5), resulting in the 'corrected' maps. In this case, normalization was applied to the number of total split read-pairs mapping a distance of > 0.5Mb apart, an attempt to remove the effect of background polymer contacts (which affect only the interaction libraries and not the control). This approach broadly corresponds to a model in which

the bin counts in the control libraries follow independent Poisson distributions, such that subtraction yields the residual from the null model. However, this approach relies on the assumption that the normalization is indeed valid, which requires significant validation. In addition, this does not correctly capture effects such as the non-independence between bins due to physical connectedness of the polymer (spatial autocorrelation) or technical assumptions of the Poisson model such as overdispersion. These approaches reflect an effort to control for the effect of homology while recognizing that more research is needed into the nature of noise and bias in genome-wide chromosome conformation capture experiments before a rigorous correction can be applied.

Finally, we considered the reproducibility of the data. We compared all of the interaction matrices for samples generated from IT lines and independently, all of the data generated from NF54 lines. At a resolution of 25Kb, the correlation coefficient for the vectorized, whole-genome interaction matrices from the MboI HiC libraries was $r = 0.99$ between the two A4 libraries, and $r = 0.98$ on average for the libraries from NF54-derived clones. The highly reproducible nature of the data allowed us to pool datasets within a single genotype for some subsequent analyses and visual display. While the conclusions of our analyses were not sensitive to whether libraries of given genotype were pooled, the signal-to-noise ratio in the interaction matrices improved with pooling, which facilitated visual display.

The quality of the reference genome impacts the completeness of the maps produced. In the maps constructed using *P. falciparum* IT (e.g. Figure 2A), regions that appear to lack interaction (such as the region spanning 0.8 Mb – 1.2 Mb on chromosome 5) are due to degenerate, unmappable regions of the genome (in the case of chromosome 5, an MDR1 region

amplification event – see below, supplemental note 3) or areas for which assembly in the current IT reference is imperfect.

The maps constructed for the NF54 and IT genomes differ in that the numbers of contacts between telomeres and subtelomeric regions are somewhat increased in the NF54-derived lines in comparison to those of the IT lineage. This is likely due to the higher quality of the 3D7 reference genome. For the interaction matrices in Figure 2B produced from the NF54-derived strains, we took the conservative approach of removing regions that generated a positive signal in control libraries ('filtered' maps). This prevents potentially false interaction signals generated by homology, but also likely attenuates or conceals a number of genuine interactions. In the parts of the genome removed by the analysis (those bins plotted in Figure S4E panel 3), longer reads or more sophisticated analytical approach may prove helpful in the future.

Supplemental Note 2

Many of the theoretical properties of polymers are understood both quantitatively and qualitatively. Such results are useful in interpreting the data from chromosome conformation capture experiments, for example by verifying that the measured quantitative relationships are consistent with their expected forms, and by comparing evidence for different models of chromosome folding.

Polymers belong to a class of objects known as fractals. Fractals are objects that remain similar when viewed from multiple length scales. These objects are frequently described by power laws of the form $y \sim x^b$ (Mandelbrot, 1983) because this specific mathematical relationship embodies the property of scale invariance (i.e. scaling the function by $f(ax)$ results in $a^{b*}f(x)$, or multiplication of the original function by a factor a^b). Physical quantities described by such relationships are numerous and, relevant here, include the probability of a polymer making a contact with itself as a function of distance (Colby and Rubinstein, 2003), which is also the quantity that is directly observed in a chromosome conformation capture experiment. The reason for this functional form is that a polymer in an ideal solvent can be modeled as a random walk in three dimensions (see, for example, (Flory, 1953)). Based on this model, the power law relationship follows directly from a mathematical analysis of random walk statistics, with a scaling exponent of -3/2 for a walk in three dimensions (Norris, 1998).

We calculated the probability of a chromosome making a contact with itself at a given genomic distance. We estimated the parameter, b, known as the scaling exponent, by calculating the slope of the fit line on a plot whose axes are both logarithmic (Figure 2 and Figure S5). We display the number of split read-pairs because that is the quantity directly observed in the

experiment. The number of split read-pairs we sequenced at a given genomic distance divided by the total number of split read-pairs gives an estimate of contact probability (these numbers are related by multiplication, and the estimate of the slope on log-log axes will be therefore be the same regardless of whether contact probability or total contact number is used). The fit was performed on the initial part of the log(contacts) vs. log(genomic distance) curve, as the relationship did not seem to hold at large distances, reflecting either a genuine physical property of the polymer or an inability to distinguish signal from noise as the signal weakens with distance (Figure S5).

As described in the main text, we observed estimates of approximately -1 for the scaling exponent for contact probability as a function of distance. This numerical value has been observed previously in HiC experiments of human cells (Lieberman-Aiden et al., 2009), a result which is inconsistent with a random-walk polymer at equilibrium. The authors noted that the observed scaling exponent of -1 was consistent with an alternate model of chromosome structure, proposed on theoretical grounds, and known as the ‘crumpled globule’ (Grosberg et al., 1988, 1993). The values we observe of approximately -1 are consistent with this model of genome organization, although confirmation of this possibility will require further study.

Supplemental Note 3

Chromosome conformation capture analysis provides important physical information about the nature of chromosomes that can be useful for identifying genomic rearrangement events and placing contigs within chromosomes; however, this also has the potential to generate false interaction signals, because amplification or deletion of loci becomes apparent in the data as a relative increase or decrease, respectively, in observed interaction frequency. In the course of analyzing the IT subclone data, we repeatedly observed an increase in interaction over the chromosome 5 region containing the *pfmdr1* locus (Figure S9A-B). We considered the possibility that this locus, which is frequently amplified in response to antimalarial drug pressure, was duplicated in the IT isolate. Subsequent reanalysis of the large-insert libraries used to generate the IT assembly confirmed that the locus was present in three copies. Similarly, we observed an apparent interaction decrease at an internal chromosome 4 *var* gene when comparing the interactions in the A4 subclones to the 3G8 subclones (Figure S9F). Further examination of the sequence in this region revealed that a 15Kb deletion has occurred in the process of sub-cloning (Figure S9G).

This deletion is notable in that it is associated with the stable expression of an internal *var* gene (ITvar1) in the 3G8 line. While *var* gene switching does not require alterations in DNA sequence, it has been observed previously that insertions or deletions can occur with *var* gene switches (Deitsch et al., 1999; Horrocks et al., 2004) and the 3G8 clone may offer an additional instance of this. Chromosome interaction data can also be used to place genomic fragments in their proper position along the chromosome. Because observed interaction frequency scales as an inverse function of distance due to physical properties of the chromosome, this expected

decrease in interaction probability provides an immediate visual and statistical cue for the proper position for an unplaced contig. In this capacity, chromosome conformation capture provides structural information about chromosomes similar, in a sense, to techniques such as optical mapping (Cai et al., 1995), and may have additional uses in genome assembly.

Supplemental Table 1

Name	Library Type	Enzyme	Genotype	Total Reads	Mapped	Split	% Split
A4+	HiC	MboI	A4	31133540	23564242	10000348	42.44%
A4-	HiC	MboI	A4	31505940	23736139	11567994	48.74%
3G8	HiC	MboI	A4	53447418	41345734	16733368	40.47%
A4+	GCC	MboI	A4	18988066	16012792	2653450	16.57%
A4-	GCC	MboI	A4	19019824	16049291	2665960	16.61%
3G8	GCC	MboI	A4	20400656	17099629	2956534	17.29%
A4+	HiC	HindIII	A4	35389790	27738673	7123934	25.68%
A4-	HiC	HindIII	A4	46507926	35497679	12064480	33.99%
3G8	HiC	HindIII	A4	72229988	60754216	2051172	3.38%
A4+	GCC	HindIII	A4	19044836	16665267	596342	3.58%
A4-	GCC	HindIII	A4	20081522	17309104	887788	5.13%
3G8	GCC	HindIII	A4	20903082	18259006	812916	4.45%
A4+	Control	MboI	A4	21790310	16062185	9222322	57.42%
A4-	Control	MboI	A4	20423364	15823249	3560170	22.50%
3G8	Control	MboI	A4	34382936	28659130	4061798	14.17%
A4+	Control	HindIII	A4	25875380	20864942	1412180	6.77%
A4-	Control	HindIII	A4	9892522	2104270	1121964	53.32%
3G8	Control	HindIII	A4	21796788	18080044	1101512	6.09%
DCJ On	HiC	MboI	NF54	22252602	22147669	2129210	9.61%
DCJ Off	HiC	MboI	NF54	20386994	20207680	1907436	9.44%
B15C2	HiC	MboI	NF54	15331312	15219702	1983654	13.03%
DCJ On	GCC	MboI	NF54	21259398	21233219	8100656	38.15%
DCJ Off	GCC	MboI	NF54	18474310	18279129	7998312	43.76%
B15C2	GCC	MboI	NF54	16674282	16639567	7663138	46.05%
DCJ On	HiC	HindIII	NF54	22252602	22147669	1402092	6.33%
DCJ Off	HiC	HindIII	NF54	32339184	31442882	2662672	8.47%
B15C2	HiC	HindIII	NF54	19636634	19495659	2108744	10.82%
DCJ On	GCC	HindIII	NF54	17082948	17052156	1214964	7.12%
DCJ Off	GCC	HindIII	NF54	17528200	17275477	1672316	9.68%
B15C2	GCC	HindIII	NF54	15929652	15892292	1201134	7.56%
NF54	Control	MboI	NF54	20562618	20551753	8973524	43.66%
NF54	Control	HindIII	NF54	15699790	15693363	1332778	8.49%
A4 pilot	GCC	HindIII	A4	35569274	23140514	1301762	5.63%
A4 pilot	GCC	MboI	A4	57805960	46213445	5606754	12.13%
A4 pilot	HiC	HindIII	A4	34027340	26760830	7583842	28.34%
A4 pilot	HiC	MboI	A4	61333256	45872915	22773024	49.64%

Figure S1

A)

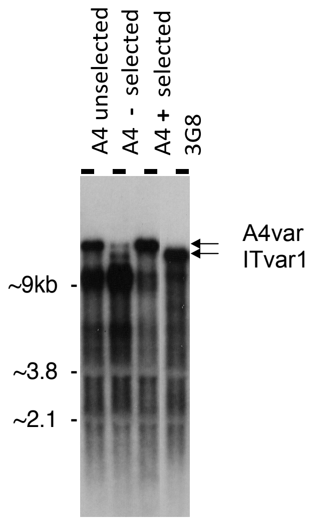

B)

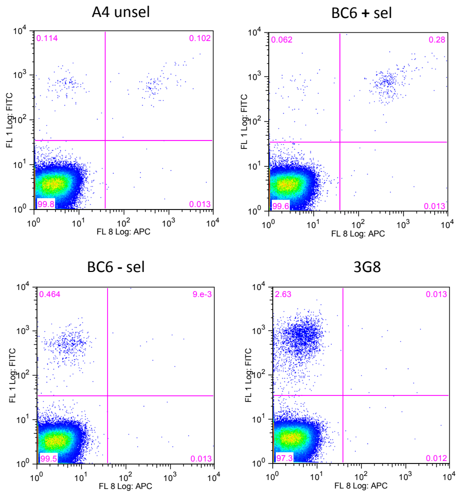

C)

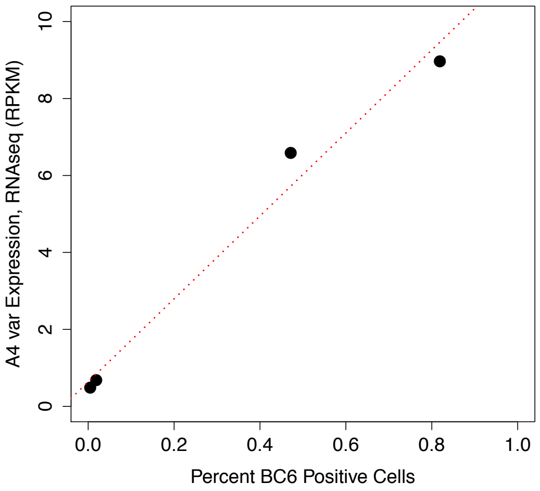

D)

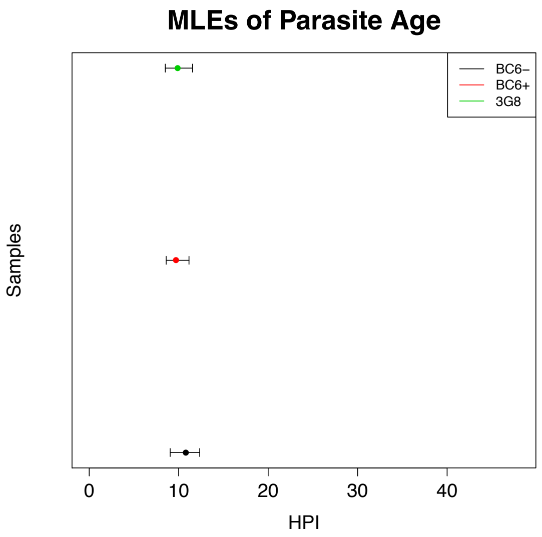
MLEs of Parasite Age
BC6−
BC6+
3G8
Samples
0
10
20
30
40
HPI

E)

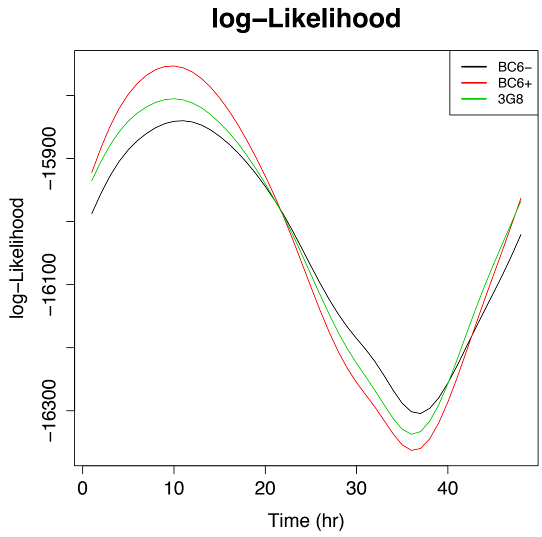
log−Likelihood
BC6−
BC6+
3G8
log−Likelihood
−15900
−16100
−16300
0
10
20
30
40
Time (hr)

F)

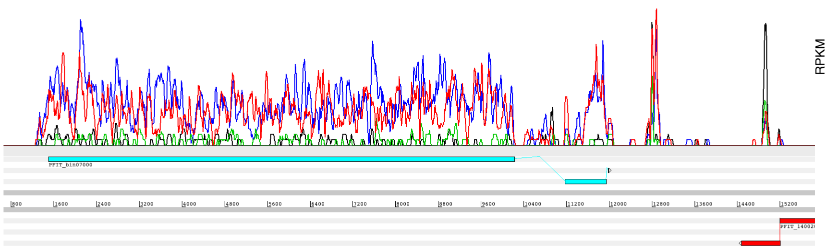

G)

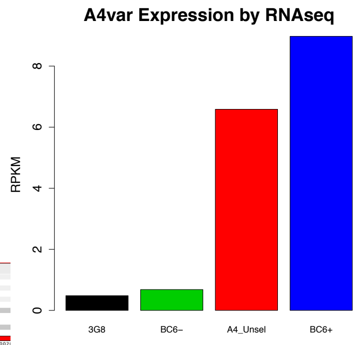
A4var Expression by RNAseq
RPKM
0
2
4
6
8
3G8
BC6−
A4_Unsel
BC6+

H)

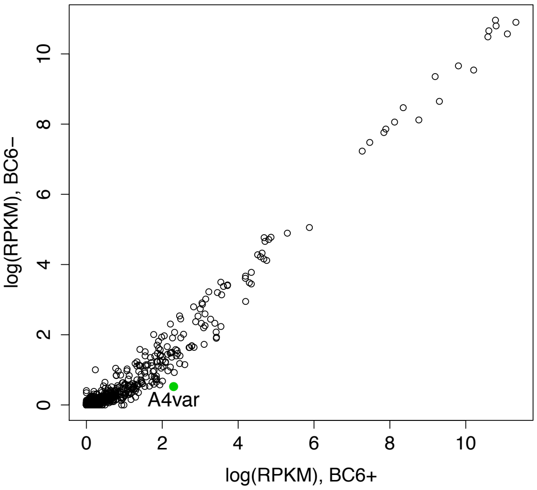
log(RPKM), BC6−
0
2
4
6
8
10
A4var
log(RPKM), BC6+

I)

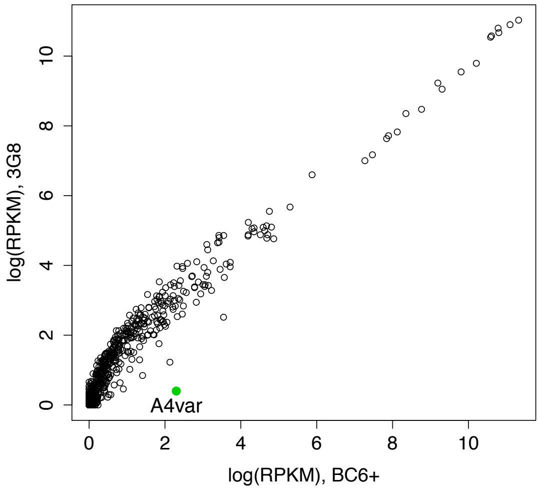
log(RPKM), 3G8
0
2
4
6
8
10
A4var
log(RPKM), BC6+

Figure S1: Confirmation of *var* gene and PfEMP1 protein expression. Northern blots probed with a conserved *var* exon 2 probe are shown in **A).** Flow cytometry of the corresponding lines, showing infected and uninfected red blood cells stained with SYBR green and the BC6 antibody (with an APC-conjugated secondary antibody) is shown in **B).** Panel **C)** shows the concordance between *A4var* gene expression as measured by RNAseq and the percentage of BC6+ cells by flow cytometry. The stage estimation method described previously (Lemieux et al., 2009) was used to estimate an approximate temporal progression, in hours post invasion (HPI) for the samples used for RNAseq and chromosome conformation capture. The log-likelihood curves are shown in panel **D)** and maximum-likelihood esimates and 95% confidence intervals are given in **E),** confirming that the samples studied were at mid-to-late ring stage and possessed equivalent age and synchronicity. **F)** RNAseq coverage of the *A4var* gene in the unselected A4 clone of the IT isolate (red line), BC6 positively selected A4 ("BC6+", blue line), BC6 negatively selected A4 ("BC6-", green line), and 3G8 (black line). **G)** Reads per kilobase of transcript per million mapped reads (RPKM) values for the *A4var* gene. **H)** and **I)** are scatterplots of gene expression for genes on chromosome 13 showing differential expression of the *A4var* gene in the BC6+ vs. the BC6- and 3G8-line.

Figure S2

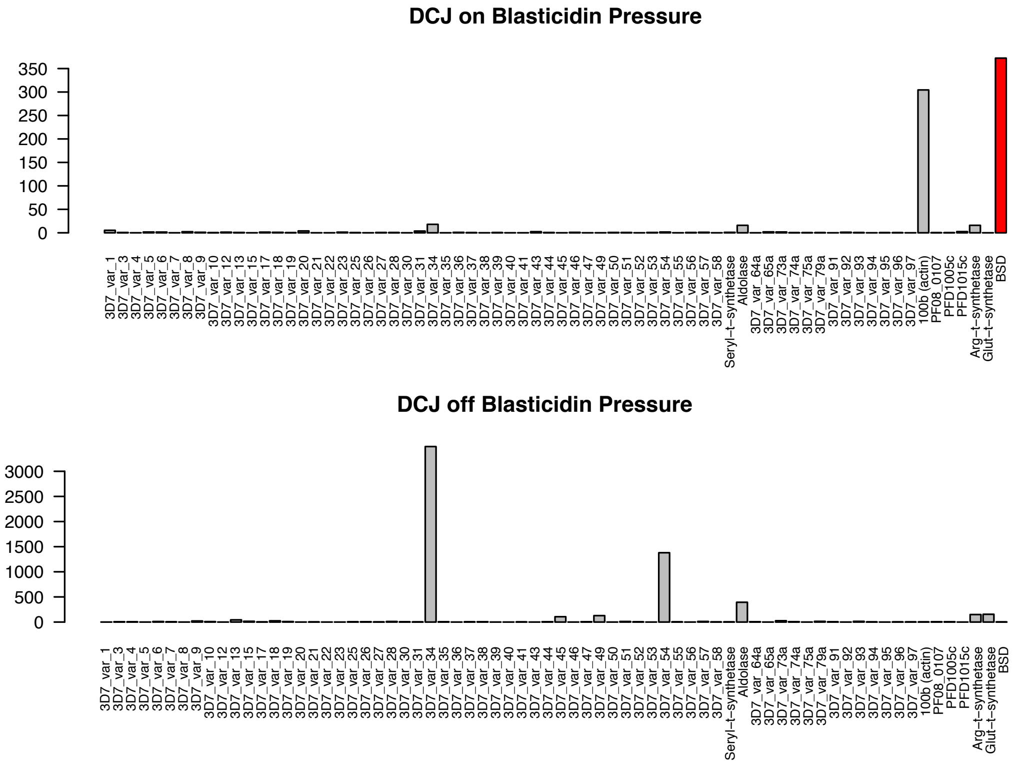

Figure S2: Analysis of *var* gene expression in DCJ lines using quantitative real-time PCR. The primer sets from (Salanti et al., 2003) with modifications by (Dzikowski et al., 2006) were used to profile the *var* gene expression patterns in the DCJ isolates grown with and without blasticidin S. Consistent with the expression patterns described for this subclone under these conditions in Dzikowski et al. 2006, the DCJ parasite grown without blasticidin S expresses a *var* gene detected by primer set #34 from Salanti et al. which detects PFD1015c. As noted by the authors in reference (Salanti et al., 2003), this *var* gene is also partially cross-reactive with their primer set 54, which detects PFD1005c and PFD1015c. When the DCJ subclone is grown in the presence of blasticidin S, strong expression of the *var*BSD gene is detectable (red bar). Quantification is by the ΔΔCt method and is relative to Seryl-t-synthetase, which is arbitrarily set to a value of 1.

Figure S3

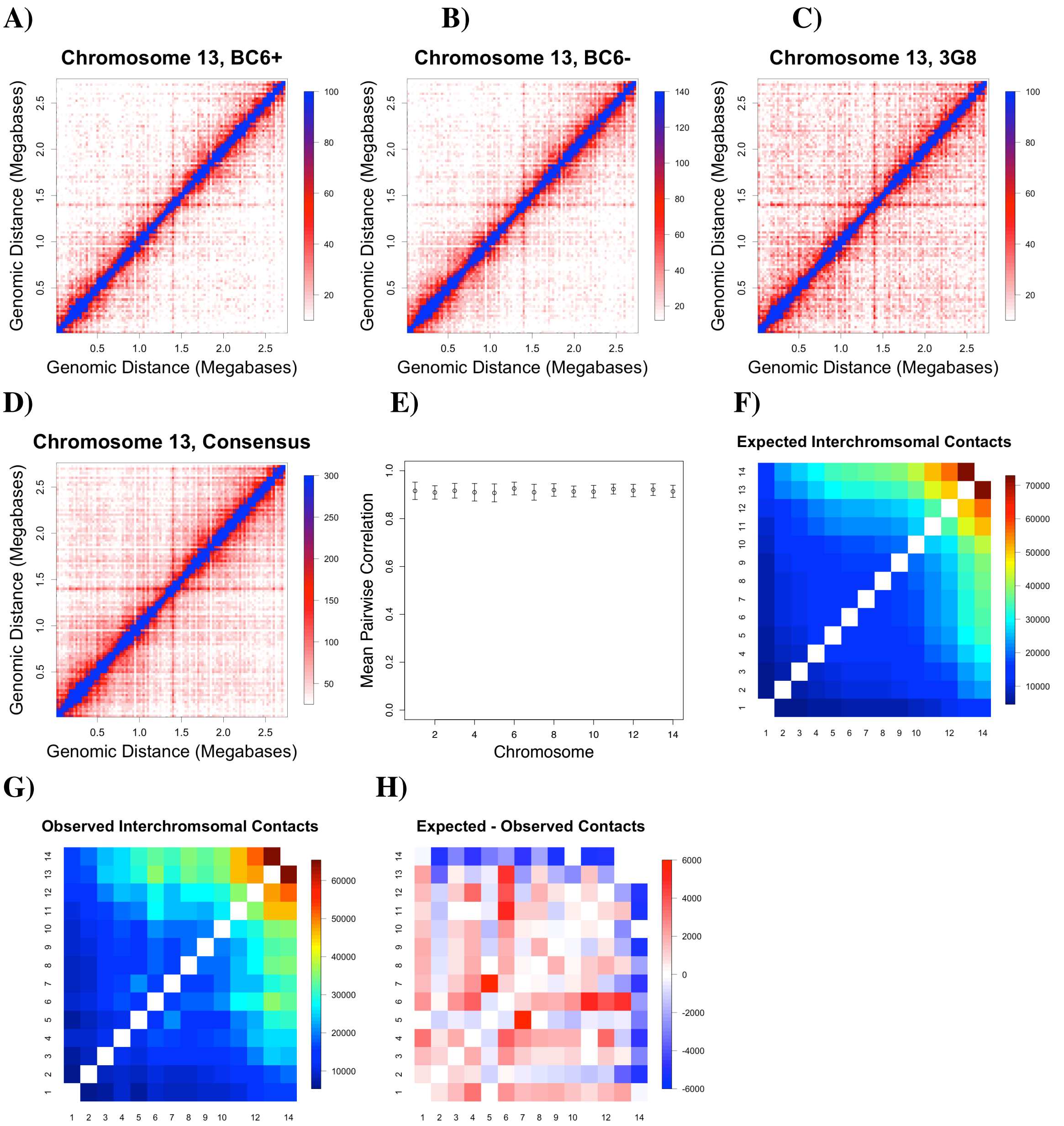

Figure S3: The intra-chromosomal interaction matrices for chromosome 13 in the BC6+, BC6-, and 3G8 libraries (panels **A**, **B**, and **C** respectively, plotted as in Figure 2). Most spatial interactions occur *in cis* between nearby sequences, reflecting the polymeric nature of chromosomes. Some evidence for regional variation in interaction density can be seen, with the diagonal increasing and decreasing in 'thickness' down the length of the chromosome. **D)** Shows the consensus interaction matrix for chromosome generated by combining the libraries in **A-C**. Panel **E)** plots the mean (+/- 1SD) of the pairwise correlation coefficients between chromosomes in the three libraries, computed using vectors formed by concatenating the rows or columns of

the interaction matrix. Because the matrices are symmetric, the elements above the diagonal were discarded in calculating the correlation coefficient. For this analysis, 5Kb bins were used to provide greater sensitivity in detecting changes. The mean pairwise correlation coefficient is not significantly different for Chromosome 13, even though this chromosome has an active *A4var* gene in the BC6+ libraries, suggesting that chromosome-wide reconfigurations do not occur with *var* switching. **F-G) Expected and Observed Inter-chromosomal Interaction Matrices. F)** A heatmap of expected interactions under a model in which contact between chromosomes occurs randomly with probability proportional to the product of their lengths. **G)** The observed inter-chromosomal interactions for the NF54 libraries, showing strong concordance with the values in **F** (r = 0.98). **H)** The difference in read counts between observed and expected values for each chromosome pair. The largest difference belongs to the chromosome 5, chromosome 7 interaction pair (5840 reads). The apparent paucity of observed interactions for chromosomes 2 and 14 is explained by the loss of the left arm of chromosome 2 (KAHRP deletion) and the right-hand side of chromosome 14 in continuous culture (visible in Figure 2 and the individual chromosome plots in Figure S4B).

Figure S4

A)

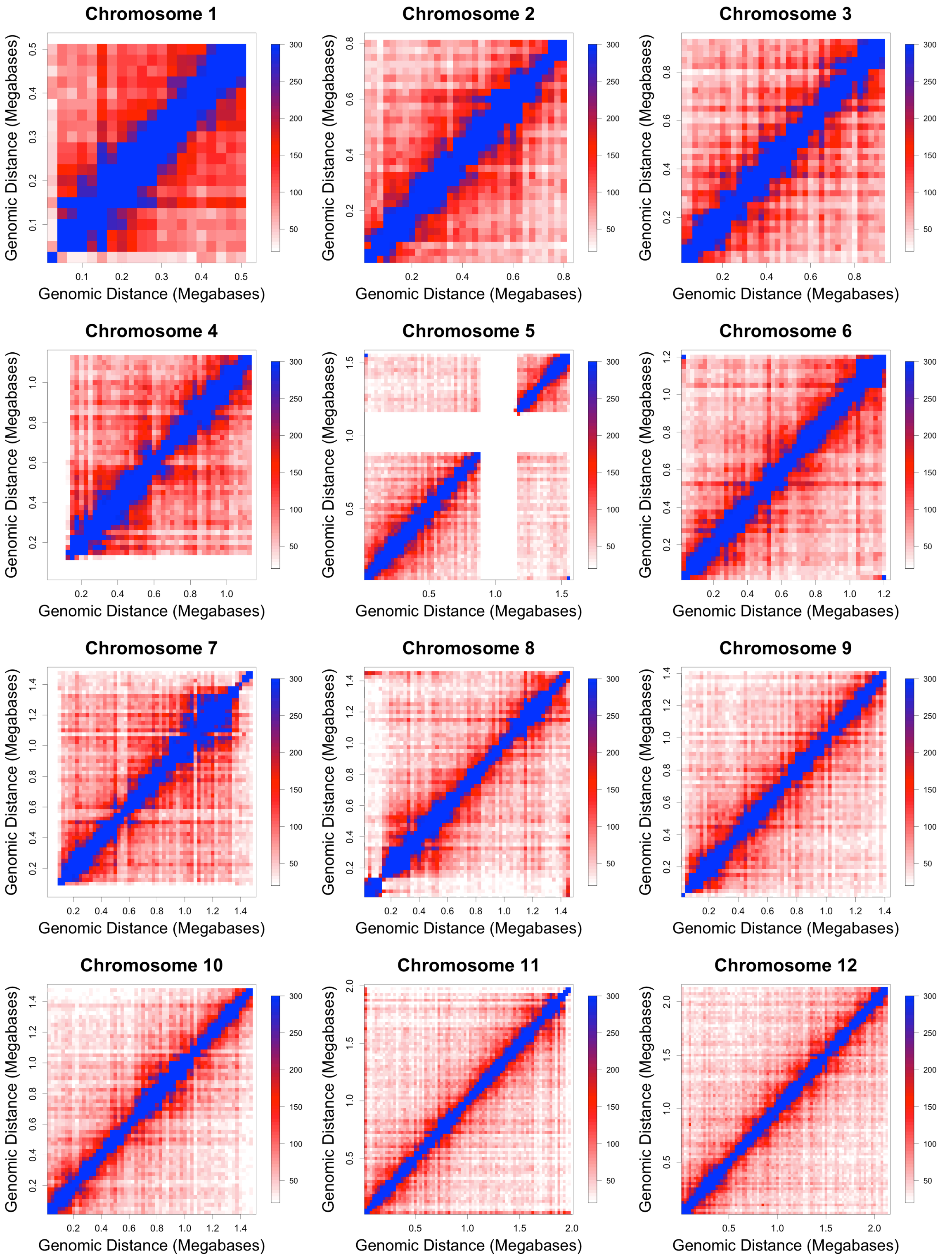
Chromosome 1
Chromosome 2
Chromosome 3
Chromosome 4
Chromosome 5
Chromosome 6
Chromosome 7
Chromosome 8
Chromosome 9
Chromosome 10
Chromosome 11
Chromosome 12
Genomic Distance (Megabases)
Genomic Distance (Megabases)
300
250
200
150
100
50

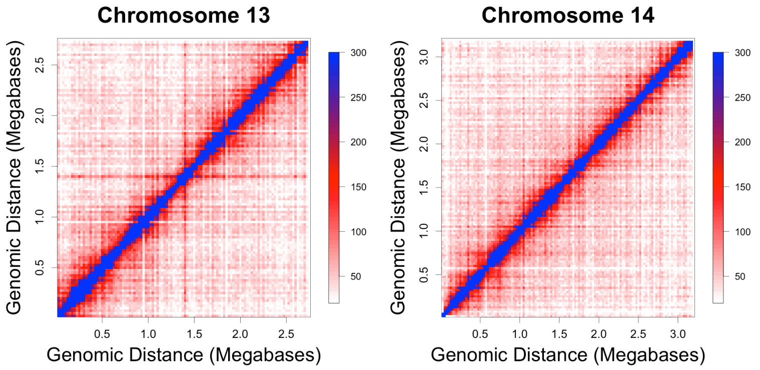

Chromosome 13
Chromosome 14
Genomic Distance (Megabases)
Genomic Distance (Megabases)
Genomic Distance (Megabases)
Genomic Distance (Megabases)
0.5
1.0
1.5
2.0
2.5
3.0
50
100
150
200
250
300

B)

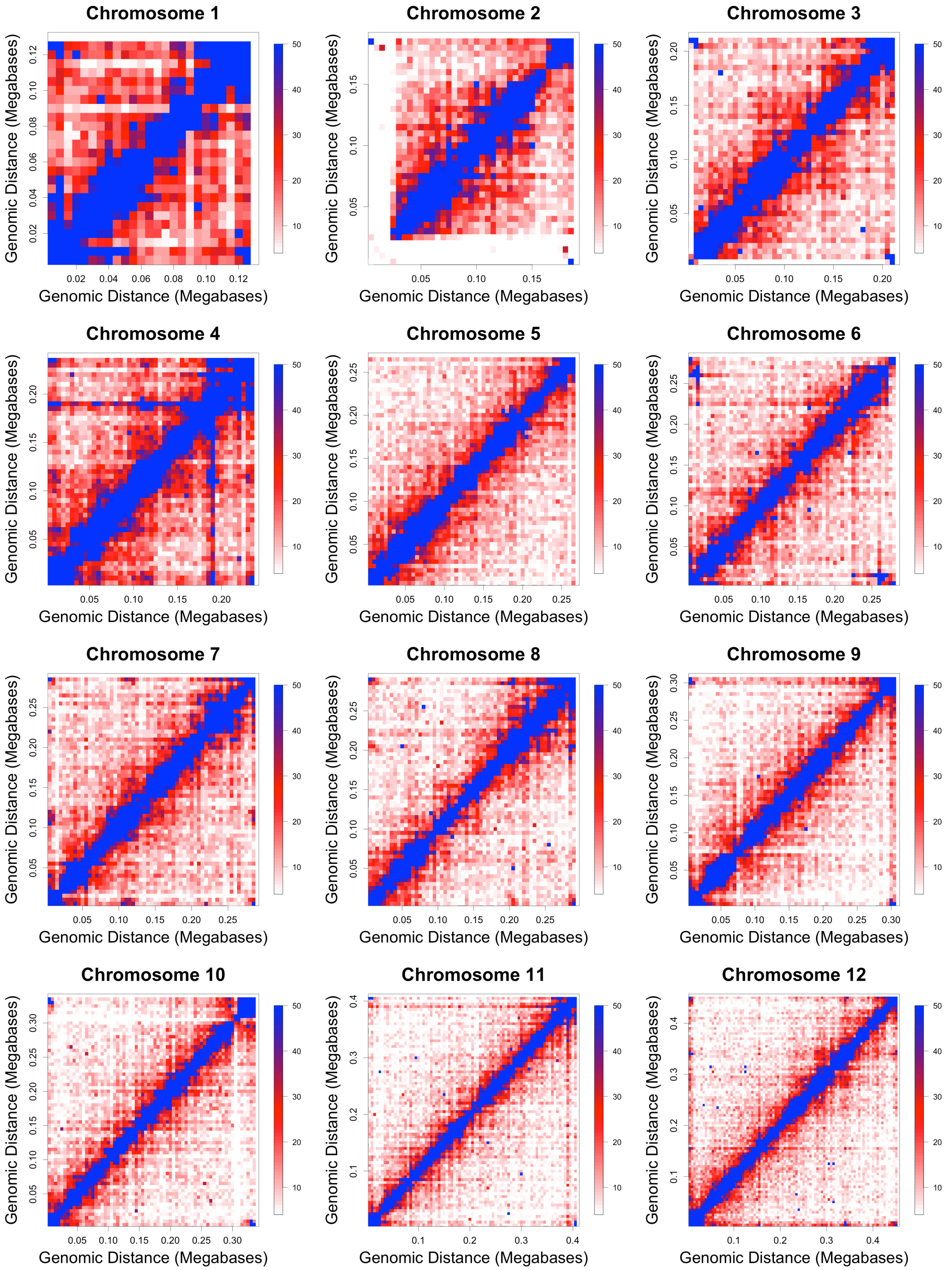

Chromosome 1
Chromosome 2
Chromosome 3
Chromosome 4
Chromosome 5
Chromosome 6
Chromosome 7
Chromosome 8
Chromosome 9
Chromosome 10
Chromosome 11
Chromosome 12
Genomic Distance (Megabases)
10
20
30
40
50

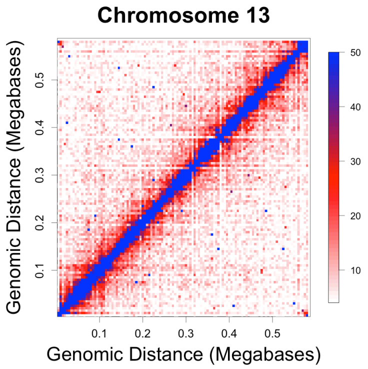
Chromosome 13
Genomic Distance (Megabases)
Genomic Distance (Megabases)
0.1
0.2
0.3
0.4
0.5
50
40
30
20
10

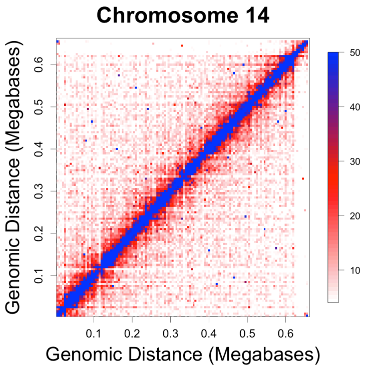
Chromosome 14
Genomic Distance (Megabases)
Genomic Distance (Megabases)
0.1
0.2
0.3
0.4
0.5
0.6
50
40
30
20
10

C)

1)

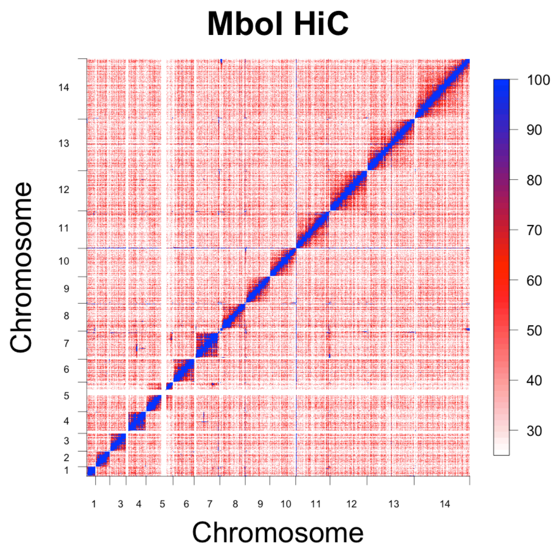
MboI HiC
Chromosome
Chromosome
1 2 3 4 5 6 7 8 9 10 11 12 13 14
100 90 80 70 60 50 40 30

2)

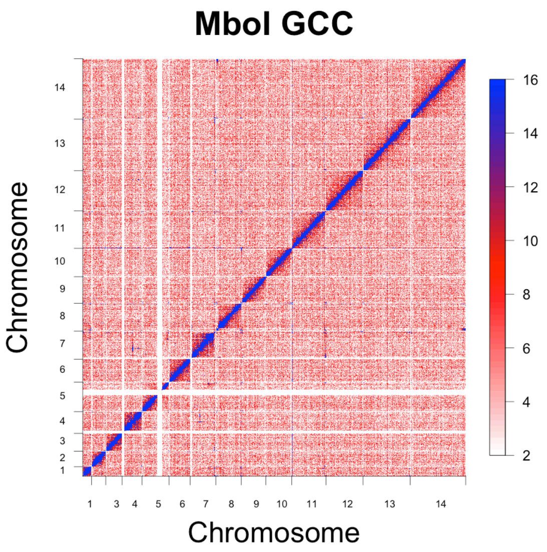
MboI GCC
Chromosome
Chromosome
1 2 3 4 5 6 7 8 9 10 11 12 13 14
16 14 12 10 8 6 4 2

3)

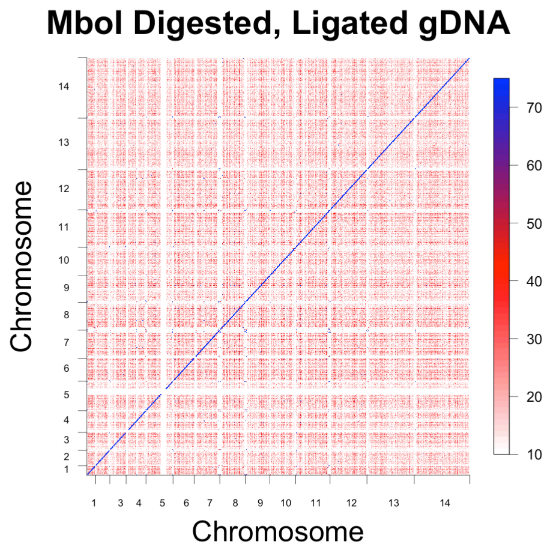
MboI Digested, Ligated gDNA
Chromosome
Chromosome
1 2 3 4 5 6 7 8 9 10 11 12 13 14
70 60 50 40 30 20 10

4)

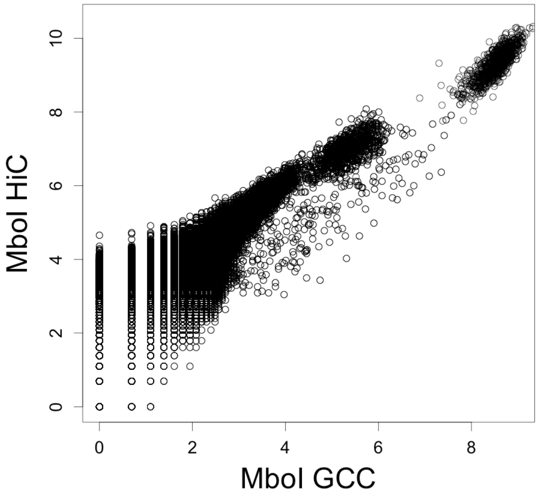
MboI HiC
MboI GCC
0 2 4 6 8 10
0 2 4 6 8

5)

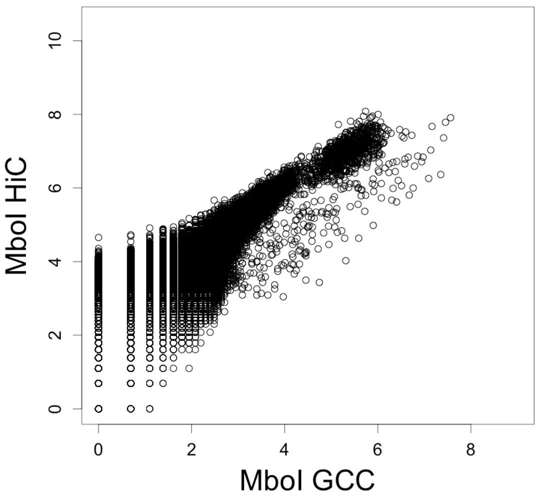

Mbol HiC
Mbol GCC
0
2
4
6
8
10

6)

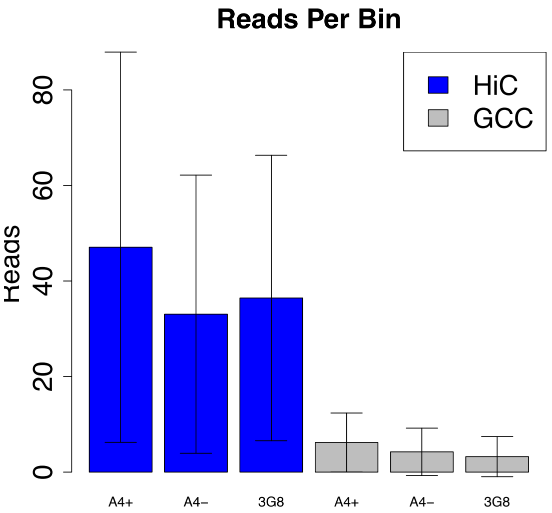

Reads Per Bin
Reads
HiC
GCC
0
20
40
60
80
A4+
A4−
3G8
A4+
A4−
3G8

D)

1)

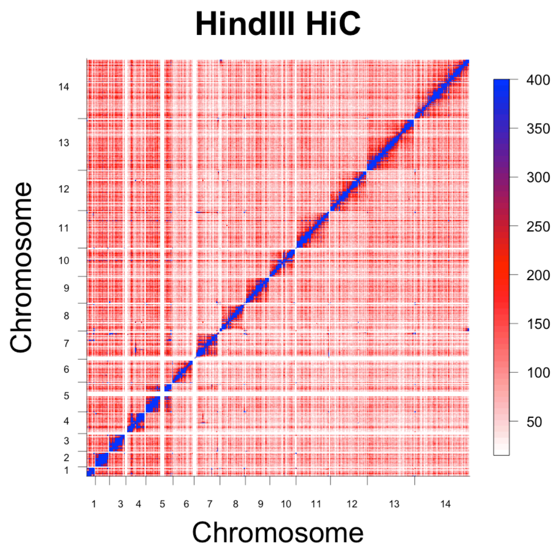

HindIII HiC
Chromosome
Chromosome
1 2 3 4 5 6 7 8 9 10 11 12 13 14
400 350 300 250 200 150 100 50

2)

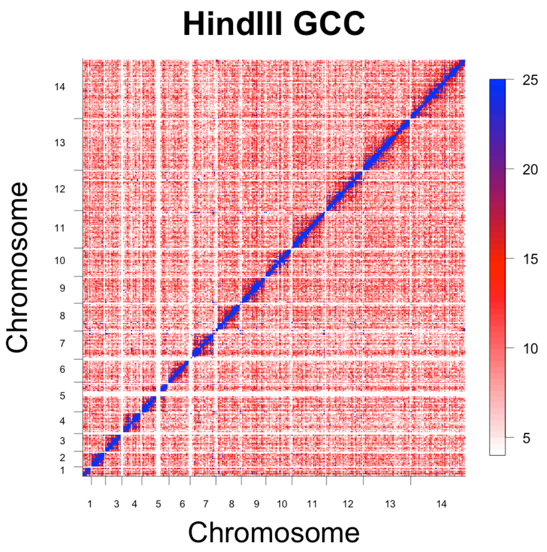

HindIII GCC
Chromosome
Chromosome
1 2 3 4 5 6 7 8 9 10 11 12 13 14
25 20 15 10 5

3)

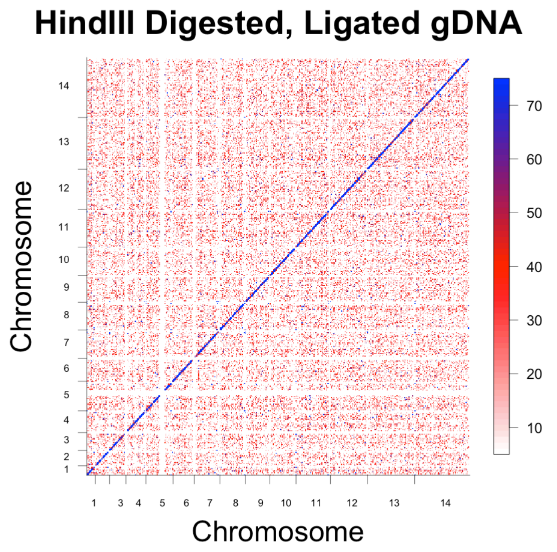

HindIII Digested, Ligated gDNA
Chromosome
Chromosome
1 2 3 4 5 6 7 8 9 10 11 12 13 14
70 60 50 40 30 20 10

4)

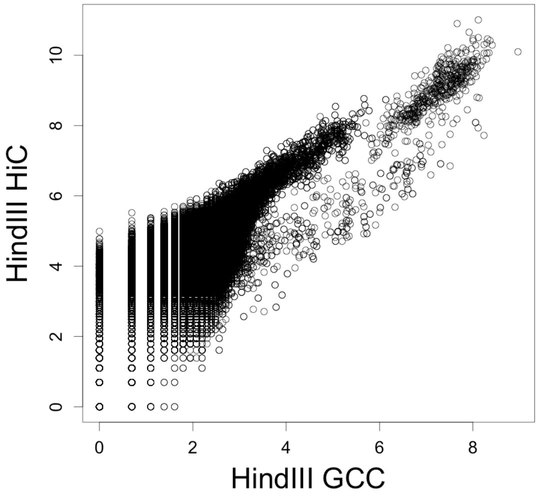

HindIII HiC
HindIII GCC
0 2 4 6 8 10
0 2 4 6 8

E)

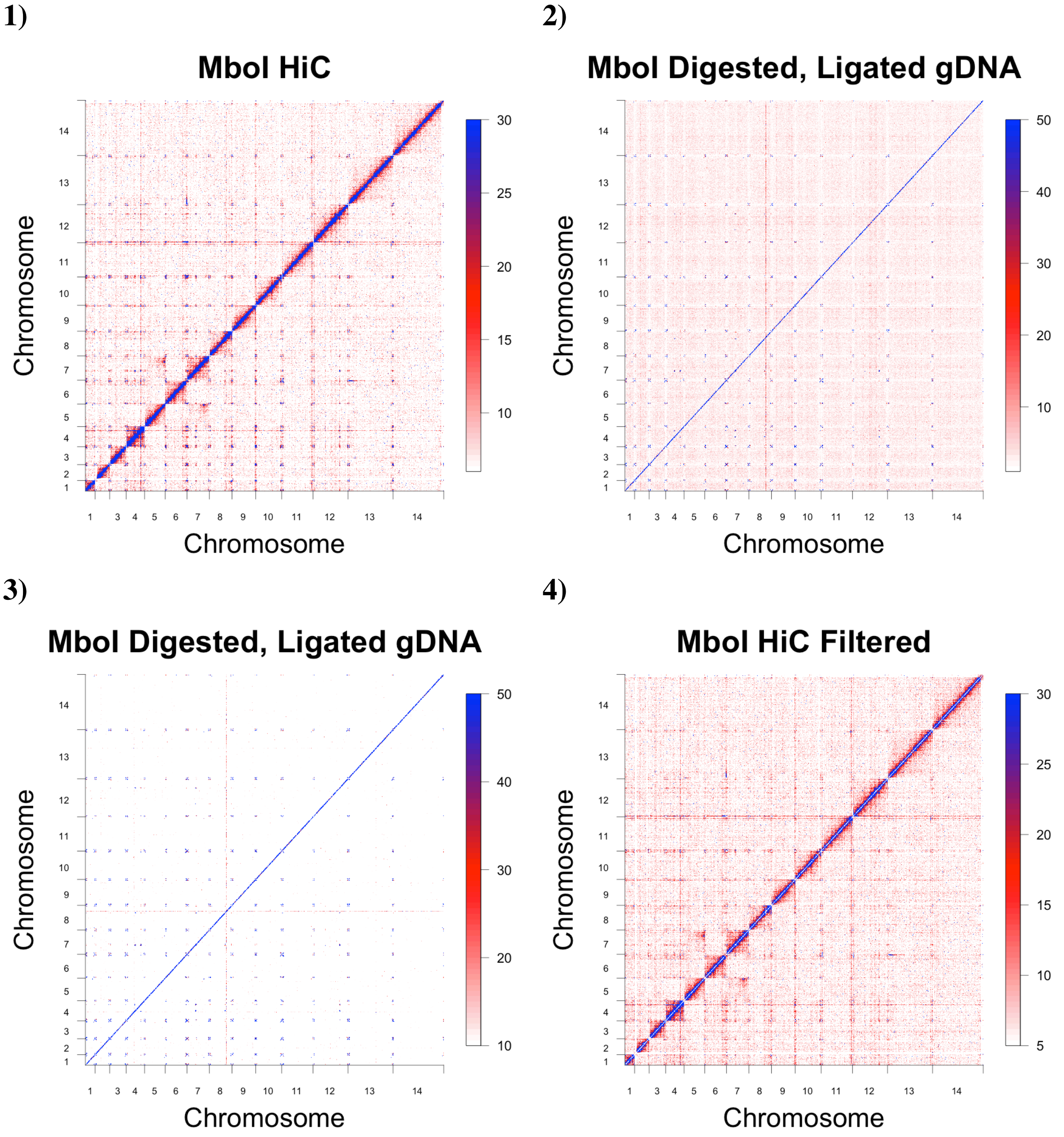

1)
MboI HiC
2)
MboI Digested, Ligated gDNA
3)
MboI Digested, Ligated gDNA
4)
MboI HiC Filtered
Chromosome
Chromosome

5)

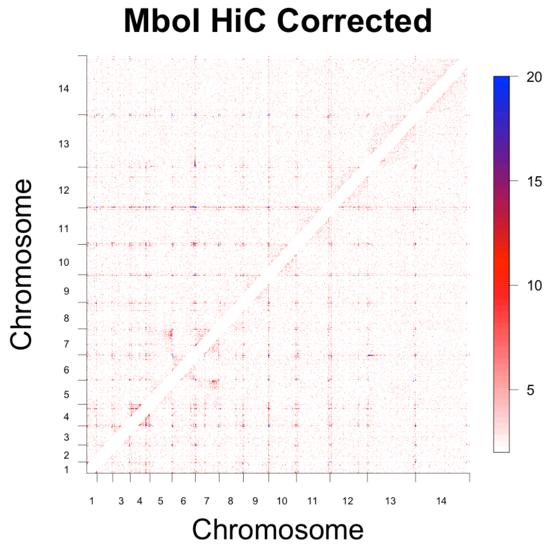
MboI HiC Corrected
Chromosome
Chromosome
14
13
12
11
10
9
8
7
6
5
4
3
2
1
1
3
4
5
6
7
8
9
10
11
12
13
14
20
15
10
5

F)

1)

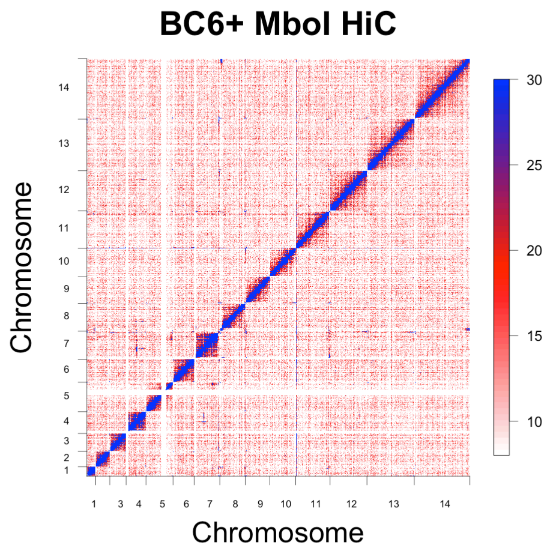
BC6+ MboI HiC
Chromosome
Chromosome
30
25
20
15
10

2)

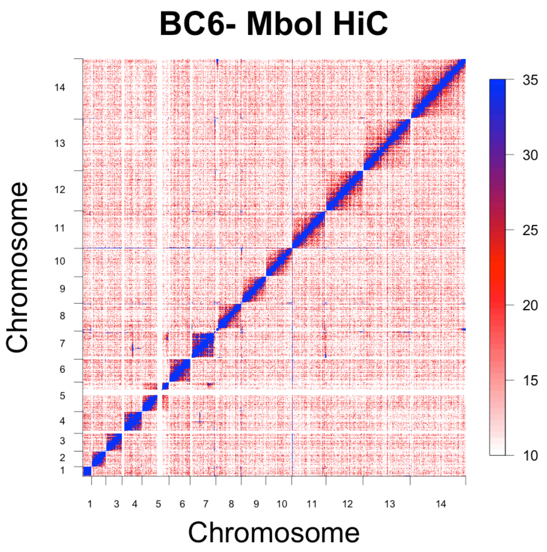
BC6- MboI HiC
Chromosome
Chromosome
35
30
25
20
15
10

3)

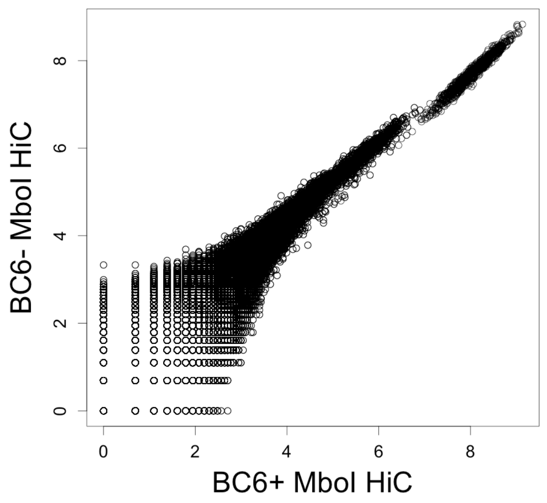
BC6- MboI HiC
BC6+ MboI HiC

G)

1)

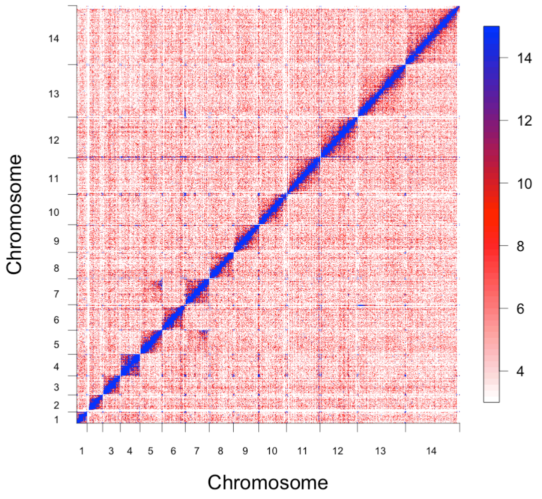

Chromosome
Chromosome
14
13
12
11
10
9
8
7
6
5
4
3
2
1

2)

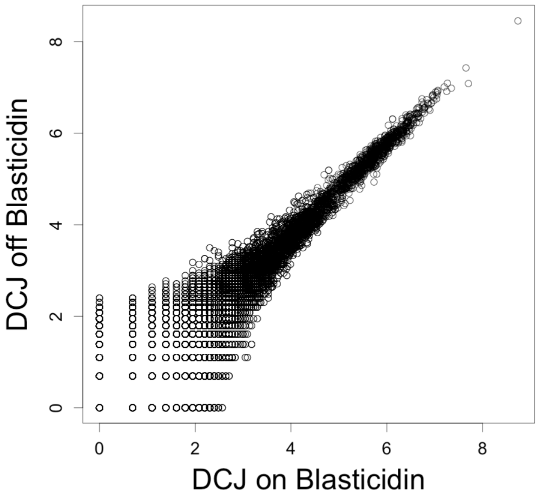

DCJ off Blasticidin
DCJ on Blasticidin

3)

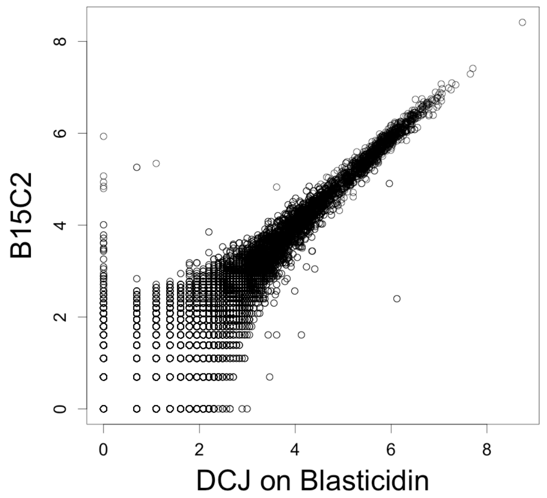

B15C2
DCJ on Blasticidin

4)

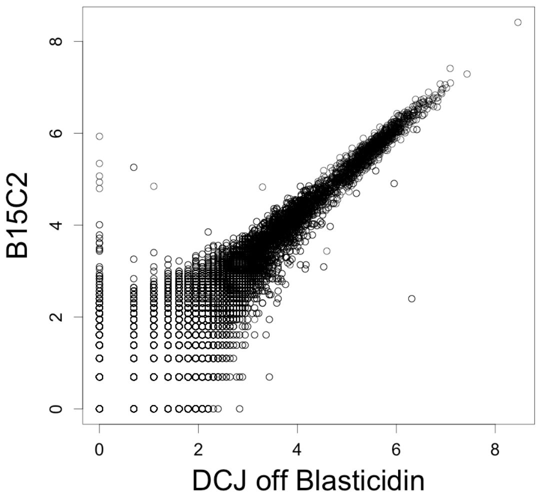

B15C2
DCJ off Blasticidin

Figure S4: Interaction matrices. A) Interaction matrices for the combined IT MboI HiC libraries at a resolution of 25Kb for individual chromosomes. **B)** Interaction matrices for the DCJ and B15C2 MboI HiC libraries at a resolution of 25Kb. **C)** Comparison of MboI HiC and GCC whole genome interaction matrices at a resolution of 25Kb for HiC (panel 1) and GCC (panel 2) as well as digested, ligated control gDNA (panel 3) for the combined IT isolates. A scatterplot of vectorized interaction matrices is shown in panel 4 (r = 0.97) on the log scale. The cloud of points in the upper right of the scatterplot, with a different slope, is due to points along the matrix diagonal (i.e. A_{ii}). This can be seen by removing the elements along the matrix diagonal and plotting the vectorized "hollow" matrix which is shown in panel 5. Panel 6 shows the mean +/- standard deviation of the reads per bin in the first 300 Kb of the chromosomes (with the first 50Kb removed in order to filter self-ligation products), the region which contains the vast majority of reads corresponding to genuine polymer contacts, in the A4 MboI HiC and GCC libraries. A higher number of reads was observed in the HiC libraries (mean for the three HiC libraries in panel 6: 38.9 +/- 7.3) as compared to the GCC libraries (mean for GCC libraries in panel 6: 4.6 +/- 1.5). **D)** Whole genome interaction matrices constructed for the combined IT isolates using HindIII and binned at a resolution of 50Kb for HiC (panel 1) and GCC (panel 2) as well as digested, ligated control gDNA (panel 3). A scatterplot of vectorized interaction matrices is shown in panel 4 (r = 0.88). **E)** HiC maps for the combined DCJ and B15C2 lines are shown in panel 1. In these maps, reads that mapped to more than one location were randomly placed at one of their mapping sites. Because of the high degree of homology between subtelomeric regions, this creates potential for spurious interactions resulting from sequence similarity. For this reason, we also examined split read-pair mapping in digested and ligated gDNA controls (Panel 2). We selected the bins that scored in the highest 1% of read counts in the control libraries (all non-white bins in Panel 3), and removed these from bins from the subsequent analysis to create a 'filtered' HiC map, shown in panel 4. This approach was meant to be conservative and reduce the rate of false positive interactions generated by sequence homology. One drawback of this approach is that the genuine information in these bins was not used. Therefore, the maps in panel 4 may underestimate the extent of genuine interaction between subtelomeric regions. The matrix in panel 5 represents a correction introduced by subtracting the normalized control matrix from the normalized interaction matrix, after removing short-range interactions (see Supplemental Note 1). Figure **F)** shows a comparison of maps between the BC6+ and BC6- lines. The MboI HiC maps at a bin resolution of 25Kb are shown for BC6+ in panel 1 and Bc6- in panel 2 A scatterplot, on the log scale, shows the high degree of reproducibility of this technique (r = 0.99) for isogenic lines as well as the limited observed differences between lines confirmed for distinct *var* expression phenotypes. As mentioned previously, this level of reproducibility between maps constructed from isolates of the same genetic background allowed us to combine datasets to produce consensus maps of higher signal-to-noise ratio. **G)** Panel 1 shows consensus maps formed from the DCJ and B15C2 isolates in which reads mapping to multiple sites in the genome were discarded from the analysis. These maps are in excellent agreement with the filtered maps in panel 4 of **E),** suggesting that discarding degenerately mapping reads has a similar effect to removing from the analysis elements that generate a positive signal in the control libraries. Panels 2- 4 of **G)** show comparisons between the reads per bin in the DCJ Off, DCJ On and B15C2 libraries. The concordance between lines (panel 2, r = 0.984; panel 3, r = 0.978; panel 4, r = 0.975) demonstrates the technical reproducibility of the technique as well as biological similarity in the folding architecture between lines expressing different *var* loci, discussed in the

main text. As observed for the IT isolates, the strongest reproducibility was seen between lines of identical genetic background (DCJ On vs. Off).

Figure S5

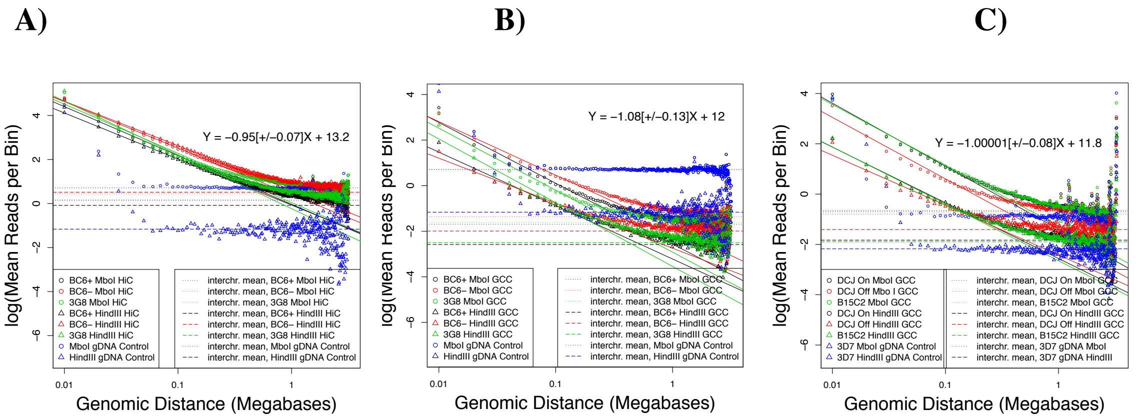

Figure S5: Relationship between log(split read-pairs) and log(genomic distance) in HiC and GCC libraries and controls. As in Figure 3B, the logarithm of the number of split read-pairs is shown on the y-axis and the logarithm of genomic distance is shown on the x-axis. A linear fit is shown on the log-log scale for each of the libraries. We obtained values of approximately -1 for all: Panel **(A)** shows the relationship for the A4 HiC libraries (mean scaling exponent: -0.95 +/- 0.07); panel **(B)** contains the A4 GCC libraries (mean scaling exponent: -1.08 +/- 0.13); and **(C)** shows the GCC libraries generated in the NF54-derived clones (mean scaling exponent -1.0001 +/- 0.08). Mean reads per bin for interchromosomal interactions are plotted as horizontal dashed lines for each library. Control libraries (plotted in blue) did not show linear scaling on a log-log plot in any of the assays, and instead showed an exponential decrease in contact probability for the first 10 - 50 KB, followed by a curve of no slope. This exponential decrease in control libraries likely results from missed cleavages, which by definition follow a geometric distribution, $(1 - p)^{n-1}p$, where p is the probability of a successful cleavage and n is the number of cleavage sites. The second portion of the curve results from random contacts, which should show no scaling as a function of distance. The interaction libraries (HiC and GCC) are expected to contain a sum of linear scaling due to polymeric contacts (Supplemental Note 2) and non-specific scaling due to experimental noise such as present in the control libraries. Thus, the HiC libraries, which contain larger numbers of split read pairs, show linear scaling over the first ~500 Kb, whereas the GCC libraries begin to deviate from the linear scaling relationship after approximately 300Kb, reflecting a weaker signal-to-noise ratio.

Figure S6

A)

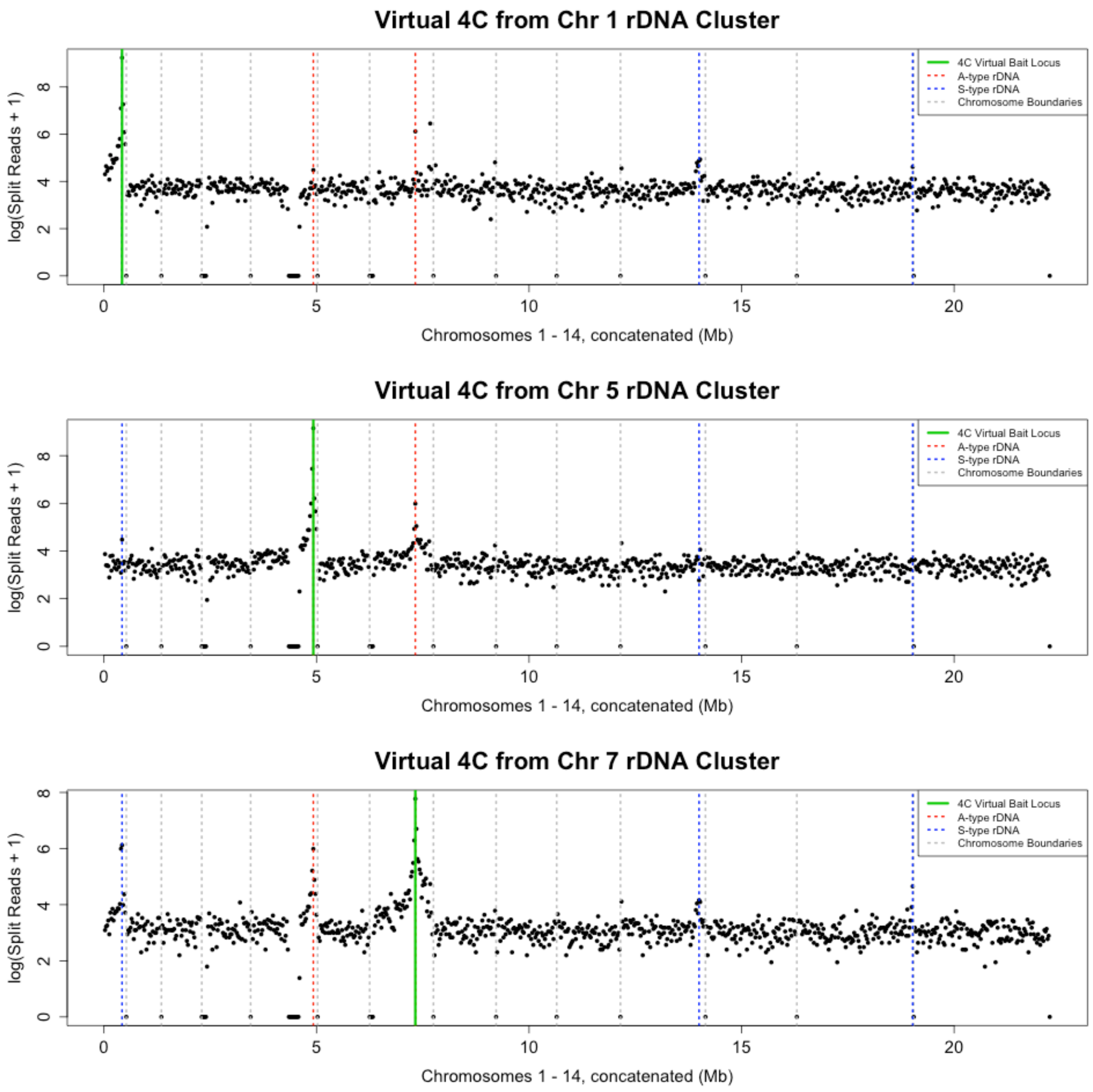

Virtual 4C from Chr 1 rDNA Cluster
log(Split Reads + 1)
Chromosomes 1 - 14, concatenated (Mb)
4C Virtual Bait Locus
A-type rDNA
S-type rDNA
Chromosome Boundaries
Virtual 4C from Chr 5 rDNA Cluster
log(Split Reads + 1)
Chromosomes 1 - 14, concatenated (Mb)
4C Virtual Bait Locus
A-type rDNA
S-type rDNA
Chromosome Boundaries
Virtual 4C from Chr 7 rDNA Cluster
log(Split Reads + 1)
Chromosomes 1 - 14, concatenated (Mb)
4C Virtual Bait Locus
A-type rDNA
S-type rDNA
Chromosome Boundaries

(Figure S6, ctd.)

B)

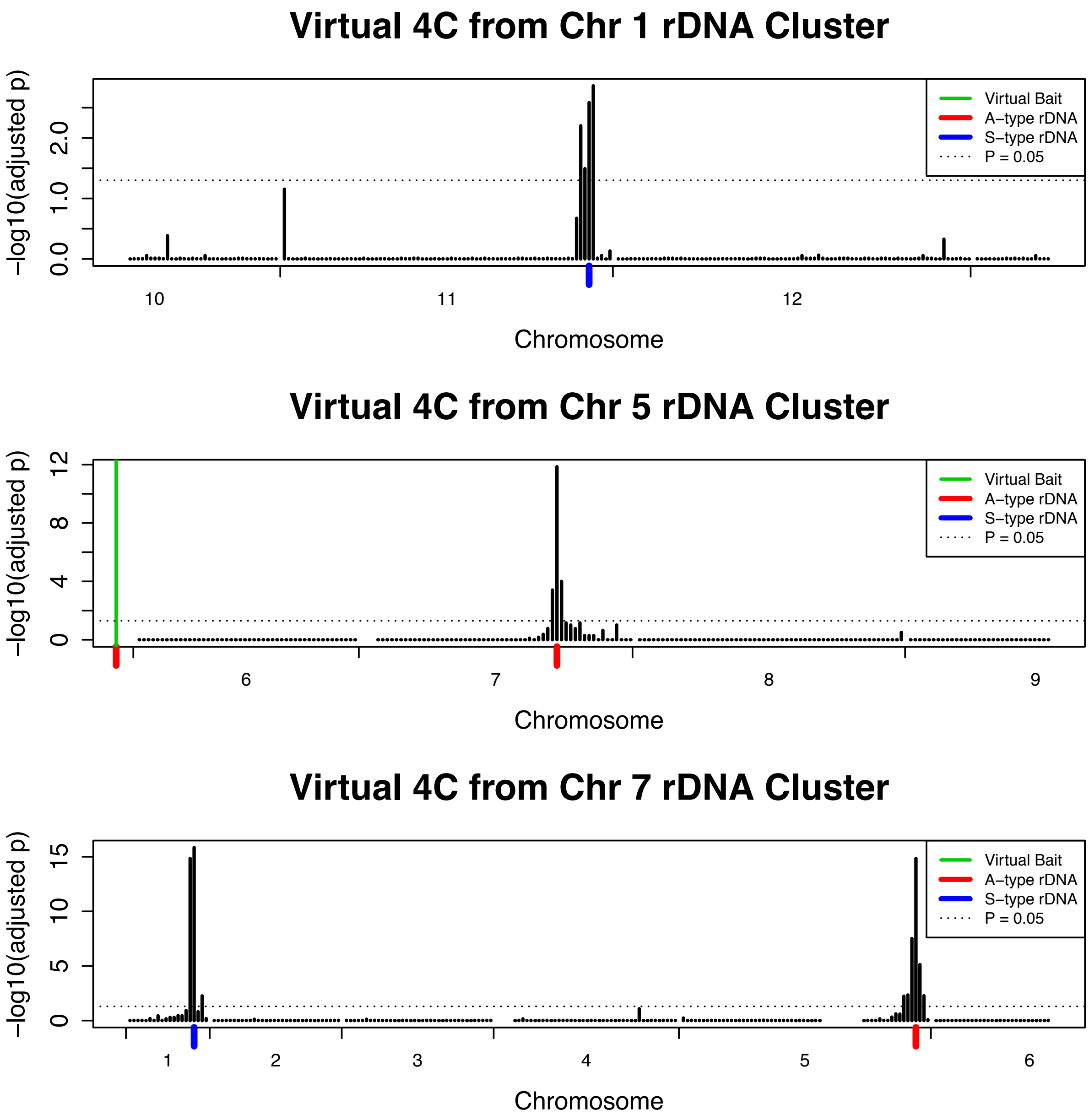

(Figure S6, ctd.)

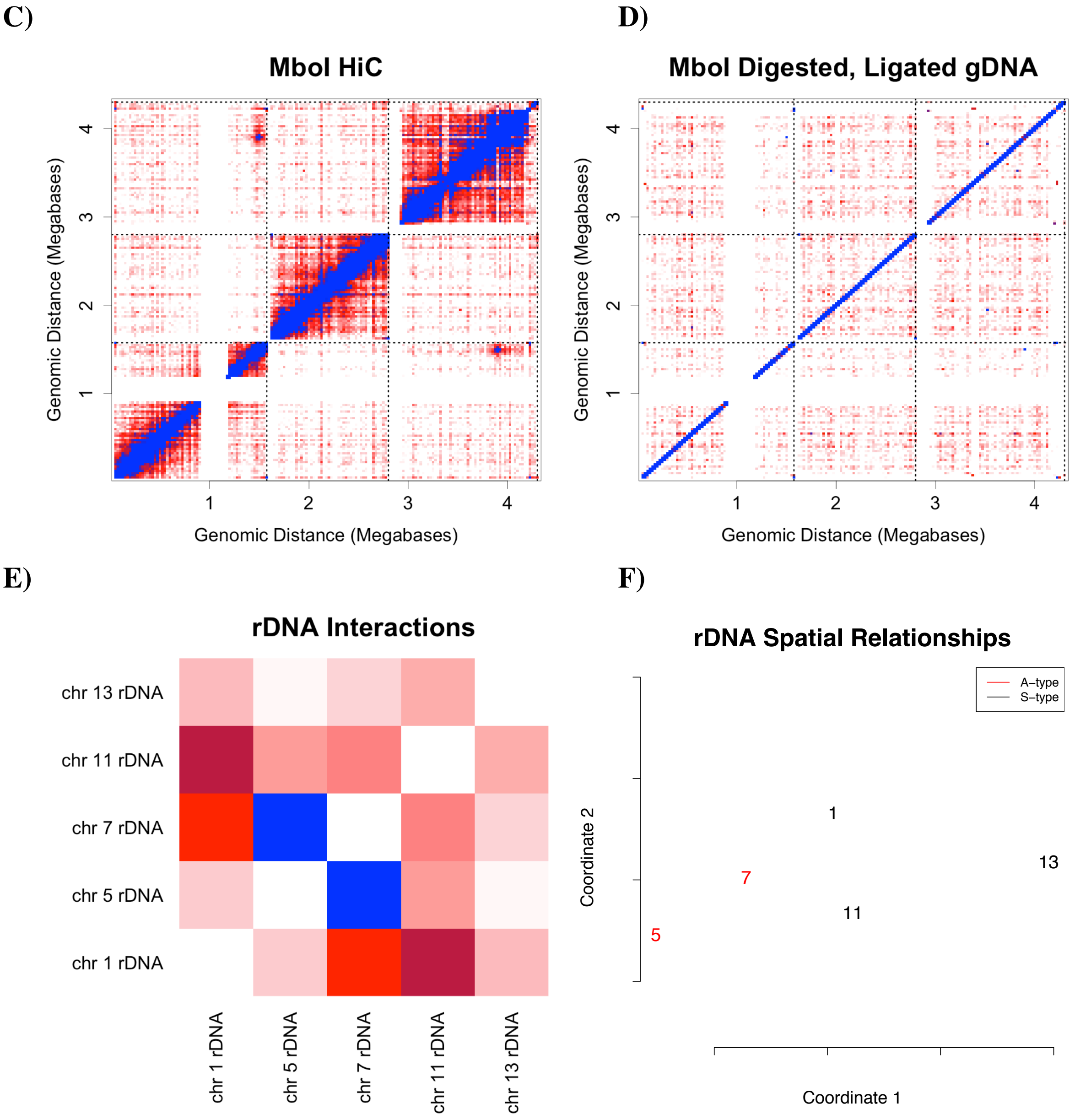

G)
1)

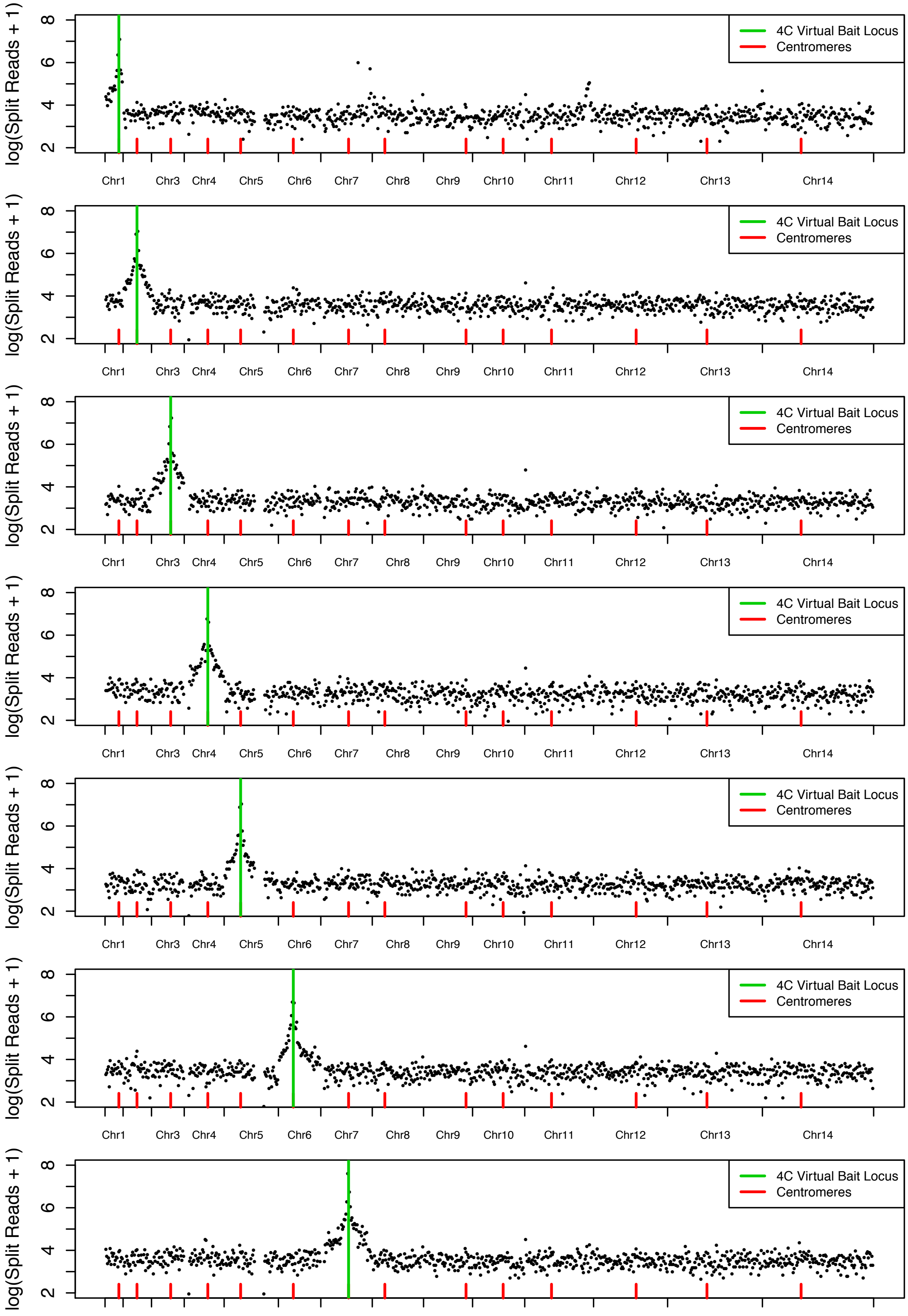
log(Split Reads + 1)
2
4
6
8
4C Virtual Bait Locus
Centromeres
Chr1
Chr3
Chr4
Chr5
Chr6
Chr7
Chr8
Chr9
Chr10
Chr11
Chr12
Chr13
Chr14

2)

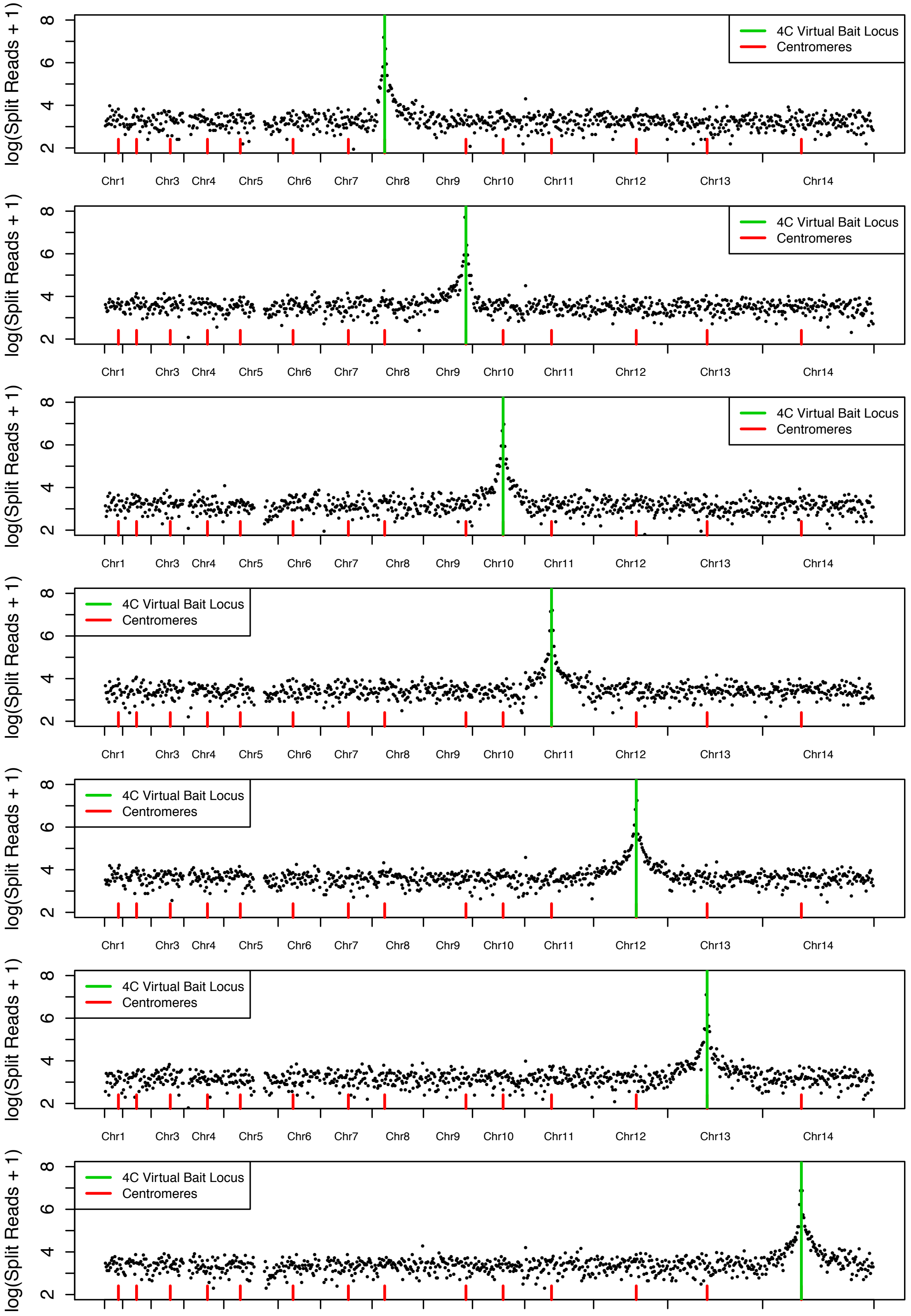

log(Split Reads + 1)
2
4
6
8
4C Virtual Bait Locus
Centromeres
Chr1
Chr3
Chr4
Chr5
Chr6
Chr7
Chr8
Chr9
Chr10
Chr11
Chr12
Chr13
Chr14

Figure S6: Interactions between rDNA loci. A) virtual 4C experiments from the rDNA loci on chromosome 1, 5, and 7. The number of interactions between the locus marked by the green line ("4C virtual bait") and the rest of the genome is plotted as a point for each 25Kb bin in the genome on a log scale. Prominent interactions can be observed between rDNA loci. A-type rDNA are marked with red dashed lines while S-type rDNA are marked with blue dashed lines (only 18S-5.8S-28S clusters of A and S type are marked in the figure; the genome also contains a 5S repeat on chromosome 14 and two incomplete rDNA units on the opposite subtelomeres of chromosome 8). Chromosome boundaries are denoted with grey dashed lines. There is a strong interaction between the A-type loci that are predominantly expressed during the asexual stages. There is also evidence of weaker interaction within the S-type rDNA loci and between A- and S-type loci. The rDNA locus on chromosome 7 interacts with the loci on chromosome 1, 5, 7, 11, and 13 and may therefore function as a spatial "hub" for the nucleolus. **B)** A higher resolution of some of the peaks from Figure 4 showing the increase in signal presence as regional 'peaks' with a distance-dependent decrease in interaction frequency, consistent with a genuine interaction, rather than single bins with isolated increases in interaction. **C)** MboI HiC interaction maps for chromosomes 5, 6, and 7 in IT. The prominent interaction between the rDNA loci on chromosome 5 and 7 can be clearly seen. Chromosome boundaries between chromosomes 5,6, and 7 are noted with a black grid. **D)** Control interaction maps made from digested and then ligated genomic DNA are shown for chromosomes 5, 6, and 7 **E)** Interactions between rDNA loci as shown by normalized plots of split read-pair counts between rDNA loci. In order to reflect genuine physical interactions, the split read-pair counts above background from 50Kb up- and downstream of the 25Kb bins containing rDNA loci were used, with the rDNA-containing bin itself removed from the analysis. **F)** Multidimensional scaling (MDS) plots which show the best-fit spatial relationships among rDNA loci. In this analysis, 1/(split read-pairs) was interpreted as a dissimilarity measure for non-metric MDS, and the best-fit configuration of points was chosen (the relationship between contact probability and spatial distance is not known, which is why non-metric MDS was used). **G)** Virtual 4C plots showing the whole-genome interaction profiles of the 14 centromeres.

Figure S7

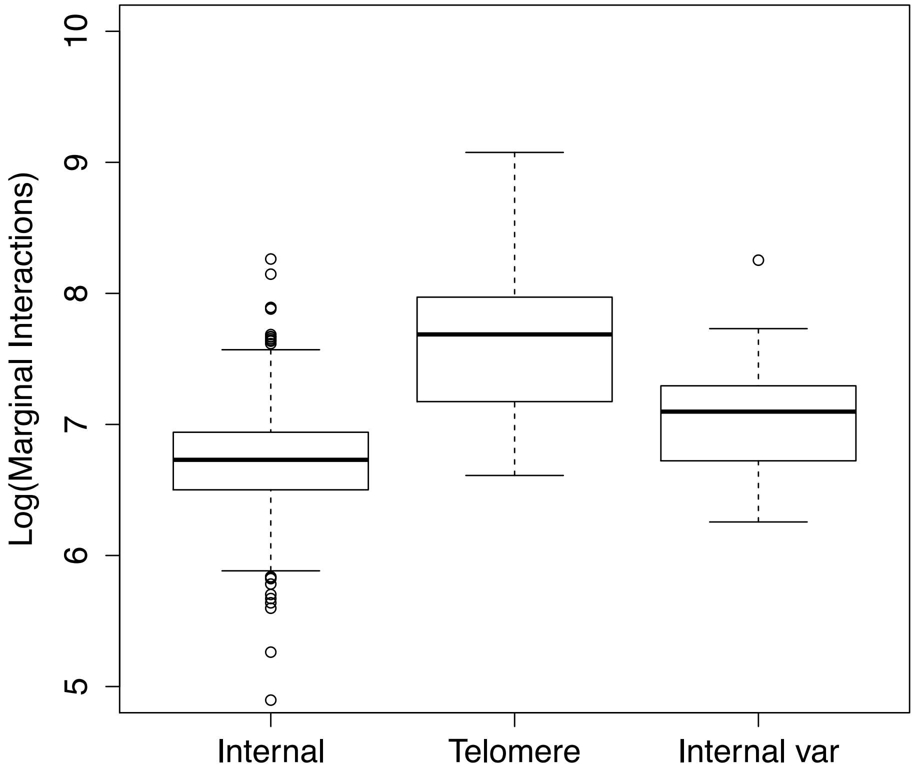

Figure S7: Chromosome region interaction densities. Row (and also, because of symmetry, column) sums of the interaction matrix provide summary statistics of the "total" interaction in a given bin. Stratifying the summed interactions by their presence in internal chromosome regions ("internal") vs. telomeric regions ("telomere") or regions containing internal *var* clusters ("internal var") shows a much higher level of total interaction in these latter regions (internal vs. telomere, Wilcoxon Rank Sum test, $P < 2.2 \times 10^{-16}$; internal vs. internal var, $P = 0.002$, Wilcoxon Rank Sum test). Both telomeres and internal *var* clusters are known sites of heterochromatin formation (Lopez-Rubio et al., 2009). In this case, the 'corrected' interaction matrix was used which was obtained by subtracting the normalized values in the control libraries from the values in the interaction libraries (similar results were obtained for the 'filtered' matrices as well as matrices obtained by discarding non-uniquely aligning reads).

Figure S8

A)

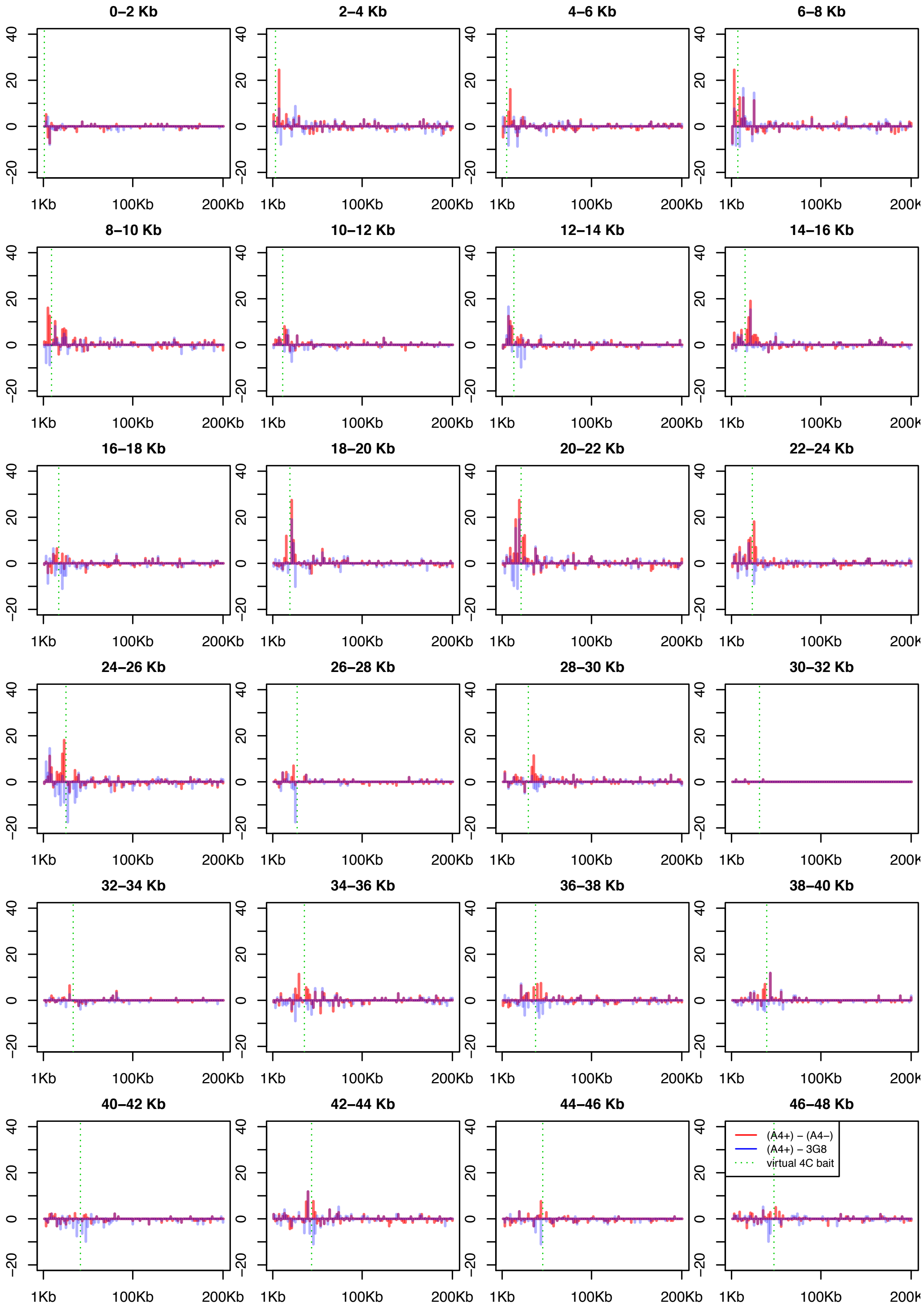
0–2 Kb
2–4 Kb
4–6 Kb
6–8 Kb
8–10 Kb
10–12 Kb
12–14 Kb
14–16 Kb
16–18 Kb
18–20 Kb
20–22 Kb
22–24 Kb
24–26 Kb
26–28 Kb
28–30 Kb
30–32 Kb
32–34 Kb
34–36 Kb
36–38 Kb
38–40 Kb
40–42 Kb
42–44 Kb
44–46 Kb
46–48 Kb
40
20
0
−20
1Kb
100Kb
200Kb
(A4+) − (A4−)
(A4+) − 3G8
virtual 4C bait

B)

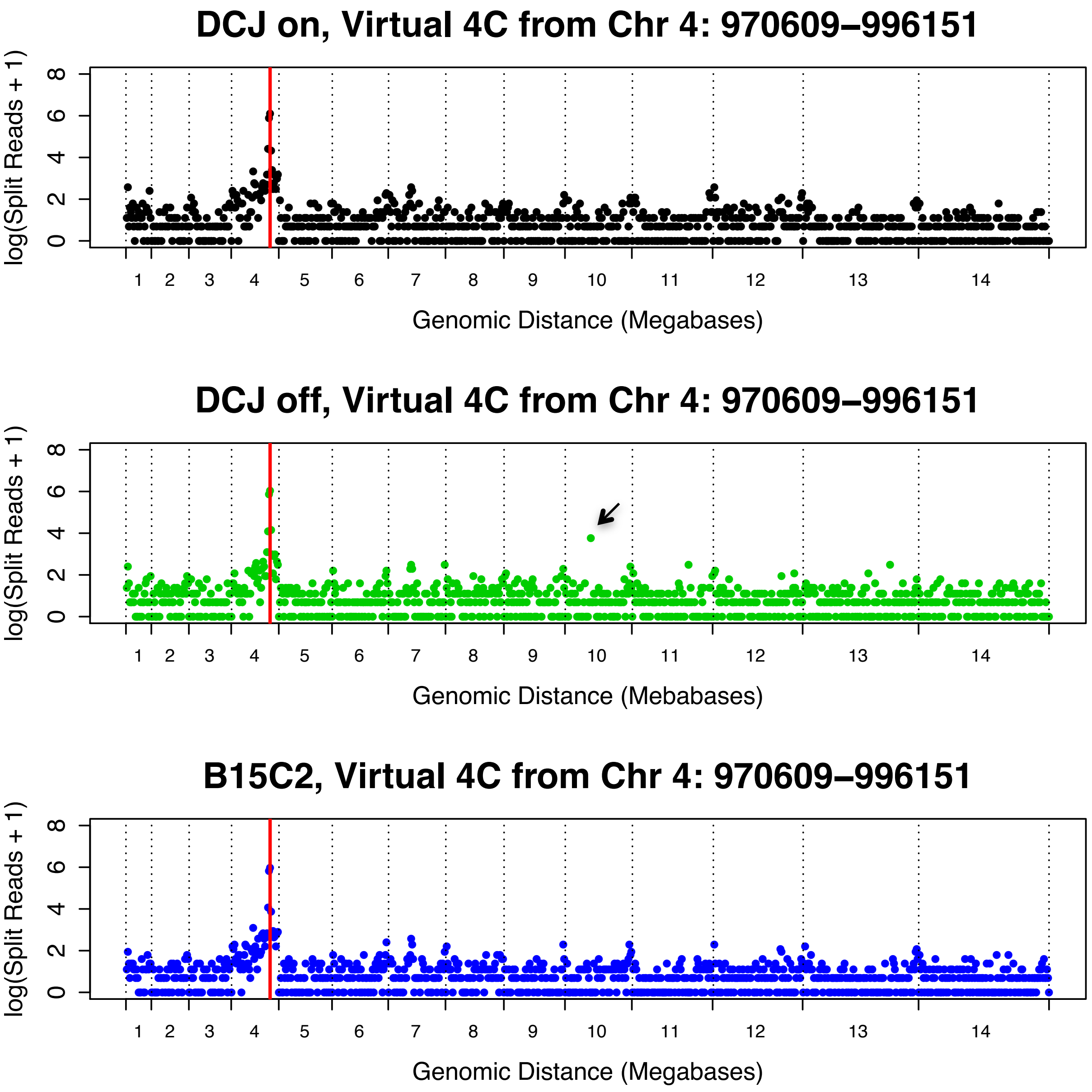
DCJ on, Virtual 4C from Chr 4: 970609–996151
log(Split Reads + 1)
0 2 4 6 8
1 2 3 4 5 6 7 8 9 10 11 12 13 14
Genomic Distance (Megabases)
DCJ off, Virtual 4C from Chr 4: 970609–996151
log(Split Reads + 1)
0 2 4 6 8
1 2 3 4 5 6 7 8 9 10 11 12 13 14
Genomic Distance (Mebabases)
B15C2, Virtual 4C from Chr 4: 970609–996151
log(Split Reads + 1)
0 2 4 6 8
1 2 3 4 5 6 7 8 9 10 11 12 13 14
Genomic Distance (Megabases)

C)

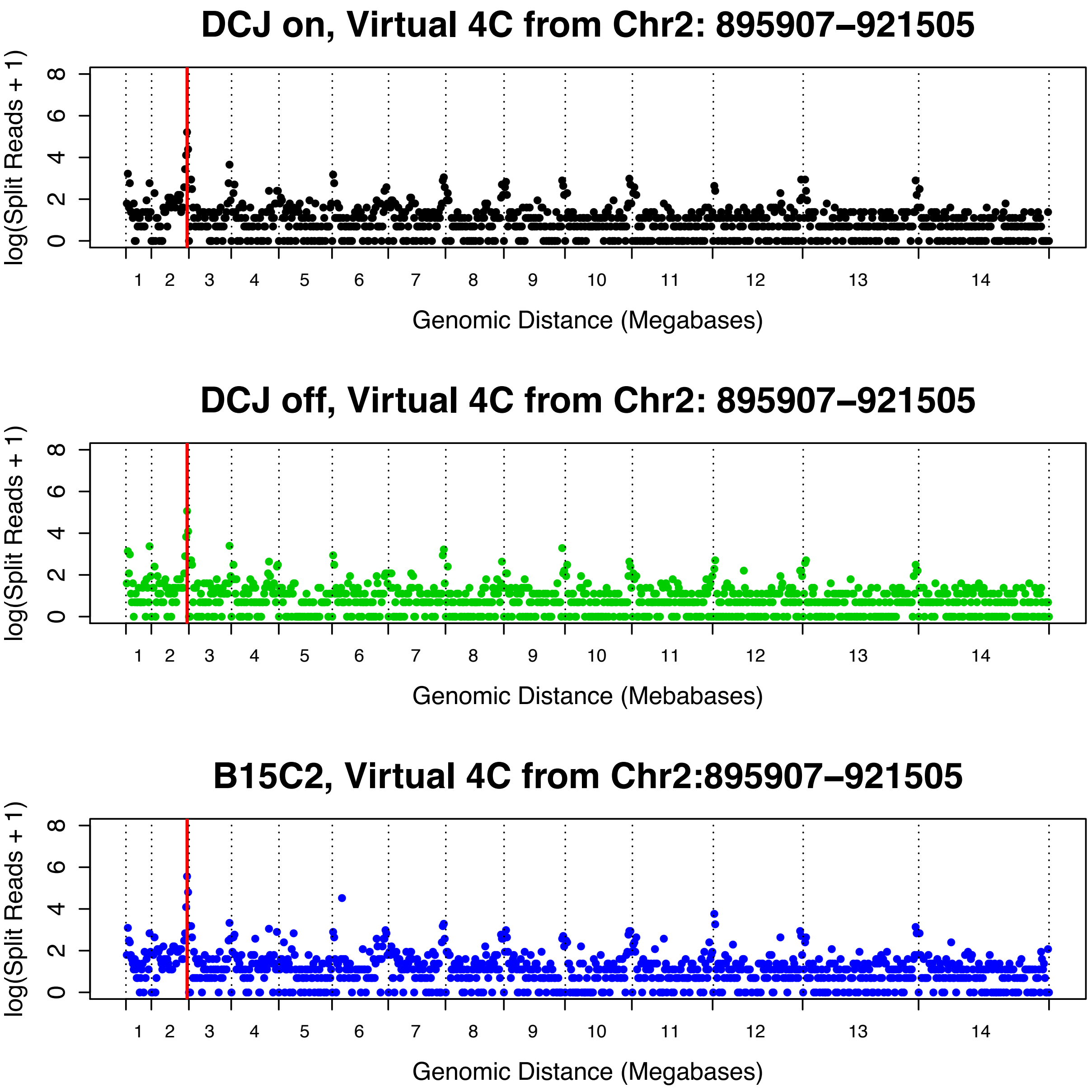
DCJ on, Virtual 4C from Chr2: 895907–921505
log(Split Reads + 1)
0
2
4
6
8
1 2 3 4 5 6 7 8 9 10 11 12 13 14
Genomic Distance (Megabases)
DCJ off, Virtual 4C from Chr2: 895907–921505
log(Split Reads + 1)
0
2
4
6
8
1 2 3 4 5 6 7 8 9 10 11 12 13 14
Genomic Distance (Mebabases)
B15C2, Virtual 4C from Chr2:895907–921505
log(Split Reads + 1)
0
2
4
6
8
1 2 3 4 5 6 7 8 9 10 11 12 13 14
Genomic Distance (Megabases)

D)

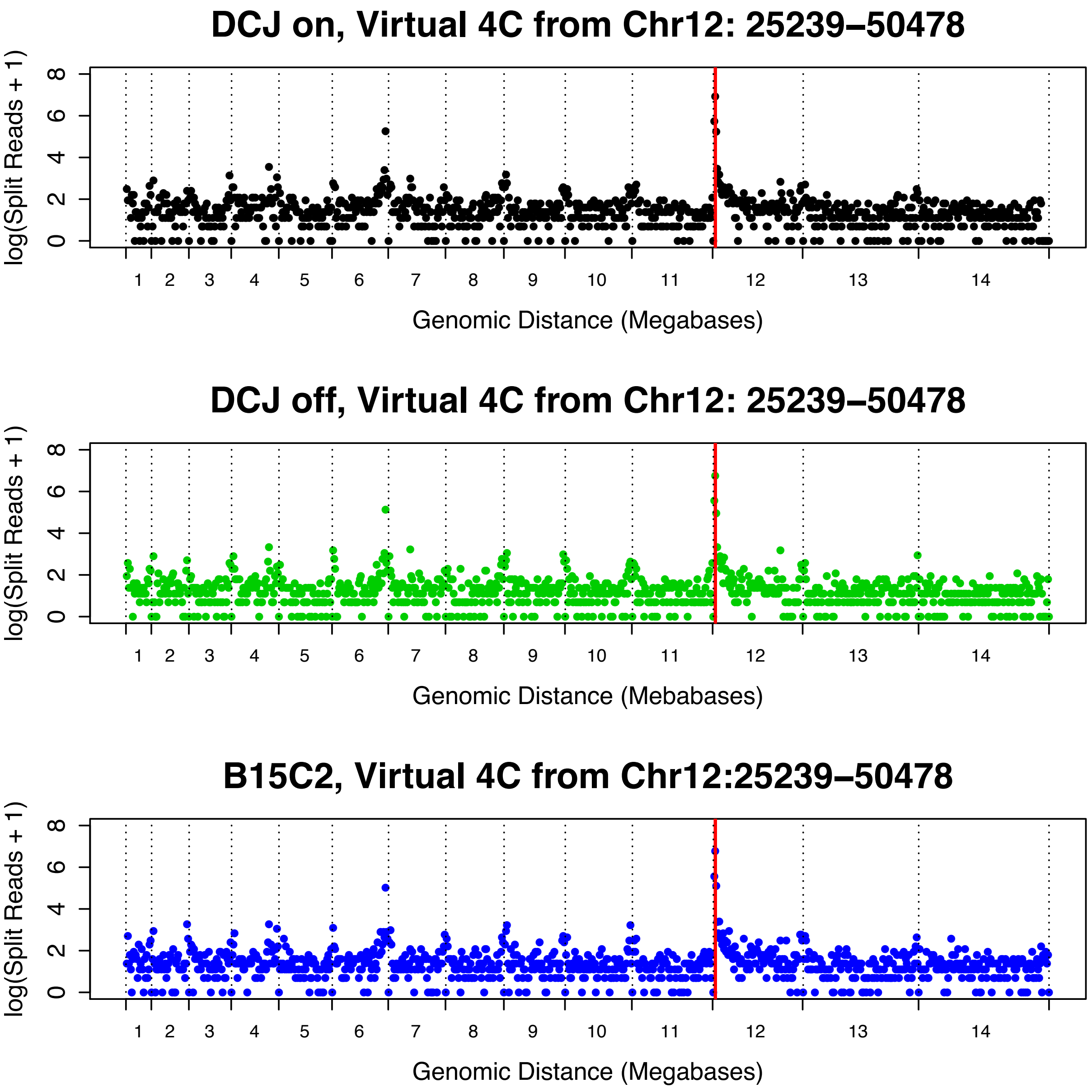
DCJ on, Virtual 4C from Chr12: 25239–50478
log(Split Reads + 1)
0
2
4
6
8
1
2
3
4
5
6
7
8
9
10
11
12
13
14
Genomic Distance (Megabases)
DCJ off, Virtual 4C from Chr12: 25239–50478
Genomic Distance (Mebabases)
B15C2, Virtual 4C from Chr12:25239–50478
Genomic Distance (Megabases)

E)

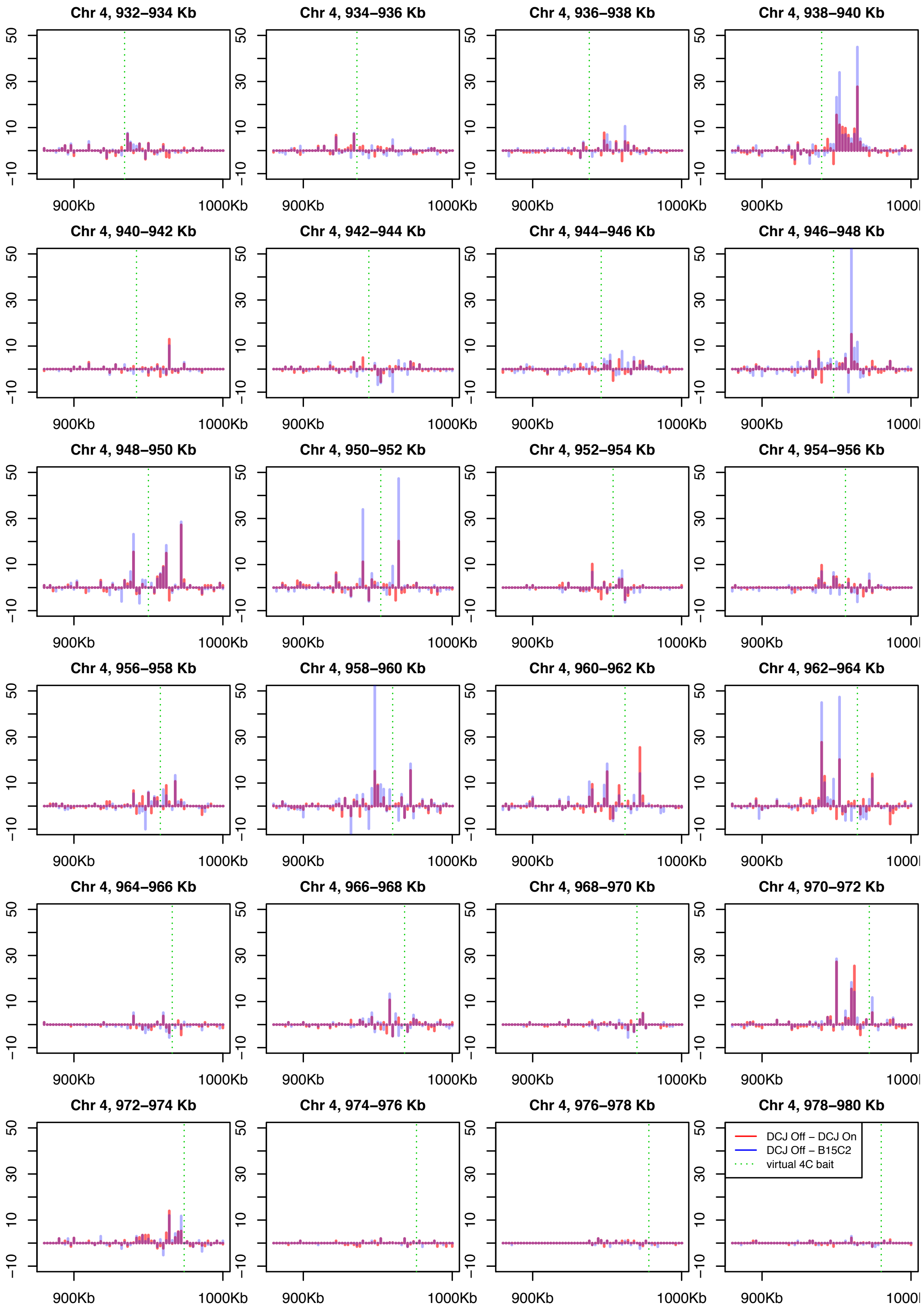
Chr 4, 932–934 Kb
Chr 4, 934–936 Kb
Chr 4, 936–938 Kb
Chr 4, 938–940 Kb
Chr 4, 940–942 Kb
Chr 4, 942–944 Kb
Chr 4, 944–946 Kb
Chr 4, 946–948 Kb
Chr 4, 948–950 Kb
Chr 4, 950–952 Kb
Chr 4, 952–954 Kb
Chr 4, 954–956 Kb
Chr 4, 956–958 Kb
Chr 4, 958–960 Kb
Chr 4, 960–962 Kb
Chr 4, 962–964 Kb
Chr 4, 964–966 Kb
Chr 4, 966–968 Kb
Chr 4, 968–970 Kb
Chr 4, 970–972 Kb
Chr 4, 972–974 Kb
Chr 4, 974–976 Kb
Chr 4, 976–978 Kb
Chr 4, 978–980 Kb
50
30
10
−10
900Kb
1000Kb
DCJ Off − DCJ On
DCJ Off − B15C2
virtual 4C bait

F)

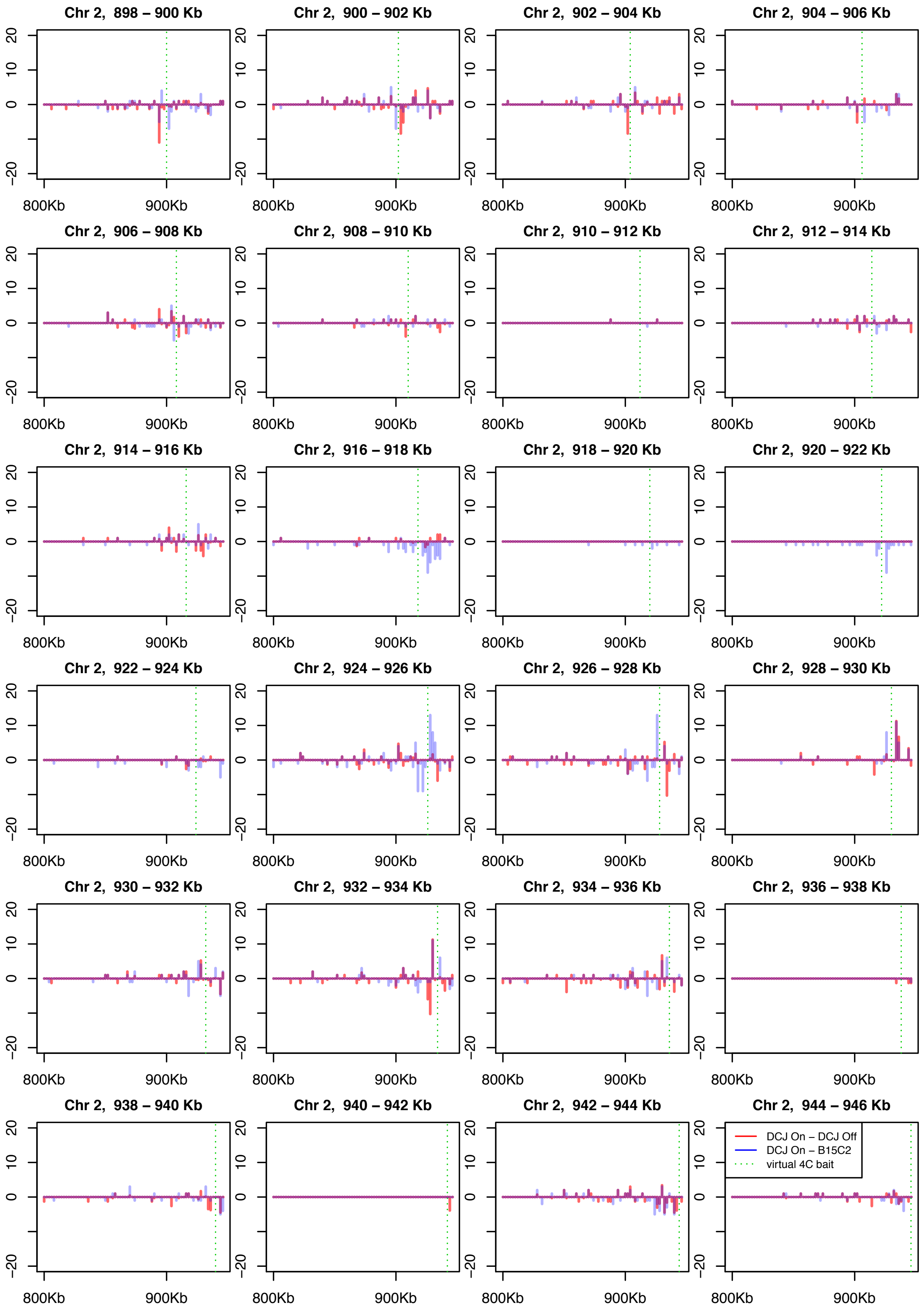

Chr 2, 898 – 900 Kb
Chr 2, 900 – 902 Kb
Chr 2, 902 – 904 Kb
Chr 2, 904 – 906 Kb
Chr 2, 906 – 908 Kb
Chr 2, 908 – 910 Kb
Chr 2, 910 – 912 Kb
Chr 2, 912 – 914 Kb
Chr 2, 914 – 916 Kb
Chr 2, 916 – 918 Kb
Chr 2, 918 – 920 Kb
Chr 2, 920 – 922 Kb
Chr 2, 922 – 924 Kb
Chr 2, 924 – 926 Kb
Chr 2, 926 – 928 Kb
Chr 2, 928 – 930 Kb
Chr 2, 930 – 932 Kb
Chr 2, 932 – 934 Kb
Chr 2, 934 – 936 Kb
Chr 2, 936 – 938 Kb
Chr 2, 938 – 940 Kb
Chr 2, 940 – 942 Kb
Chr 2, 942 – 944 Kb
Chr 2, 944 – 946 Kb
20
10
0
−20
800Kb
900Kb
DCJ On – DCJ Off
DCJ On – B15C2
virtual 4C bait

G)

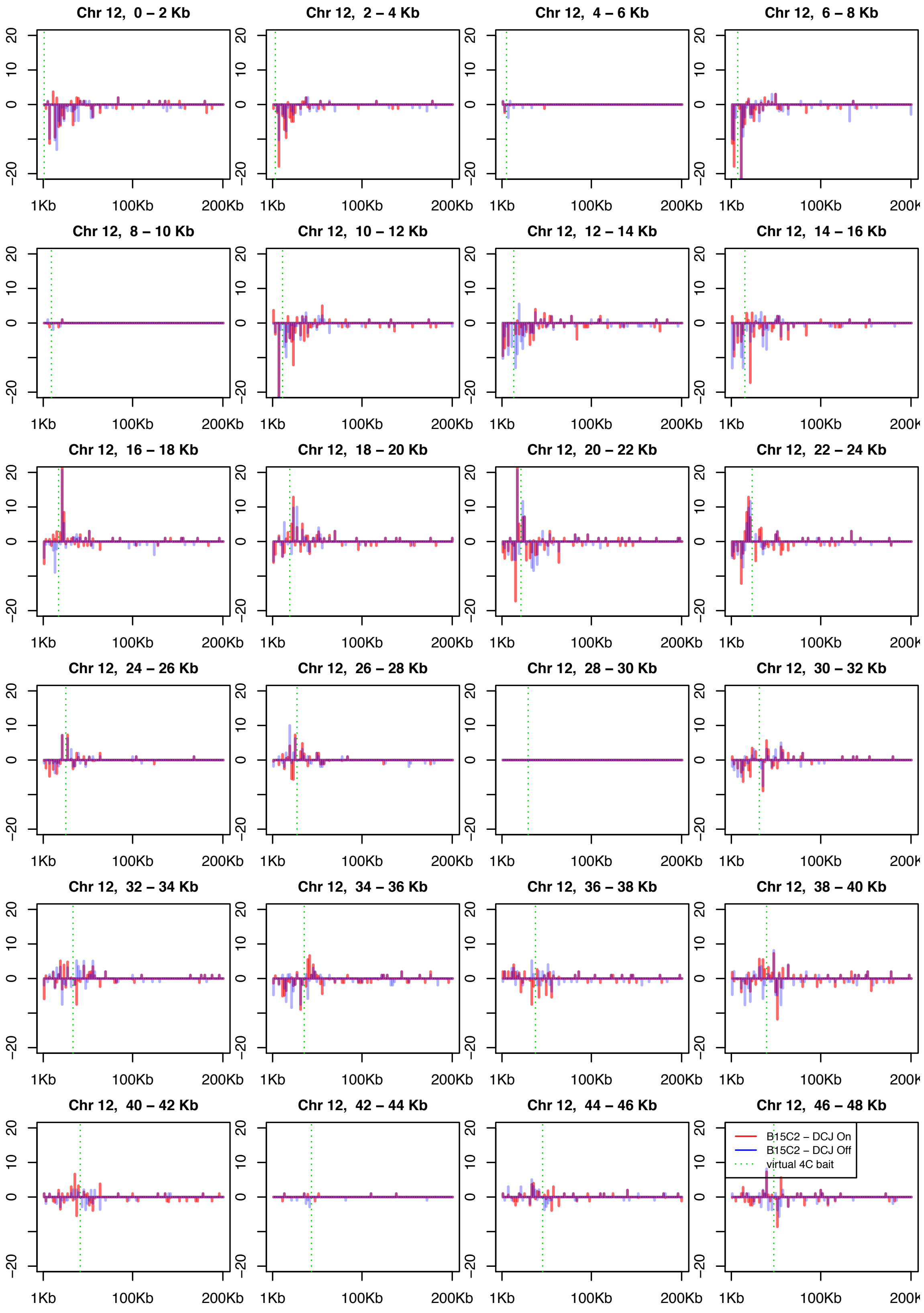

Chr 12, 0 – 2 Kb
Chr 12, 2 – 4 Kb
Chr 12, 4 – 6 Kb
Chr 12, 6 – 8 Kb
Chr 12, 8 – 10 Kb
Chr 12, 10 – 12 Kb
Chr 12, 12 – 14 Kb
Chr 12, 14 – 16 Kb
Chr 12, 16 – 18 Kb
Chr 12, 18 – 20 Kb
Chr 12, 20 – 22 Kb
Chr 12, 22 – 24 Kb
Chr 12, 24 – 26 Kb
Chr 12, 26 – 28 Kb
Chr 12, 28 – 30 Kb
Chr 12, 30 – 32 Kb
Chr 12, 32 – 34 Kb
Chr 12, 34 – 36 Kb
Chr 12, 36 – 38 Kb
Chr 12, 38 – 40 Kb
Chr 12, 40 – 42 Kb
Chr 12, 42 – 44 Kb
Chr 12, 44 – 46 Kb
Chr 12, 46 – 48 Kb
20
10
0
−20
1Kb
100Kb
200Kb
B15C2 – DCJ On
B15C2 – DCJ Off
virtual 4C bait

H)

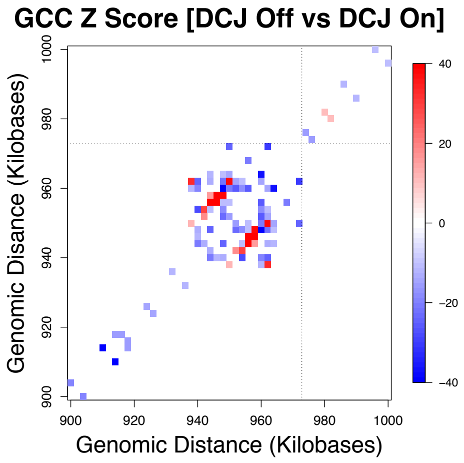
GCC Z Score [DCJ Off vs DCJ On]
Genomic Disance (Kilobases)
Genomic Distance (Kilobases)

I)

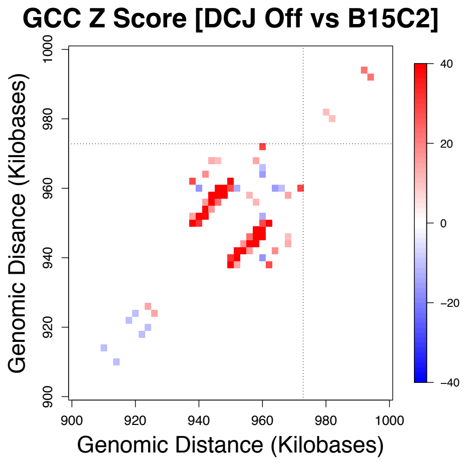
GCC Z Score [DCJ Off vs B15C2]
Genomic Disance (Kilobases)
Genomic Distance (Kilobases)

J)

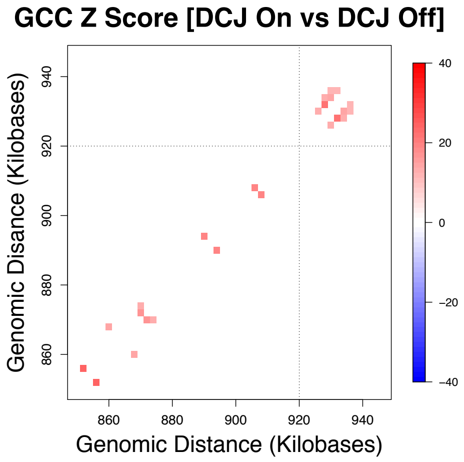
GCC Z Score [DCJ On vs DCJ Off]
Genomic Disance (Kilobases)
Genomic Distance (Kilobases)

K)

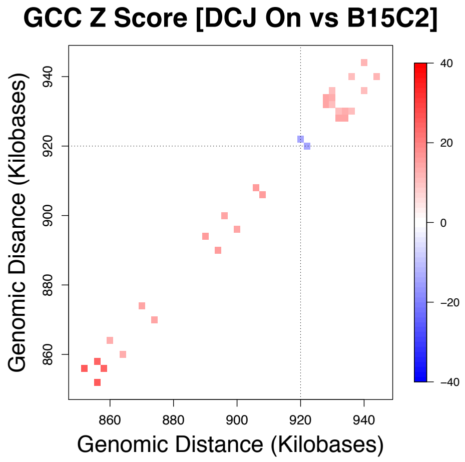
GCC Z Score [DCJ On vs B15C2]
Genomic Disance (Kilobases)
Genomic Distance (Kilobases)

I)

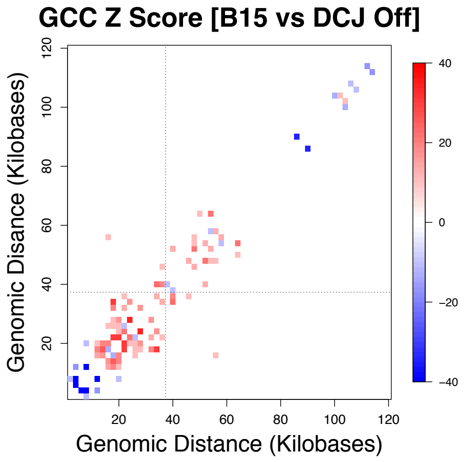
GCC Z Score [B15 vs DCJ Off]
Genomic Disance (Kilobases)
Genomic Distance (Kilobases)

L)

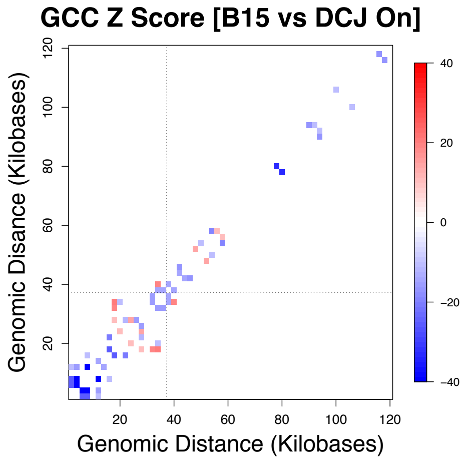
GCC Z Score [B15 vs DCJ On]
Genomic Disance (Kilobases)
Genomic Distance (Kilobases)

M)

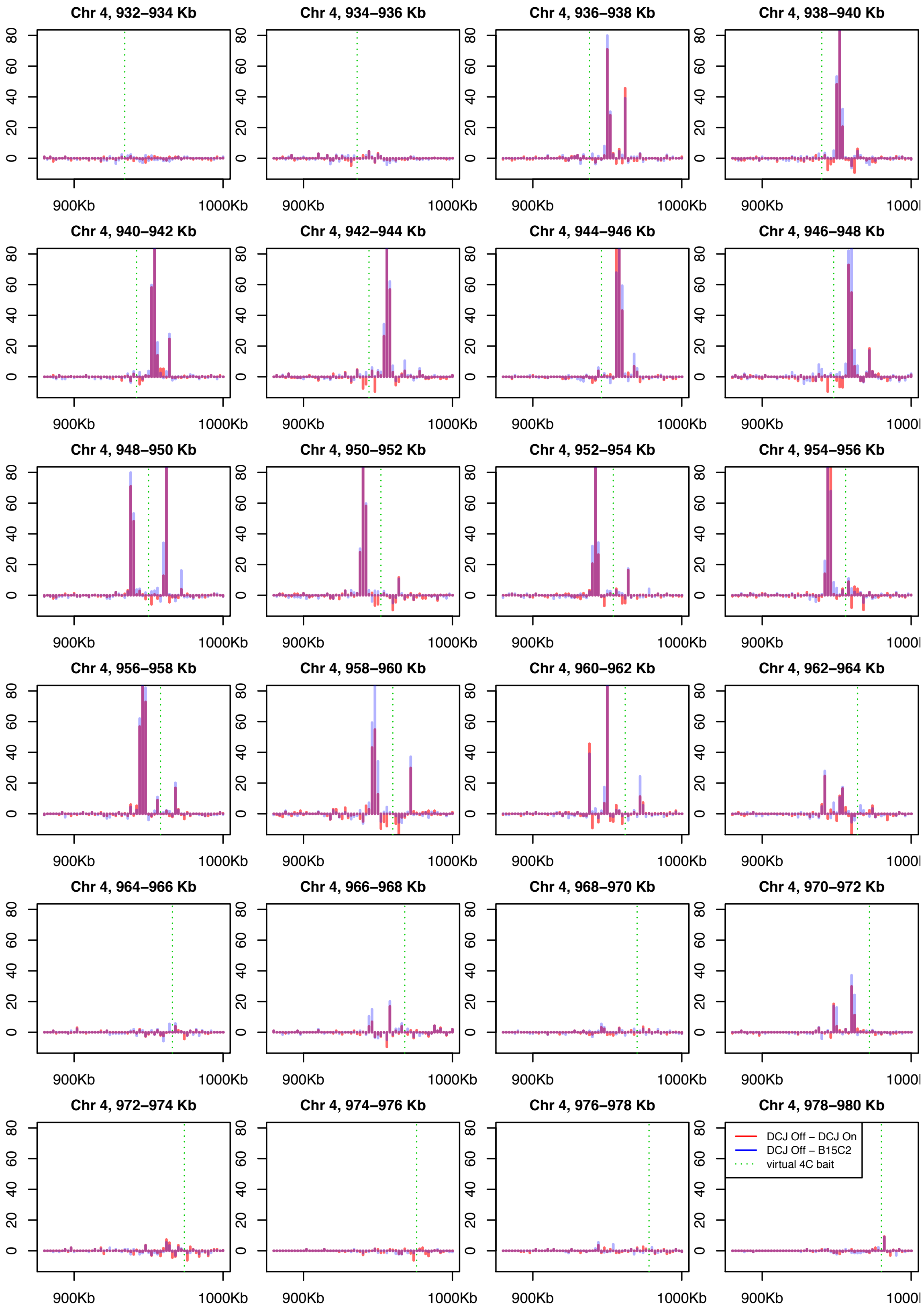
Chr 4, 932–934 Kb
Chr 4, 934–936 Kb
Chr 4, 936–938 Kb
Chr 4, 938–940 Kb
Chr 4, 940–942 Kb
Chr 4, 942–944 Kb
Chr 4, 944–946 Kb
Chr 4, 946–948 Kb
Chr 4, 948–950 Kb
Chr 4, 950–952 Kb
Chr 4, 952–954 Kb
Chr 4, 954–956 Kb
Chr 4, 956–958 Kb
Chr 4, 958–960 Kb
Chr 4, 960–962 Kb
Chr 4, 962–964 Kb
Chr 4, 964–966 Kb
Chr 4, 966–968 Kb
Chr 4, 968–970 Kb
Chr 4, 970–972 Kb
Chr 4, 972–974 Kb
Chr 4, 974–976 Kb
Chr 4, 976–978 Kb
Chr 4, 978–980 Kb
0
20
40
60
80
900Kb
1000Kb
DCJ Off – DCJ On
DCJ Off – B15C2
virtual 4C bait

N)

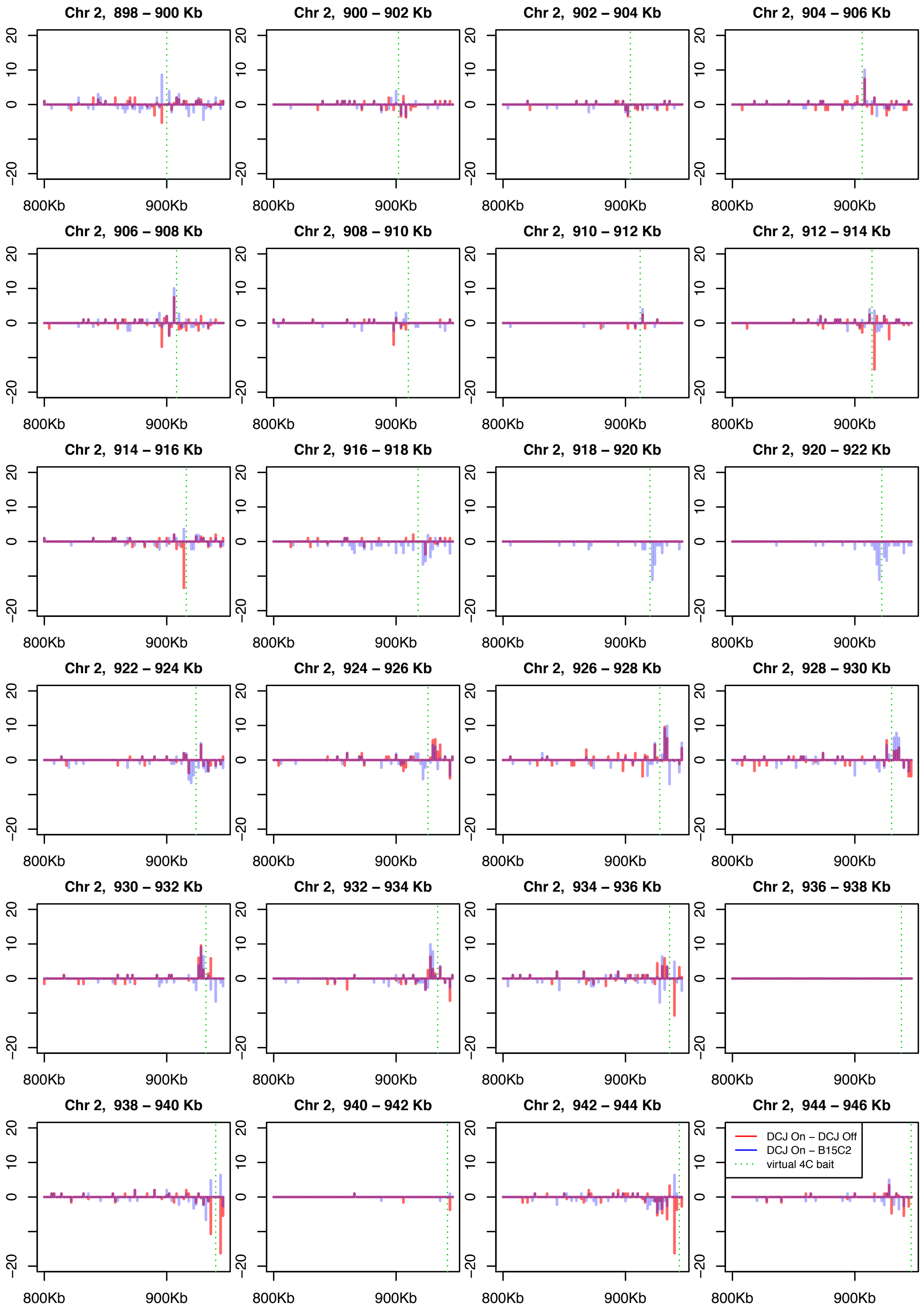
Chr 2, 898 – 900 Kb
Chr 2, 900 – 902 Kb
Chr 2, 902 – 904 Kb
Chr 2, 904 – 906 Kb
Chr 2, 906 – 908 Kb
Chr 2, 908 – 910 Kb
Chr 2, 910 – 912 Kb
Chr 2, 912 – 914 Kb
Chr 2, 914 – 916 Kb
Chr 2, 916 – 918 Kb
Chr 2, 918 – 920 Kb
Chr 2, 920 – 922 Kb
Chr 2, 922 – 924 Kb
Chr 2, 924 – 926 Kb
Chr 2, 926 – 928 Kb
Chr 2, 928 – 930 Kb
Chr 2, 930 – 932 Kb
Chr 2, 932 – 934 Kb
Chr 2, 934 – 936 Kb
Chr 2, 936 – 938 Kb
Chr 2, 938 – 940 Kb
Chr 2, 940 – 942 Kb
Chr 2, 942 – 944 Kb
Chr 2, 944 – 946 Kb
20
10
0
−20
800Kb
900Kb
DCJ On – DCJ Off
DCJ On – B15C2
virtual 4C bait

O)

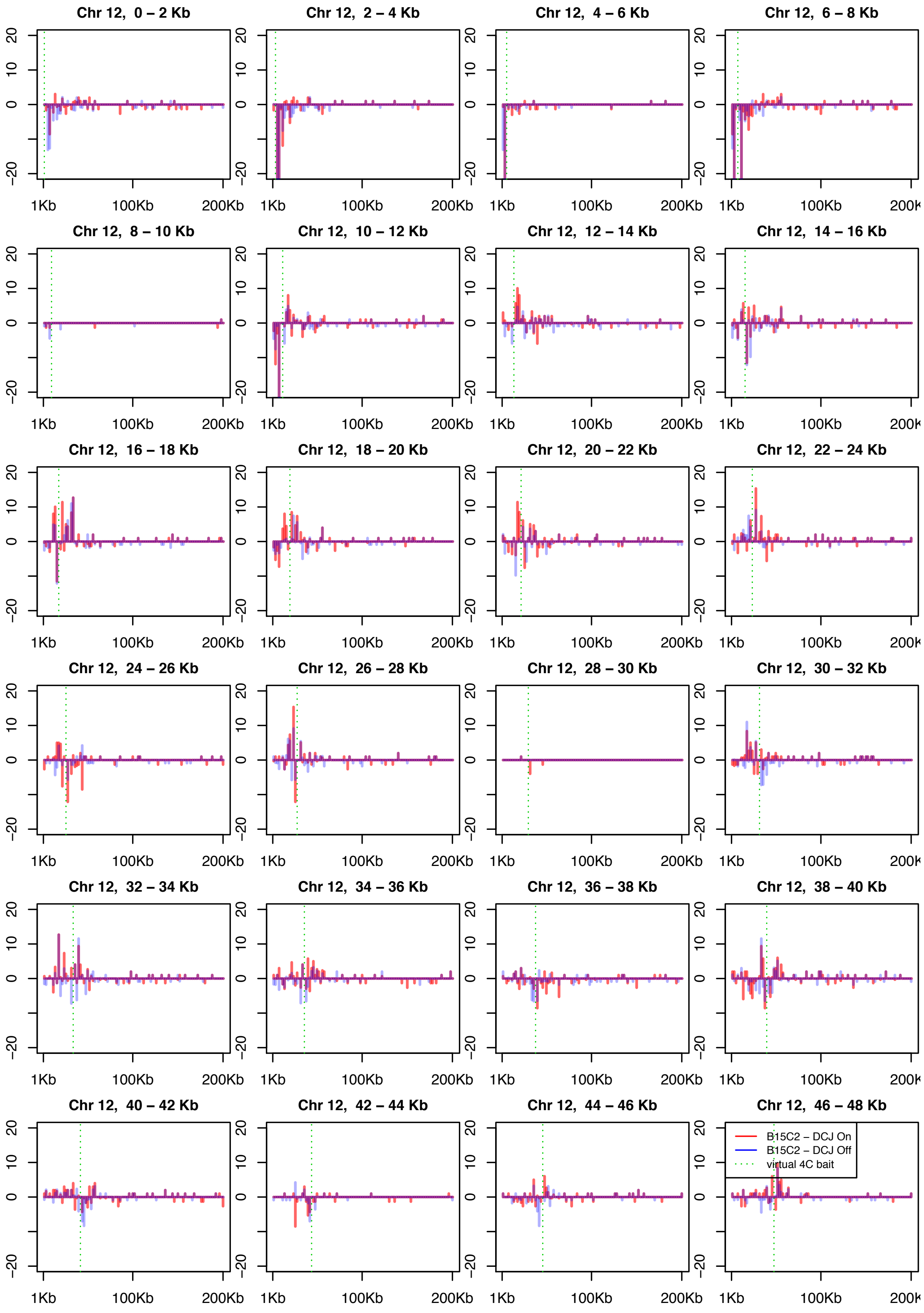
Chr 12, 0 – 2 Kb
Chr 12, 2 – 4 Kb
Chr 12, 4 – 6 Kb
Chr 12, 6 – 8 Kb
Chr 12, 8 – 10 Kb
Chr 12, 10 – 12 Kb
Chr 12, 12 – 14 Kb
Chr 12, 14 – 16 Kb
Chr 12, 16 – 18 Kb
Chr 12, 18 – 20 Kb
Chr 12, 20 – 22 Kb
Chr 12, 22 – 24 Kb
Chr 12, 24 – 26 Kb
Chr 12, 26 – 28 Kb
Chr 12, 28 – 30 Kb
Chr 12, 30 – 32 Kb
Chr 12, 32 – 34 Kb
Chr 12, 34 – 36 Kb
Chr 12, 36 – 38 Kb
Chr 12, 38 – 40 Kb
Chr 12, 40 – 42 Kb
Chr 12, 42 – 44 Kb
Chr 12, 44 – 46 Kb
Chr 12, 46 – 48 Kb
B15C2 – DCJ On
B15C2 – DCJ Off
virtual 4C bait
20
10
0
−20
1Kb
100Kb
200Kb

Figure S8: Local interaction alterations associated with *var* switching in the A4 lines, and local and global interaction alterations associated with *var* switching for the other three *var* genes studied in NF54-derived lines. **A)** The virtual 4C plots for the first 200 KB of chromosome 13 are shown for each of the 2KB bins in the first 48KB of the chromosome (the region containing the A4var locus). The red line shows the difference in normalized read count between the A4+ and A4- lines; the blue line shows the difference in normalized read count between the A4+ and BC6- line. The dashed, green, vertical line marks the site whose interactions are being assessed (the virtual 4C 'bait' locus). **B-D)** The global interaction profile for the 25KB bin containing the activated *var* genes (PF3D7_0421300 in B); PF3D7_0223500 in C); PF3D7_1200400 in D)) is shown as virtual 4C plots. No major differences can be seen globally between the interaction profiles of parasites with activated and silent *var* genes. A single bin on chromosome 10 (arrow in panel **B**) appeared elevated in the DCJ Off line (which expresses PF3D7_0421300), however, this apparent interaction did not display the expected decrease in interaction probability as a function of distance, and was isolated to a small sub-region of the bin, suggesting it was an artifact of sequencing. The DCJ Off MboI HiC library had a number of such bins, which appeared arbitrarily in small numbers in some chromosomes, and appeared to be an artifact of sequencing that affected only this library. **E – G)** Virtual 4C plots showing the difference in local signal in each of the 2KB bins in the 48KB interval containing the *var* locus studied. PF3D7_0421300 and chromosome 4 in **E**); PF3D7_0223500 and chromosome 2 in in **F**); PF3D7_1200400 and chromosome 12 in **G**). In each case, as indicated in the legend, the normalized interaction counts in the lines with the inactivated *var* gene have been subtracted from the normalized counts in the line with the active *var* gene. **H) – L)** Interaction alterations associated with *var* switching in GCC libraries. The plots are the same as Figure 7 B-C, E-F, and H-I, except with GCC libraries instead of HiC libraries. The interaction matrices for individual libraries were subtracted, and the difference was standardized to generate a Z-score. Chromosomal elements that show an increase in interaction are drawn with red links, while areas that decrease in interaction are drawn with blue links. **M) – O)** Same as **E-G**), except with the GCC libraries instead of the HiC libraries: Virtual 4C plots showing the difference in local signal in each of the 2KB bins in the 48KB interval containing the *var* locus studied. PF3D7_0421300 and chromosome 4 in **M**); PF3D7_0223500 and chromosome 2 in in **N**); PF3D7_1200400 and chromosome 12 in **O**). In each case, as indicated in the legend, the normalized interaction counts in the lines with the inactivated *var* gene have been subtracted from the normalized counts in the line with the active *var* gene.

Figure S9

A)

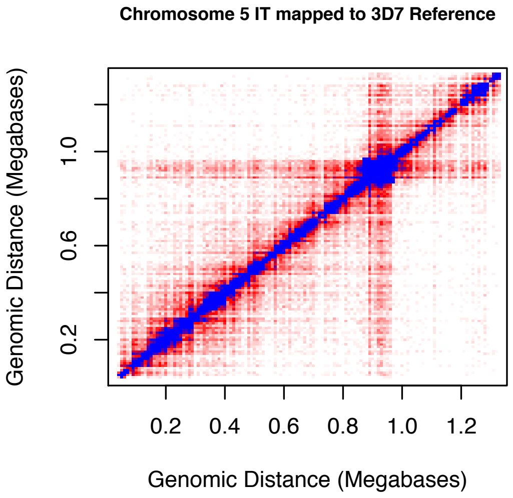

B)

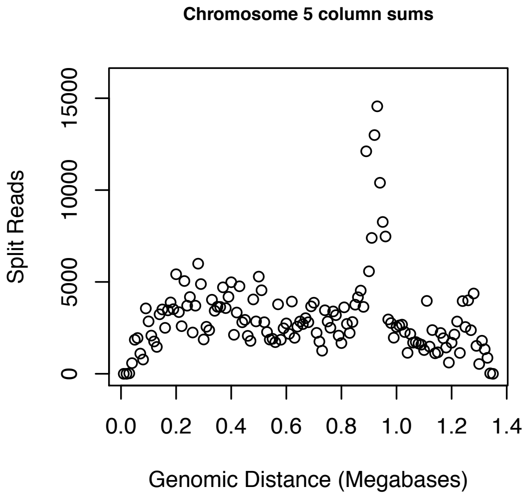

C)

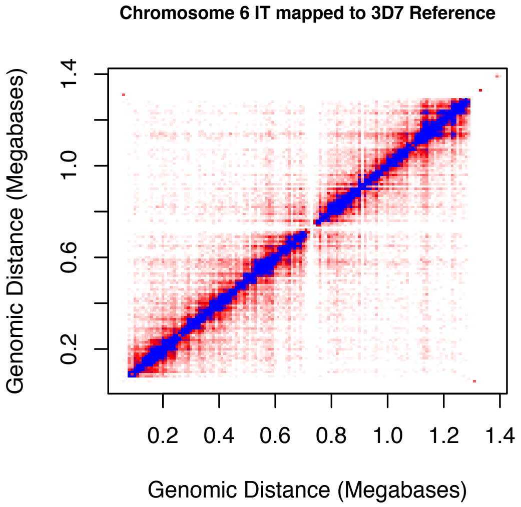

D)

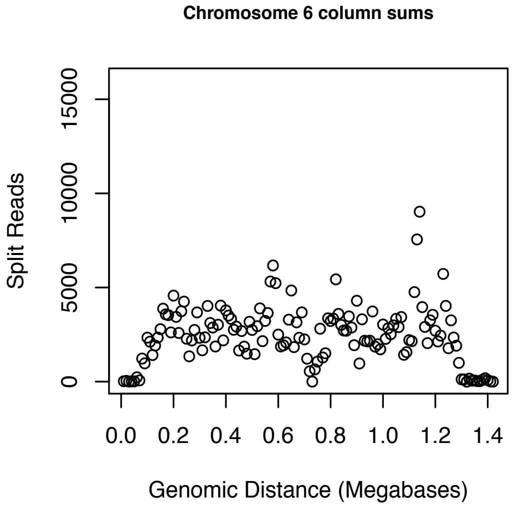

E)

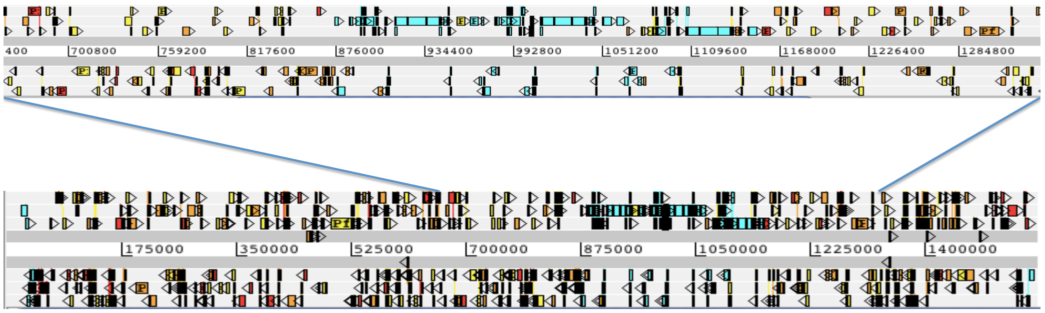

F)

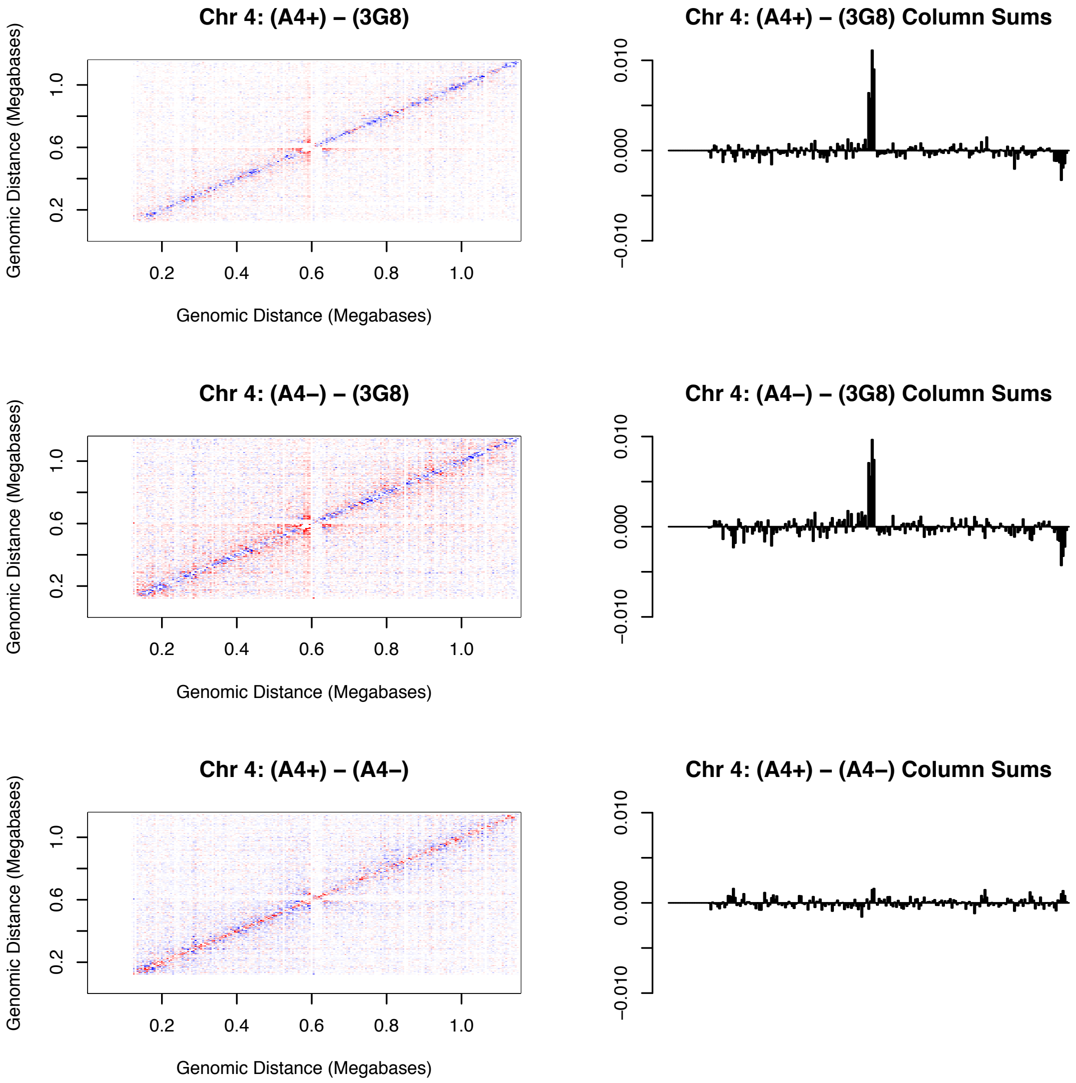

Chr 4: (A4+) – (3G8)
Chr 4: (A4+) – (3G8) Column Sums
Chr 4: (A4–) – (3G8)
Chr 4: (A4–) – (3G8) Column Sums
Chr 4: (A4+) – (A4–)
Chr 4: (A4+) – (A4–) Column Sums
Genomic Distance (Megabases)
0.2
0.4
0.6
0.8
1.0
0.010
0.000
–0.010

G)

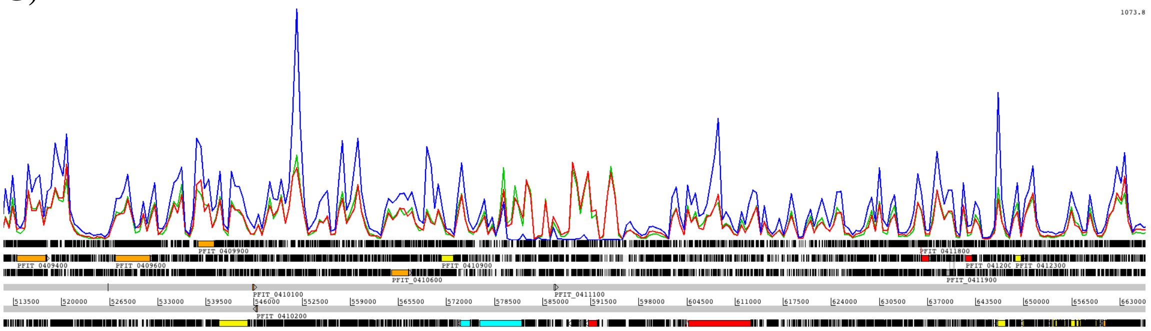

Figure S9): Identification of duplication and deletion events using interaction data. Initial analysis of interaction data from the IT isolate yielded a region of apparently increased interaction frequency between approximately 0.85Mb and 1Mb on chromosome 5. The initial analysis of the IT strain data was performed using a 3D7 reference genome 'morphed' to resemble the IT genome using the iCORN algorithm (Otto et al., 2010). Chromosome interaction matrices using an MboIHiC protocol are shown for Chromosome 5 **(A)**, and the corresponding column sums of this matrix are shown at right in **(B)**. Intra-chromosomal interaction maps and column sums are shown for Chromosome 6 for comparison in **(C)** and **(D)**. In this initial analysis, on chromosome 5, a prominent band of interaction can be observed spanning a 100 Kb region from approximately 0.86 Mb – 0.96 Mb. This region of elevated interaction frequency did not show the expected decrease in interaction over linear genomic distance, and the interaction density extended, with a uniform density, onto other chromosomes in the whole genome map (data not shown), suggesting it was an artifact of the underlying reference sequence and not a genuine interaction since simultaneous interaction with all parts of the genome is physically unlikely. This is a commonly duplicated region that contains the MDR1 gene. Since MDR1 amplifications are frequently found in parasite isolates, especially drug resistant strains, we considered the possibility that this increase in interaction probability was an artifact related to duplication of the underlying sequence. We tested this by constructing large-insert libraries of the IT isolate in order to improve the reference genome and found that the locus was present in three copies. This was confirmed by qPCR data (not shown). A view of the revised locus, showing the amplification event, is given in **(E)**. Similarly, in comparing the interactions in the A4 clone to the 3G8 clone we detected an apparent increase in interaction frequency in the internal antigenic cluster. All libraries from strains of the A4 background showed an apparent increase in interaction density at this site, whereas libraries from the 3G8 subclone showed an apparent decrease in interaction density at this site **(F)**. The heatmaps in **F)** show the difference between the normalized interaction maps between libraries. For example, the plot entitled Chr4: (A4+) – (3G8) plots the normalized heatmap of A4+ selected line at 5Kb resolution with the normalized heatmap of chromosome 4 in 3G8, at the same resolution, subtracted. This analysis yielded a region of apparently increased interaction in the A4+ and A4- lines vs. the 3G8 lines extending over a region of approximately 15Kb. However, examination of the genomic reads in this region, which included non-split read-pairs (i.e. read-pairs that map with the expected insert size), revealed a 15Kb deletion from extending 16.2 Kb from 580,679 bp - 596880 bp **(G)**. All reads (split and non-split) from the MboI HiC libraries are shown mapping to chromosome 4 in Artemis (Rutherford et al., 2000), revealing roughly uniform coverage across the genome in the A4+ library (red line), A4- library (green line), and a drop in coverage over the deleted region in the 3G8 line (blue line).

Cai, W., Aburatani, H., Stanton, V.P., Housman, D.E., Wang, Y.K., and Schwartz, D.C. (1995). Ordered Restriction Endonuclease Maps of Yeast Artificial Chromosomes Created by Optical Mapping on Surfaces. PNAS *92*, 5164–5168.

Colby, R.H., and Rubinstein, M. (2003). Polymer physics (Oxford ;: Oxford University Press).

Deitsch, K.W., del Pinal, A., and Wellems, T.E. (1999). Intra-cluster recombination and var transcription switches in the antigenic variation of Plasmodium falciparum. Molecular and Biochemical Parasitology *101*, 107–116.

Dekker, J., Rippe, K., Dekker, M., and Kleckner, N. (2002). Capturing Chromosome Conformation. Science *295*, 1306–1311.

Dzikowski, R., Frank, M., and Deitsch, K. (2006). Mutually Exclusive Expression of Virulence Genes by Malaria Parasites Is Regulated Independently of Antigen Production. PLoS Pathog *2*, e22.

Flory, P.J. (1953). Principles of Polymer Chemistry (Cornell University Press).

Grosberg, A., Rabin, Y., Havlin, S., and Neer, A. (1993). Crumpled Globule Model of the Three-Dimensional Structure of DNA. Europhysics Letters (EPL) *23*, 373–378.

Grosberg, A.Y., Nechaev, S.K., and Shakhnovich, E.I. (1988). The role of topological constraints in the kinetics of collapse of macromolecules. Journal De Physique *49*, 2095–2100.

Horrocks, P., Kyes, S., Pinches, R., Christodoulou, Z., and Newbold, C. (2004). Transcription of a subtelomerically located var gene variant in Plasmodium falciparum appears to require the truncation of an adjacent var gene. Molecular and Biochemical Parasitology *134*, 193–199.

Lemieux, J.E., Gomez-Escobar, N., Feller, A., Carret, C., Amambua-Ngwa, A., Pinches, R., Day, F., Kyes, S.A., Conway, D.J., Holmes, C.C., et al. (2009). Statistical estimation of cell-cycle progression and lineage commitment in Plasmodium falciparum reveals a homogeneous pattern of transcription in ex vivo culture. Proceedings of the National Academy of Sciences *106*, 7559–7564.

Lieberman-Aiden, E., van Berkum, N.L., Williams, L., Imakaev, M., Ragoczy, T., Telling, A., Amit, I., Lajoie, B.R., Sabo, P.J., Dorschner, M.O., et al. (2009). Comprehensive Mapping of Long-Range Interactions Reveals Folding Principles of the Human Genome. Science *326*, 289 –293.

Lopez-Rubio, J.-J., Mancio-Silva, L., and Scherf, A. (2009). Genome-wide Analysis of Heterochromatin Associates Clonally Variant Gene Regulation with Perinuclear Repressive Centers in Malaria Parasites. Cell Host & Microbe *5*, 179–190.

Mandelbrot, B.B. (1983). The fractal geometry of nature (Macmillan).

Norris, J.R. (1998). Markov Chains (Cambridge University Press).

Otto, T.D., Sanders, M., Berriman, M., and Newbold, C. (2010). Iterative Correction of Reference Nucleotides (iCORN) Using Second Generation Sequencing Technology. Bioinformatics *26*, 1704–1707.

Rodley, C.D.M., Bertels, F., Jones, B., and O'Sullivan, J.M. (2009). Global identification of yeast chromosome interactions using Genome conformation capture. Fungal Genetics and Biology *46*, 879–886.

Rutherford, K., Parkhill, J., Crook, J., Horsnell, T., Rice, P., Rajandream, M.-A., and Barrell, B. (2000). Artemis: Sequence Visualization and Annotation. Bioinformatics *16*, 944–945.

Salanti, A., Staalsoe, T., Lavstsen, T., Jensen, A.T.R., Sowa, M.P.K., Arnot, D.E., Hviid, L., and Theander, T.G. (2003). Selective upregulation of a single distinctly structured var gene in chondroitin sulphate A-adhering Plasmodium falciparum involved in pregnancy-associated malaria. Molecular Microbiology *49*, 179–191.

Yaffe, E., and Tanay, A. (2011). Probabilistic modeling of Hi-C contact maps eliminates systematic biases to characterize global chromosomal architecture. Nature Genetics *43*, 1059–1065.
